# INFORM: A Pediatrician's Communication Curriculum About Diagnostic Conversations in Somatic Symptom and Related Disorders

**DOI:** 10.15766/mep_2374-8265.11561

**Published:** 2025-12-02

**Authors:** Florence Lambert-Fliszar, Catherine Sullivan, Christina Giudice, Hannibal Person

**Affiliations:** 1 Assistant Professor, Division of Pediatric Hospital Medicine, Department of Pediatrics, Geisel School of Medicine at Dartmouth; 2 Division Chief, Division of Pediatric Hospital Medicine, Department of Pediatrics, The Warren Alpert Medical School of Brown University and Hasbro Children's; 3 Advanced Practice Provider, Division of Pediatric Hospital Medicine, Department of Pediatrics, Connecticut Children's; 4 Assistant Professor, Division of Pediatric Gastroenterology and Hepatology, Department of Pediatrics, University of Washington School of Medicine and Seattle Children's Hospital

**Keywords:** Somatic Symptom, Integrated Behavioral Health, Communication Skills, Adolescent Medicine, Pediatrics

## Abstract

**Introduction:**

Somatic symptom and related disorders (SSRDs) is an umbrella term for physical symptoms related to or amplified by psychosocial factors. Clinicians find difficulty in delivering SSRD diagnoses to families. Caregiver buy-in of an SSRD diagnosis is strongly associated with better outcomes. Curricula addressing gaps in this communication skill are lacking, indicating a need for effective educational innovations to increase pediatricians’ confidence and efficacy in SSRD diagnostic conversations with caregivers.

**Methods:**

A 90-minute curriculum composed of didactic and experiential components, including role-play, was designed using Kern's six steps to curriculum development and the INFORM framework (Introduce, Name and Narrate, Feedback, Orient to diagnosis, Reframe, Management principles). We evaluated and improved the curriculum over multiple iterations using the CIPP (Context-Input-Process-Product) model, in which acceptability and feasibility data were collected from participants via quantitative surveys. These surveys also assessed attainment of educational objectives.

**Results:**

A total of 85 responses (of 97 total participants; 88%) were obtained from pediatric residents, pediatric hospital medicine fellows, and pediatric hospitalist attendings. Based on survey responses to two of three true/false questions, participants demonstrated significantly improved knowledge postcurriculum. The proportion of learners reporting feeling confident in SSRD diagnostic conversations increased, from 32% before to 86% after the curriculum. A total of 95% of respondents would recommend this curriculum to a colleague, and 98% reported they would use their skills in future clinical practice.

**Discussion:**

We created a novel, well-received SSRD diagnostic communication curriculum via an iterative process, with demonstration of achieved educational objectives.

## Educational Objectives

By the end of this activity, learners will be able to:

1. Recognize the importance of early diagnostic communication in the treatment of somatic symptom and related disorders (SSRDs).
2. Develop greater confidence in delivering an SSRD diagnosis to a patient's caregivers using the INFORM (Introduce, Name and Narrate, Feedback, Orient to diagnosis, Reframe, Management principles) framework.

## Introduction

A diagnosis of somatic symptom and related disorders (SSRDs) is increasingly prevalent in the pediatric population, yet clinicians have significant misconceptions and knowledge gaps that negatively impact patient care. SSRDs, which most often include pain, neurologic symptoms, and gastrointestinal symptoms,^1^ encompass complex phenomena that are now best described as biopsychosocial in etiology and can cause significant distress and impairment. The manifestation of SSRDs is more severe for those who are hospitalized, and usually begins in childhood.^2^ As a result, SSRDs place a heavy burden on the health care system and account for up to 20% of yearly health care expenditures in the United States.^3^ The effect of having a somatization disorder extends to missed school days, social opportunities, and other activities. Furthermore, clinicians’ negative bias and lack of confidence in diagnosing and managing these disorders may contribute to adverse health care experiences for both the patient and the clinician.^4^ The scope of this problem is vast and an important target for improvement of mental health-related pediatric care. To address knowledge gaps, especially knowing the importance of effective communication in these diagnoses, we developed a novel curriculum for pediatric health care professionals to build skills and a new framework for effective SSRD diagnostic conversations.

Studies suggest that pediatricians do not feel adequately equipped to manage patients with SSRDs. A multicenter study revealed that even though it has been reported that hospitalizations, testing, and procedures can exacerbate an SSRD diagnosis, almost 38% of children had repeat admissions within the year subsequent to diagnosis.^5^ More specifically, Malas et al reported that more than half of primary care providers did not enjoy working with patients with SSRDs and did not feel comfortable discussing management and nonpharmacologic treatment.^6^

To our knowledge, there are no published SSRD curricula that are specific to diagnostic delivery. In the world of SSRDs, there is one curriculum designed to teach pediatric bedside nurses how to manage falls related to this diagnosis.^7^ There are many curricula surrounding patient-centered and empathetic communication skills, such as those seen in the web-based program VitalTalk.^8^ Evidence-based family-centered and empathetic communication techniques^9^ will inform the teachings of our curriculum. However, this curriculum expands on these foundations to address challenges specific to SSRDs, such as appropriate language to use or avoid to progress patient care. Moreover, many of the communication scenarios in existing communication curricula are designed for serious illness or pertain to “breaking bad news,”^10^ including several published in *MedEdPORTAL* specific to pediatrics.^11,12^

Our aim is to provide a more positive framework, given the excellent prognosis of properly treated SSRDs, with the hope that our approach will inspire confidence and optimism in parents and doctors rather than reinforce the misconception that these diagnoses are untreatable. *MedEdPORTAL* also features curricula pertaining to trauma-informed care,^13^ which is relevant to the communication of SSRDs, as well as curricula surrounding pediatric behavioral health, such as management of anxiety and depression.^14^ However, although the pathophysiology of these illnesses can involve a history of adverse childhood experiences and comorbid psychiatric illnesses, these are only small pieces of a larger phenomenon that manifests as physical, often debilitating, symptoms. We teach memorable, organized scripts to accurately depict our current scientific understanding of the biopsychosocial etiology of SSRDs, and to discuss them in a way that does not make patients and parents feel that their symptoms are fabricated, exaggerated, or “in their head.”

When physicians discuss the patient's functional disorder as a diagnosis of exclusion, patients can easily sense uneasiness and hesitation and are therefore less likely to accept the diagnosis.^15^ Delays in diagnostic discussions are problematic because waiting for reassuring workup can decrease the likelihood of parent buy-in and worsen outcomes.^16^ Conversely, studies show that parent buy-in of the diagnoses during hospitalization could improve outcomes by almost eightfold.^17^ What we understand of these disorders is that early recognition and treatment are crucial to recovery, as is involving the family. These findings suggest a diagnostic communication curriculum could be one of the most impactful educational strategies aimed at improving the care of patients with SSRDs.

## Methods

We convened a team of experts in pediatric hospital medicine, functional neurologic disorders, and disorders of gut-brain-interaction (DGBIs) to design this communication curriculum. Specifically, this included a pediatric psychiatrist, a gastroenterologist, and the medical director of the Gut-Brain Health Program, an interdisciplinary clinic for children with DGBIs. It also included two pediatric hospitalists, a physician, and a nurse practitioner, who have developed a novel clinical pathway that standardizes workflow for inpatient SSRD care at their institution.^18^ Finally, a pediatric hospital medicine fellow acted as team lead and provided trainee perspective for the curriculum. Together, we developed the INFORM framework (Introduce, Name and Narrate, Feedback, Orient to diagnosis, Reframe, Management principles) as the basis of the communication curriculum, as a stepwise guide for SSRD diagnostic conversations.

**Figure 1.**
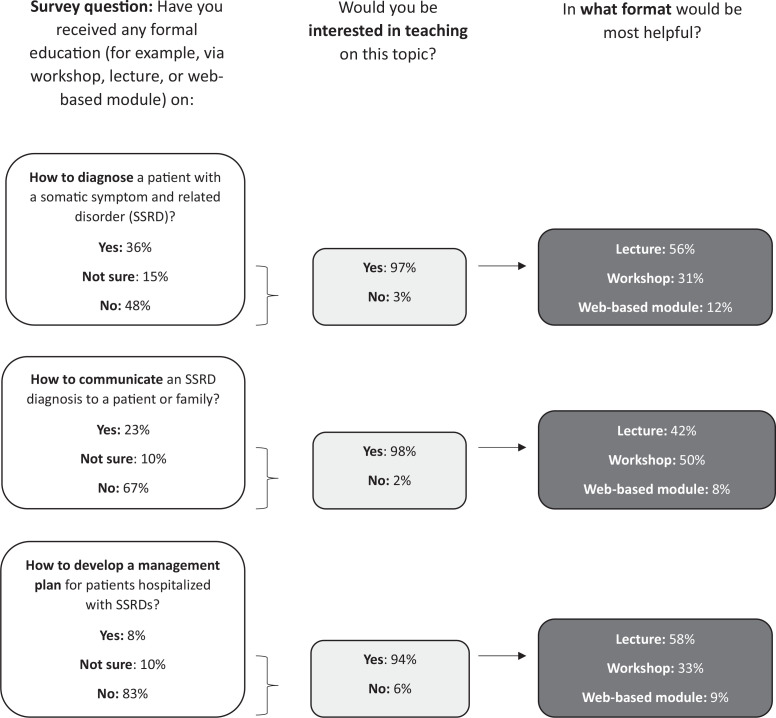
Pediatric resident survey needs assessment responses.

We designed the curriculum using Kern's six steps to curriculum development,^19^ including a targeted needs assessment of pediatric residents conducted via a quantitative survey. We collected data from 52 pediatric residents (34% response) and discovered that diagnostic communication and parent acceptance of the diagnosis are two of the dominant problems residents face in the hospital. Based on responses to the multiple-choice knowledge questions, our survey data suggest that most residents (71%) erroneously viewed SSRDs as a diagnosis of exclusion, and many (20%) believed that it should not be discussed with the family prior to full medical workup. Of the free-text responses, 54% mentioned difficulty with communication with family or parent buy-in as a significant challenge in inpatient SSRD care. Moreover, 67% of residents reported they never received formal education on best communication practices for SSRD diagnostic delivery. Of these, 98% of residents were interested in receiving education on this topic, and 50% of them preferred a workshop format (Figure 1). We used this information to guide the development of our curriculum, specifically prioritizing diagnostic communication for our educational objectives.

The pedagogical approach for this curriculum is experiential learning. In accordance with Kolb's experiential learning model, we believe that learning is a dynamic process beyond the acquisition of knowledge, especially when it comes to communication skills and human interactions that vary from one context to the next. We also aim for tangible behavioral change, which can be better achieved with reflection that can be applied to future active experimentation.^20^ Specifically, our interactive workshop includes demonstration of communication skills, role-playing, and several small- and large-group reflections, wherein less than half of the time is didactic (Appendices A and B). For example, the didactic portion is regularly broken up by large- and small-group reflections, polling questions to the audience, and example role-playing from the facilitators themselves (Appendix C). The majority of the didactic portion is spent detailing the INFORM acronym, including providing analogies to explain functional symptoms, such as the smoke or car alarm.^21^

One of the most effective ways to build communication skills is via simulation or role-play. Role-play can offer a chance to influence participants’ affective domains and behaviors.^22^ However, implementation of role-play can be challenging for several reasons, with participants’ apprehension being one of the most important ones. We mitigated these challenges by appropriately preparing our facilitators, establishing a safe, supportive learning environment, providing structured feedback guidelines, and offering opportunities for repetition.^22^ In our slide deck instructions, we specifically describe what makes role-play challenging, and we set clear group rules, including participants’ option to take pauses as needed (Appendix B). Participants could also choose to participate as observers only. Evidence suggests that observation is also essential in enhancing and reinforcing communication skills.^23^ Finally, we hope that because the facilitators are acting out a case twice during the interactive didactic, this also makes role-play less intimidating.

We formed groups of three to four participants, and each was given clear instructions from the slide presentation to practice with three different sample cases (Appendix D). In the role-play, there are three roles: the clinician, the caregiver, and the observer. The instructions for role-play include example questions that the caregiver may ask in the role-play and specific points for observer feedback (Appendix E) to facilitate discussion. Additionally, participants are provided with example scripts within an INFORM reference sheet to guide their practice during the role-play (Appendix F). Facilitators may reference Appendix G for a list of acronyms used throughout the slide deck (Appendix B) and accompanying materials (Appendices C–F).

### Curriculum Development

**Figure 2.**
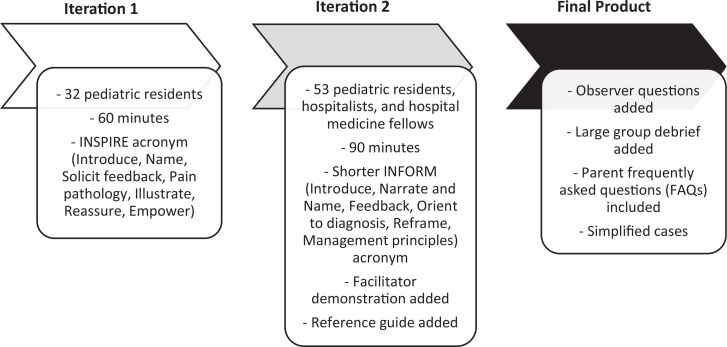
Map of process-based curriculum evaluation. Authors gathered participant feedback and performed facilitator debriefs between each iteration of the workshop.

We took a process-oriented approach to curriculum evaluation as described by the Context-Input-Process-Product (CIPP) model.^24^ We collected formative feedback via surveys to guide revisions for future iterations of the curriculum (Appendix H). Participant feedback was obtained by asking open-ended questions: “What did you like most about this workshop?” and “What would you change about this workshop?” Acceptability of the curriculum was assessed by asking questions such as, “Would you recommend this workshop to a colleague?” Facilitators debriefed at the conclusion of each iteration, determining what went well and what needed to change. Several changes were made between the first, second, and final implementation of the curriculum (Figure 2).

### Curriculum Evaluation

To evaluate the efficacy of the curriculum, we measured attainment of the educational objectives via pre/postcurriculum quantitative surveys. Attainment of educational objective 1 was assessed using two of the three true/false knowledge questions (Appendix H): “It is best practice to wait until all medical workup is complete prior to discussing a SSRD diagnosis with a patient's family,” and “Parent buy-in of the diagnosis improves outcomes for children with SSRDs.” A two-sample *t* test was performed to compare the pre- and postcurriculum correct response rates, with significant differences defined as a *p* value less than .05.

Attainment of educational objective 2 was assessed using learners’ self-reported ratings of confidence in delivering an SSRD diagnosis, a Kirkpatrick Level 2 outcome on the Four Levels of Training Evaluation.^25^ A 5-point Likert scale was used, with response ratings ranging from 1 = *strongly disagree* to 5 = *strongly agree* in response to the statement: “I feel confident in my ability to deliver a new SSRD diagnosis to a child's parent” (Appendix H).

This study was determined to be exempt from continued review by the University of Washington Institutional Review Board (reference study No. 00020086).

## Results

The first iteration of the curriculum was given at a pediatric resident didactic session to 34 residents. Thirty-two surveys were obtained from these participants (94% response), and 66% reported having never received formal education on SSRD diagnostic communication. The second iteration of the curriculum was given on two separate occasions: at a national pediatric conference to 40 participants, 30 of whom filled out surveys (75% response), and at a resident didactic (at a different institution from the first iteration) to 23 pediatric residents, 100% of whom filled out surveys. Of the 30 participants at the national conference who responded, 14 were pediatric hospital medicine attendings (47%), four were pediatric residents (13%), and 12 were pediatric hospital medicine fellows (40%). For this second iteration, when combining both occasions, 53 total surveys (88% of participants) were collected, from 27 pediatric residents (51%), 12 pediatric hospital medicine fellows (23%), and 14 pediatric hospitalist attendings (26%). A total of 62% of these participants reported having never received formal education on SSRD diagnostic communication. Between the two iterations of the curriculum, there were 97 total participants, of whom 85 participated in the surveys (88% response).

### Participant Outcomes

Before the curriculum, a total of 32% of participants reported feeling confident in delivering an SSRD diagnosis. After participation in the curriculum, the confidence levels increased to 86% of learners. Before participation, 16% *strongly agreed* they felt confident in delivering an SSRD diagnosis, while 40% of participants *disagreed* or *strongly disagreed* that they felt confident, whereas none of the survey respondents indicated a lack of confidence after the curriculum.

Of the 85 total participants, 99% answered the first true/false knowledge question correctly in the postcurriculum survey, compared to 81% answering correctly in the precurriculum survey, demonstrating a statistically significant increase in knowledge following participation (*p* < .001). In response to the second true/false knowledge question, we observed a statistically significant increase in participants reporting the correct answer, from 80% precurriculum to 95% postcurriculum (*p* = .001), in response to the question: “When talking about a SSRD diagnosis, it is important to reassure parents by emphasizing that nothing is wrong with their child.” Before participating in the curriculum, 93% of participants answered the third true/false knowledge question correctly, compared to 100% of participants on the postcurriculum survey (*p* = .50).

**Figure 3.**
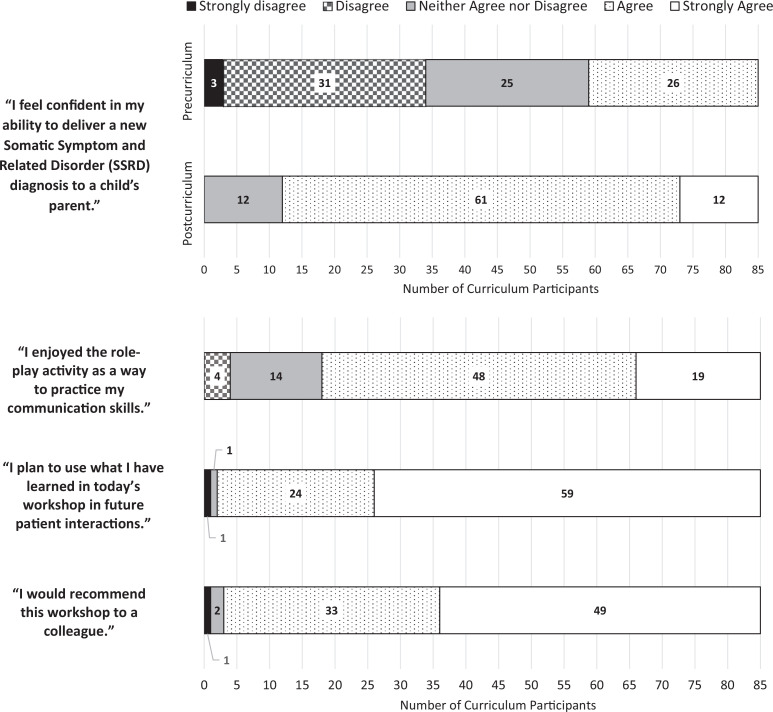
Curriculum participant survey responses; combined all iterations of the workshop.

Among all participants responding to the survey, 95% would recommend this curriculum to a colleague, of whom 60% *strongly agreed*. A total of 98% of participants *agreed* they would use these skills in their future clinical practice, of whom 71% *strongly agreed*. A total of 79% of participants *agreed* or *strongly agreed* that they enjoyed role-play to practice their skills (Figure 3).

## Discussion

Participants demonstrated reasonable achievement of the two educational objectives. Our most impactful observed change was in learner confidence levels in delivering an SSRD diagnosis. There was a 54% increase in participants who *strongly agreed* or *agreed* that they felt confident after participation in the curriculum, with none reporting that they were not confident in delivering an SSRD diagnosis after the curriculum. Additionally, there was a statistically significant increase of 15% of participants who correctly answered *false* to the statement: “When talking about a SSRD diagnosis, it is important to reassure parents by emphasizing that nothing is wrong with their child.” This indicates that, in addition to feeling increased confidence, participants demonstrated at least one measure of objective knowledge accuracy in diagnostic conversations to support this self-perception.

Our findings showed that participants recognized the importance of having diagnostic conversations for SSRDs early, even when there is still differential uncertainty. Although knowledge question three was also aimed at measuring attainment of the first educational objective, and the correct response rate postcurriculum was 100%, it did not show a significant difference in comparison to the correct response rate precurriculum. The lack of a statistically significant difference can be attributed to the very high correct response rate in the precurriculum survey, a rate of 93%, and in retrospect, the question was likely too easy for an astute test-taker. We cannot rely on this knowledge question to judge whether this fact was truly learned in relation to the curriculum itself.

Challenges for curriculum implementation included time as a limiting factor throughout all iterations. This is a topic in which our participants had little to no formal training, which sparked enthusiasm and multiple participant questions. We initially designed a 60-minute workshop because this amount of time is most realistic for pediatric residents in their busy clinical schedules. However, after the first iteration of the workshop, only 66% of participants reported having enough time to practice their skills. Therefore, we increased the curriculum to 90 minutes and shortened the didactic portion by incorporating script examples in the facilitator large-group demonstration. After making these changes, we saw an increase to 92% of participants who reported enough time to practice their skills. Even so, the educational material could easily be lengthened or expanded to accommodate an audience's needs. Participants had several follow-up questions throughout the didactic portion, pertaining to diagnostic criteria and inpatient management. In the future, we would like to address these knowledge and skills gaps via online modules or additional workshops.

We had limited time to address the ethical considerations participants raised. For example, some worried that abdominal pain in teenage girls would be misdiagnosed as DGBI without fully assessing for conditions such as endometriosis, and some recalled the rare cases in which SSRD diagnoses were later revealed to be another condition. In summary, we addressed these concerns by reiterating that in the world of medicine, early diagnostic closure is possible for any diagnosis (even common pediatric respiratory illnesses like asthma or bronchiolitis), and that many illnesses declare themselves over time. We reemphasized that the “M” in INFORM includes involving parents as a main management principle, and that parents should be encouraged to bring up new concerns. Still, these comments from the audience reflect both the ongoing moral distress clinicians face in making this diagnosis and the need for more research and diagnostic clarity in this area. Additionally, we suspect some of the moral distress we observed from participants may be alleviated by obtaining input from real recipients of these diagnostic deliveries. It would be helpful to obtain feedback from patients with SSRDs and their caregivers on the INFORM framework and to relay these experiences and comments to our participants.

Though we took many steps to set up role-play in a way that was more inviting to participants, we found ourselves having to prompt some quieter groups of learners. To help enhance participants’ comfort in the role-play activity, we added a facilitator-led large-group demonstration of an SSRD (Appendix C), and survey comments described how helpful it was to see the INFORM framework in action before practicing it themselves. Additionally, we added questions for the observer role to provide more structure during role-play (Appendix E) and a large-group debrief after the first case, for participants to take a break from acting and share common challenges. We also found that it is most helpful to have one facilitator per small group to help the flow of feedback. Still, this curriculum would benefit from incorporating trained standardized patients to play the caregiver and increase fidelity. This might also allow for more controlled variation in how caregivers respond to the clinician delivering the diagnosis (eg, pleased versus frustrated), allowing the standardized patient to guide the conversation in a certain manner or challenge the clinician.

Finally, pediatric residents were chosen as our target audience in the initial development of the curriculum. However, over the course of the year in which we developed the curriculum, we received a lot of interest from other educators, which is why our participant data includes fellows and attendings. We have also begun delivering the curriculum to nurse practitioners, psychiatry residents, and physical and occupational therapists at our institution who expressed interest in gaining these communication skills. We suspect that this curriculum may also be applicable to medical students, primary care providers, and even pediatric subspecialists, including gastroenterology, rheumatology, neurology, and respiratory specialists, as SSRDs span a variety of different symptoms.

We created a novel SSRD diagnostic communication curriculum via an iterative process. Several changes were made based on participant and facilitator feedback to create an engaging final product that participants would find valuable to their practice. The INFORM acronym was enthusiastically received by participants, many of whom commented on the practicality of having a framework for these difficult conversations, as well as scripts and analogies. We hope this framework prevents clinicians and caregivers from dismissing SSRDs as a last resort diagnosis of exclusion and allows patients to feel validated, to know that this illness is not “in their head.” With increased confidence levels in clinicians, we also hope that we are closer to improving parent buy-in of the diagnosis, thereby improving patient outcomes.

## Appendices


Curriculum Agenda.docxSlide Deck With Script.pptxScript for Case Demonstration by Facilitators.docxCases for Role-Play.docxObserver and Caregiver Guide for Role-Play.docxINFORM Quick Guide.docxGlossary of Acronyms.docxCurriculum Evaluation Forms.docx
*All appendices are peer reviewed as integral parts of the Original Publication.*
