# Supplementary material for: INFORM: A Pediatrician's Communication Curriculum About Diagnostic Conversations in Somatic Symptom and Related Disorders
Source: MedEdPORTAL. 2025 Dec 2;21:11561. doi: 10.15766/mep_2374-8265.11561 (PMC12669383; doi:10.15766/mep_2374-8265.11561)
Supplement: Supplementary file 1 — Curriculum Agenda.docxSlide Deck With Script.pptxScript for Case Demonstration by Facilitators.docxCases for Role-Play.docxObserver and Caregiver Guide for Role-Play.docxINFORM Quick Guide.docxGlossary of Acronyms.docxCurriculum Evaluation Forms.docx [file mep_2374-8265.11561-s001.zip › A. Curriculum Agenda.docx]

**INFORM SSRD Communication Curriculum: Agenda**

*For internal use by facilitators as general curriculum overview.*

**Learning Objectives:**

1. Recognize the importance of early diagnostic communication in the treatment of Somatic Symptom and Related Disorders (SSRD).
2. Develop greater confidence in delivering a SSRD diagnosis to a patient’s caregiver using the INFORM framework.

| Timing | Agenda Item | Activity Type |
| --- | --- | --- |
| - 1. min | 1. Facilitator introductions 2. Learning objectives | Large group didactic |
| 2-9 min | 1. Defining SSRDs 2. Biopsychosocial pathology of SSRDs: role of ACEs and attentional bias | Information delivery: large group didactic |
| 9-14 min | 1. Present patient case, Andy: functional abdominal pain 2. Role-playing demonstration (requires 2 facilitators): example of poor communication | Large group role-playing demonstration by facilitators |
| 14-18 min | Debrief of poor communication demo | Large group reflection |
| 18-20 min | Why is communication particularly important in cases of SSRD? Evidence in the literature | Information delivery: large group didactic |
| 20-23 min | Poll Question: What do you think is the best way for Andy’s parents to have heard about the diagnosis of functional abdominal pain? | Large group online poll and reflection |
| 23-37 min | Details of the INFORM framework   1. **I:** introduce the conversation. Being explicit about diagnostic discussions 2. **N:** narrate and name the illness. Being on the same page as the family, providing diagnostic clarity 3. **F**: feedback from family. Ensuring you are on the same page 4. **O**: orient to SSRDs. Describing the illness, using analogies, describing the role of the psyche 5. **R**: reframe. Moving toward functional recovery and away from further testing, focusing on positive prognostic outcomes 6. **M**: management principles. Providing broad overview of general management, emphasizing routine and “rewiring” pathways, and parental advocacy   Within each, providing example scripts | Information delivery: large group didactic |
| 37-43 min | 1. Role-playing demonstration (requuires two facilitators: Facilitators act out the same patient case (Andy) using INFORM framework 2. Debrief of scenario: what went well? What strategies were used? | Large group role-playing demonstration by facilitators + large group reflection |
| - 1. min | Set-up role-play exercise: provide instructions, split into groups of 3-4 (1 per table), orient to summary sheets | Large group didactic |
| 46-58 min | Role-play in small groups: Practice Case #1, abnormal movements | Small group exercise |
| - 1. min | Debrief after first patient case: What are some challenges? What went well? | Large group debrief |
| 63-68 min | Parents’ Frequently Asked Questions -Example scripts | Large group didactic and with audience feedback |
| 68-88 min | Role-play exercises, continued: Practice Case #2 (abdominal pain), Practice Case #3 (weakness, inability to walk) | Small group exercise |
| 88-90 min | Summary of take-home points | Large group didactic |
