# Supplementary material for: INFORM: A Pediatrician's Communication Curriculum About Diagnostic Conversations in Somatic Symptom and Related Disorders
Source: MedEdPORTAL. 2025 Dec 2;21:11561. doi: 10.15766/mep_2374-8265.11561 (PMC12669383; doi:10.15766/mep_2374-8265.11561)
Supplement: Supplementary file 1 — Curriculum Agenda.docxSlide Deck With Script.pptxScript for Case Demonstration by Facilitators.docxCases for Role-Play.docxObserver and Caregiver Guide for Role-Play.docxINFORM Quick Guide.docxGlossary of Acronyms.docxCurriculum Evaluation Forms.docx [file mep_2374-8265.11561-s001.zip › B. Slide Deck With Script.pptx]

## Slide 1
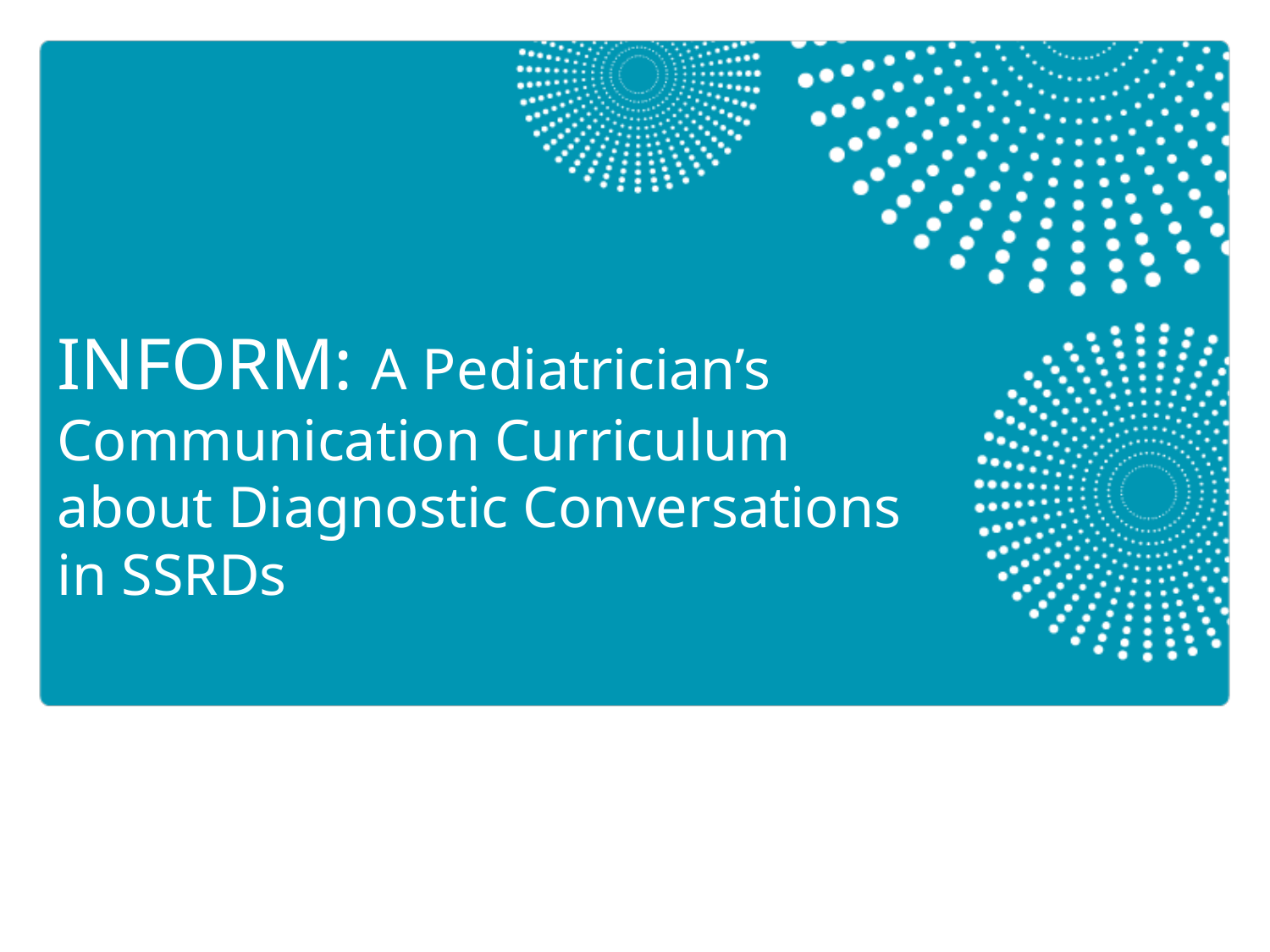

INFORM: A Pediatrician’s Communication Curriculum about Diagnostic Conversations in SSRDs

## Slide 2
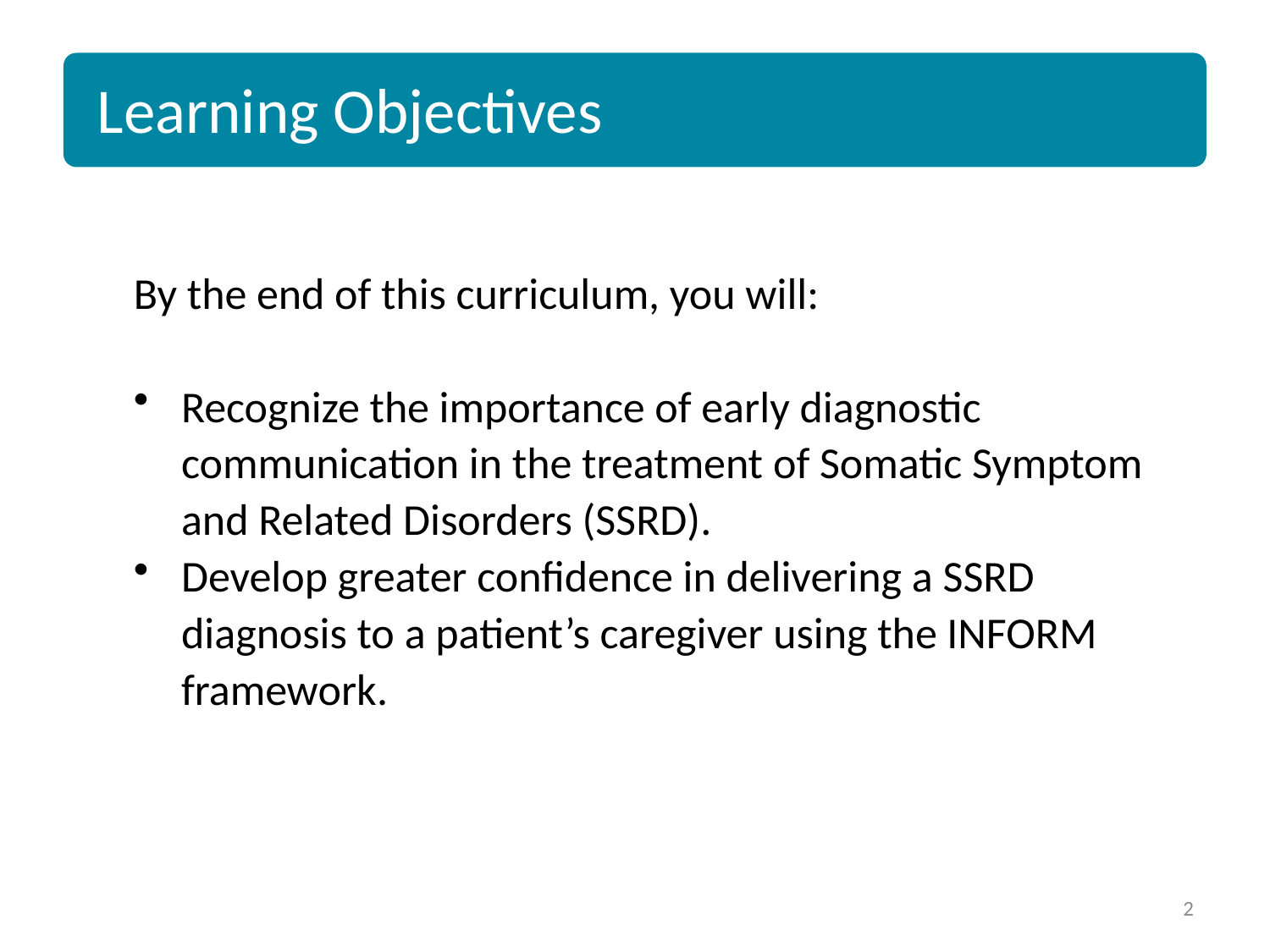

Learning Objectives
By the end of this curriculum, you will:
Recognize the importance of early diagnostic communication in the treatment of Somatic Symptom and Related Disorders (SSRD).
Develop greater confidence in delivering a SSRD diagnosis to a patient’s caregiver using the INFORM framework.
2

## Slide 3
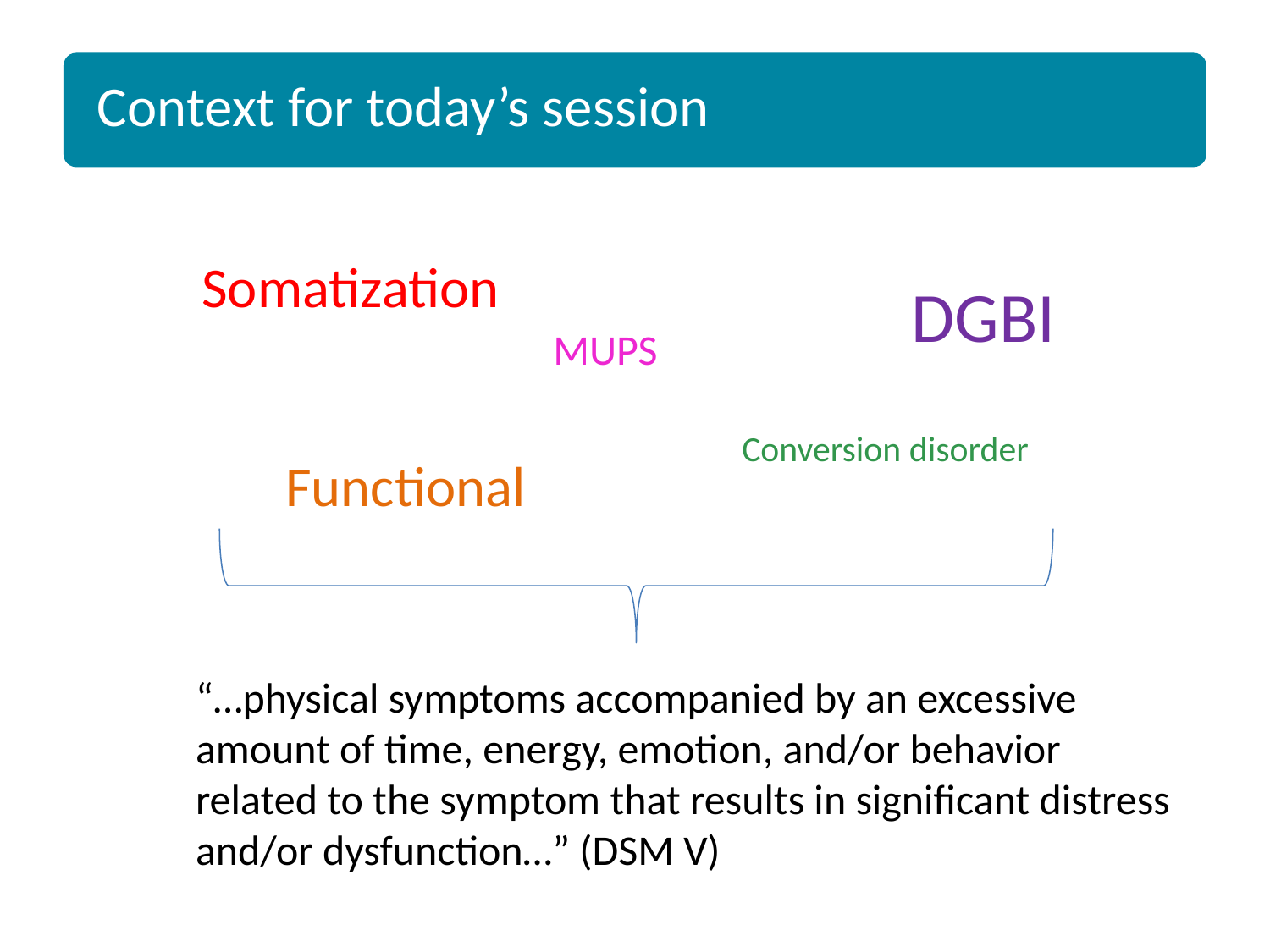

Context for today’s session
Somatization
DGBI
MUPS
Conversion disorder
Functional
“…physical symptoms accompanied by an excessive amount of time, energy, emotion, and/or behavior related to the symptom that results in significant distress and/or dysfunction…” (DSM V)

## Slide 4
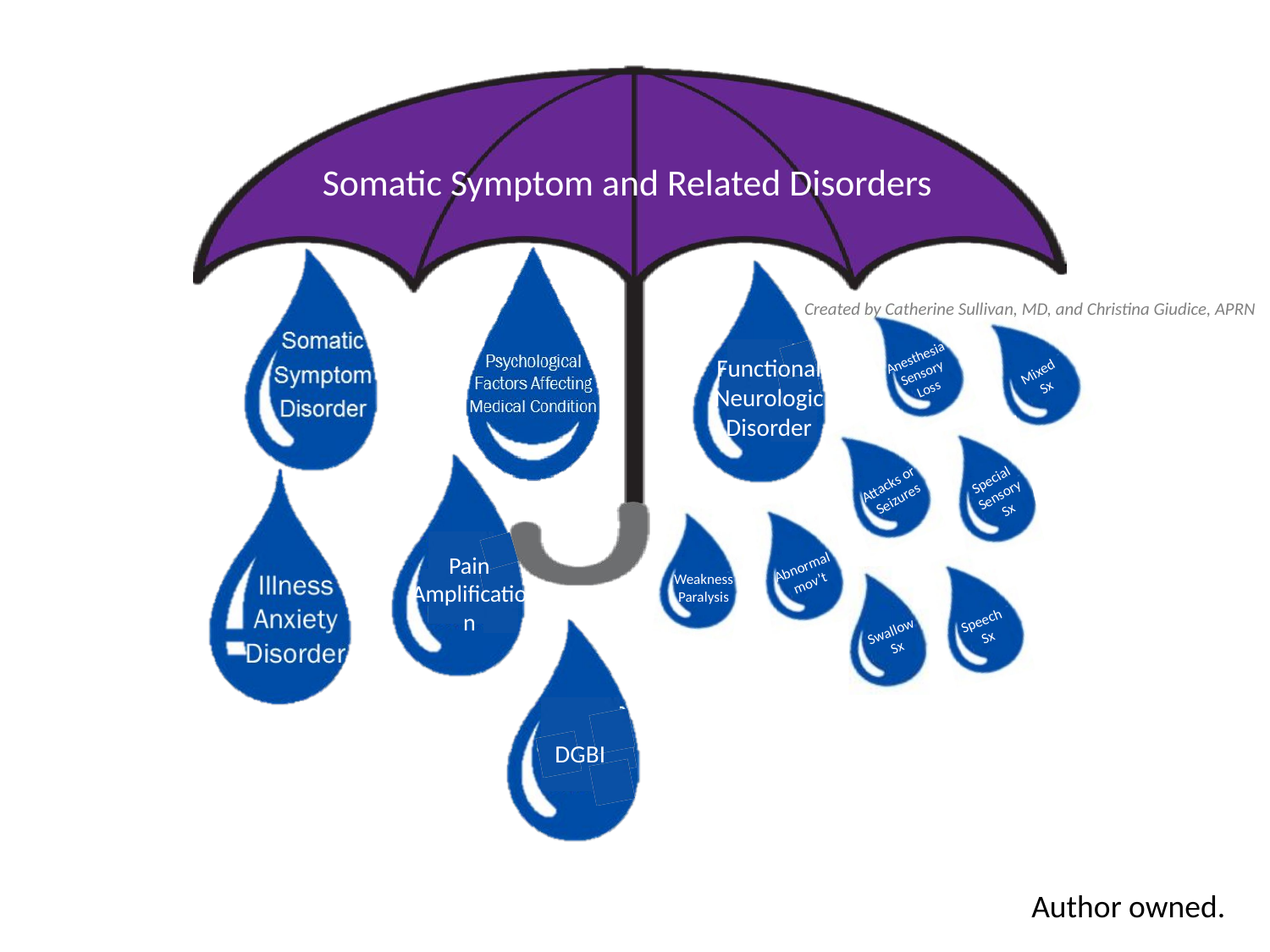

Context for today’s session
Somatic Symptom and Related Disorders
Created by Catherine Sullivan, MD, and Christina Giudice, APRN
Anesthesia
Sensory
Loss
Functional Neurologic Disorder
Mixed
Sx
Special
Sensory
Sx
Attacks or
Seizures
Pain Amplification
Abnormal
mov’t
Weakness
Paralysis
Speech
Sx
Swallow
Sx
DGBI
Author owned.

## Slide 5
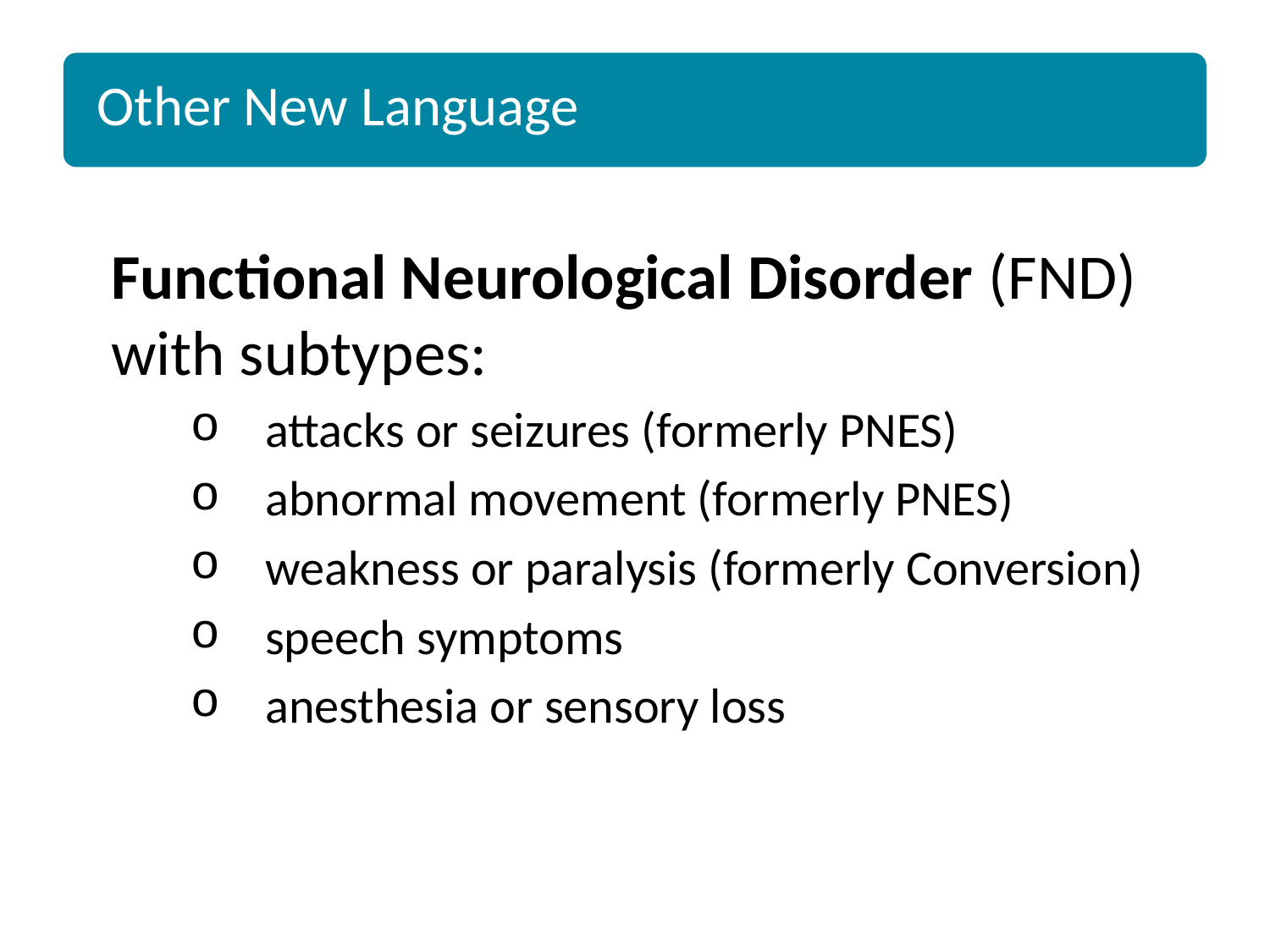

Other New Language
Functional Neurological Disorder (FND) with subtypes:
 attacks or seizures (formerly PNES)
 abnormal movement (formerly PNES)
 weakness or paralysis (formerly Conversion)
 speech symptoms
 anesthesia or sensory loss

## Slide 6
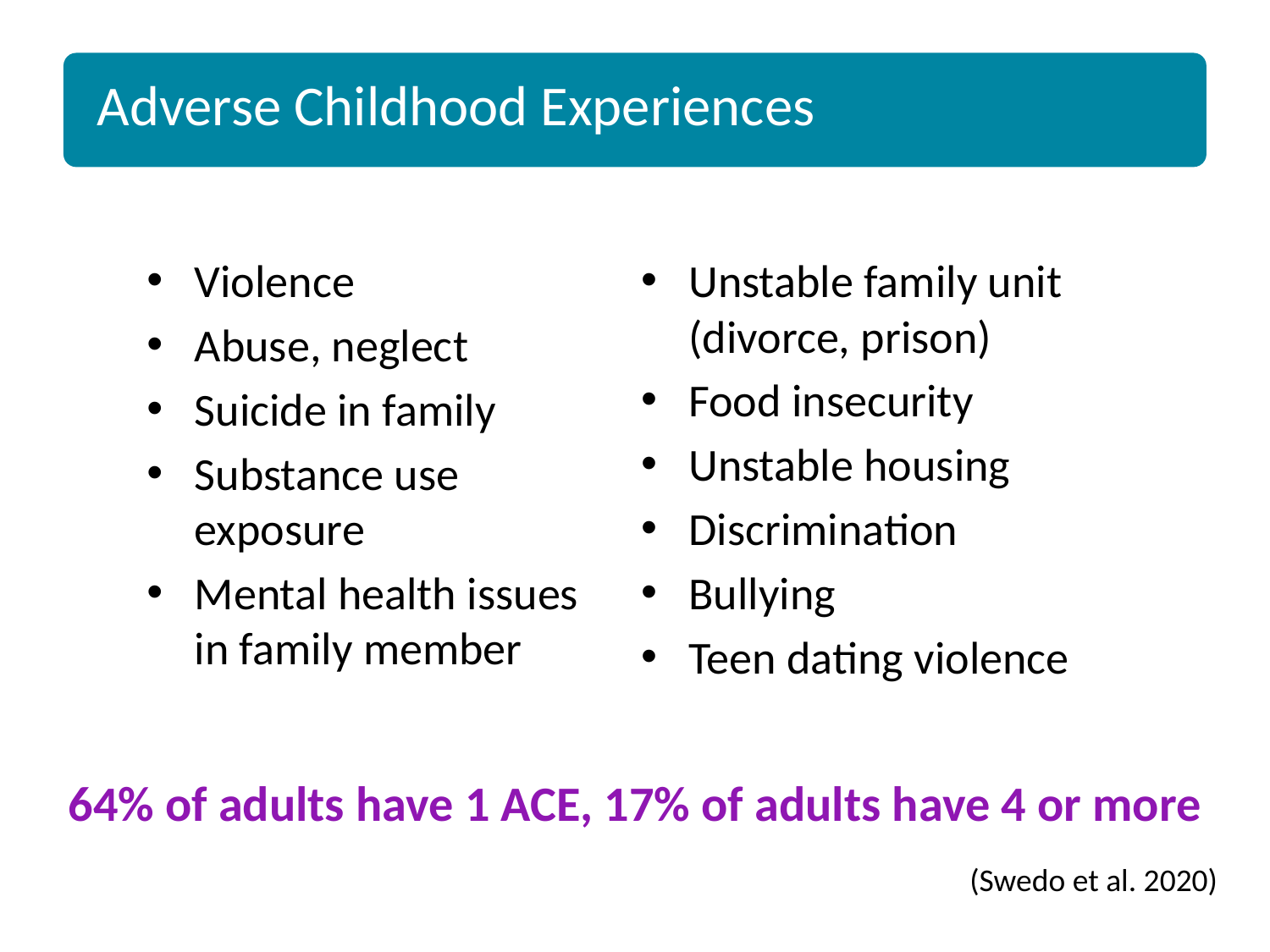

Adverse Childhood Experiences
Violence
Abuse, neglect
Suicide in family
Substance use exposure
Mental health issues in family member
Unstable family unit (divorce, prison)
Food insecurity
Unstable housing
Discrimination
Bullying
Teen dating violence
64% of adults have 1 ACE, 17% of adults have 4 or more
 (Swedo et al. 2020)

## Slide 7
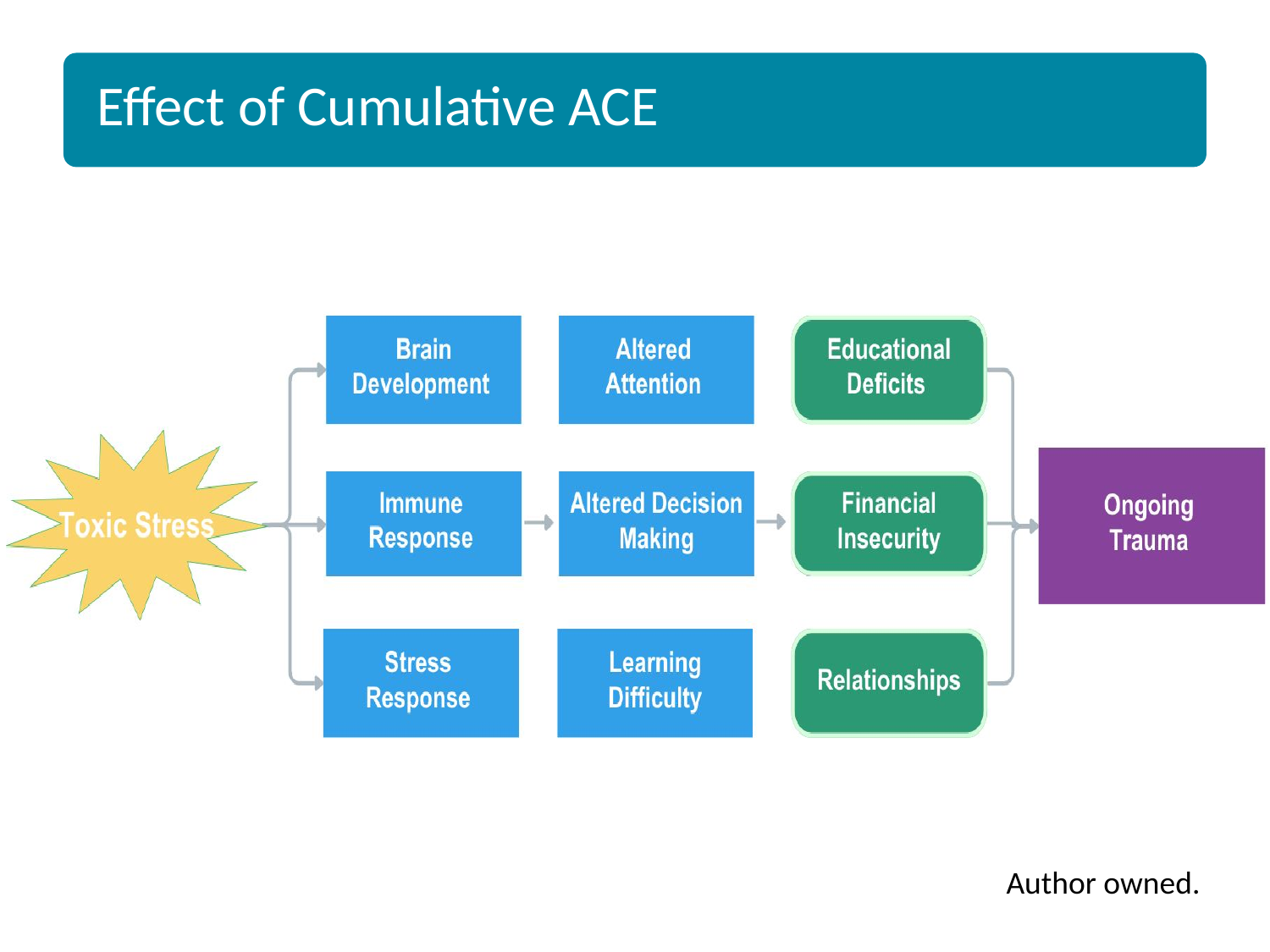

Effect of Cumulative ACE
Author owned.

## Slide 8
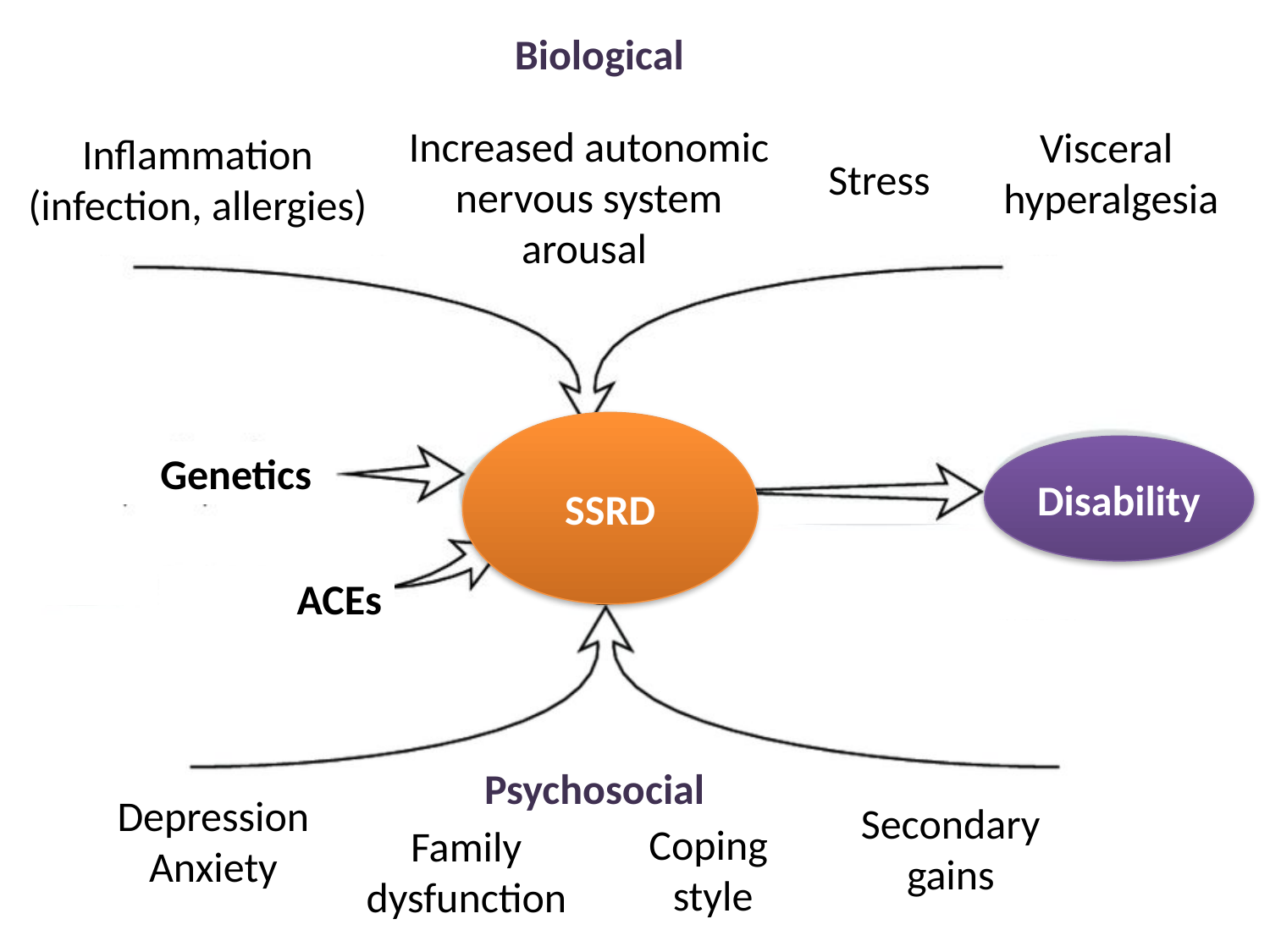

Biological
Increased autonomic nervous system arousal
Visceral
hyperalgesia
Inflammation (infection, allergies)
Stress
SSRD
Disability
Genetics
ACEs
Psychosocial
Depression
Anxiety
Secondary gains
Coping
style
Family
dysfunction

## Slide 9
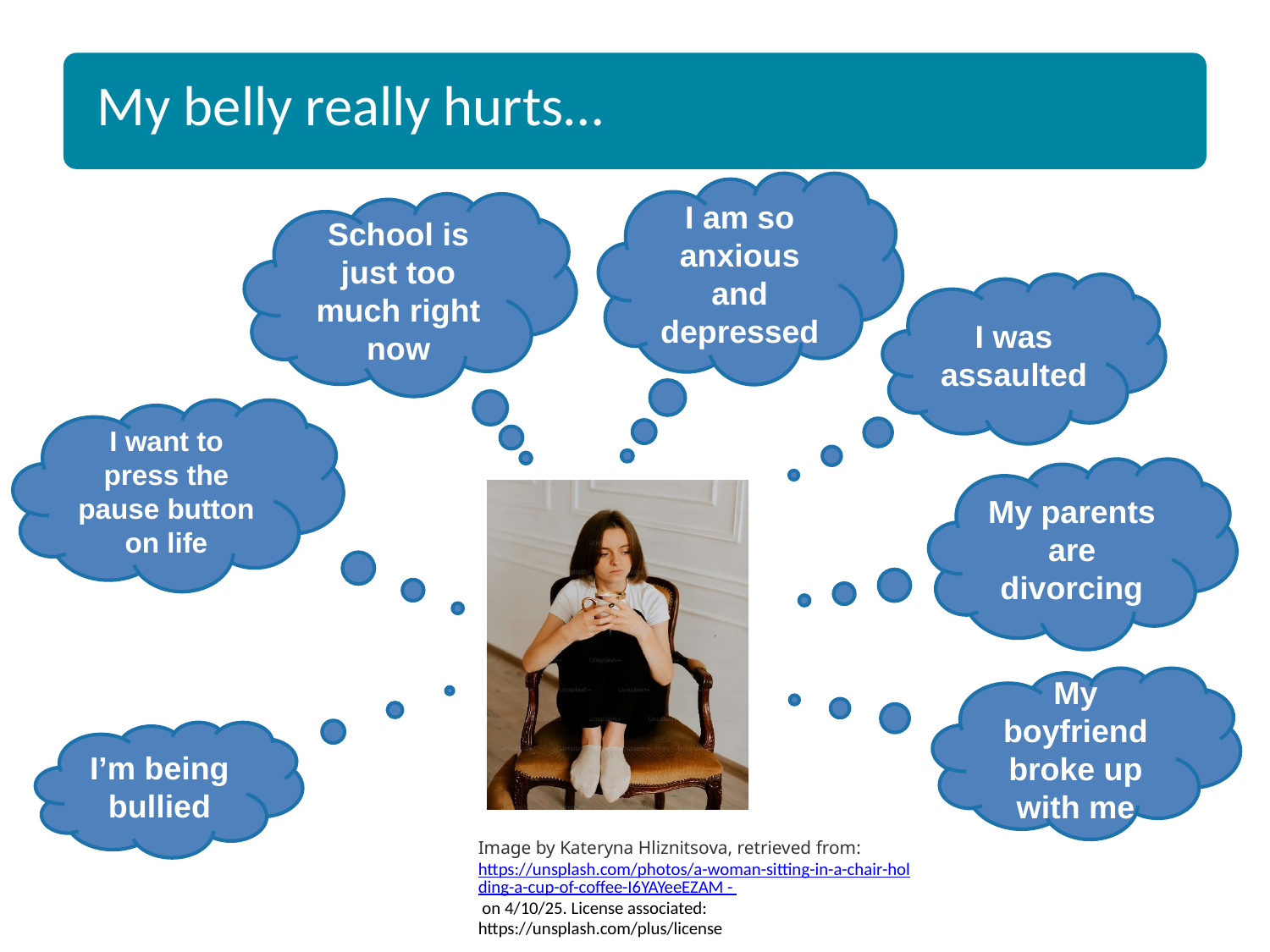

My belly really hurts…
I am so anxious and depressed
School is just too much right now
I was assaulted
I want to press the pause button on life
My parents are divorcing
My boyfriend broke up with me
I’m being bullied
Image by Kateryna Hliznitsova, retrieved from: https://unsplash.com/photos/a-woman-sitting-in-a-chair-holding-a-cup-of-coffee-I6YAYeeEZAM - on 4/10/25. License associated: https://unsplash.com/plus/license

## Slide 10
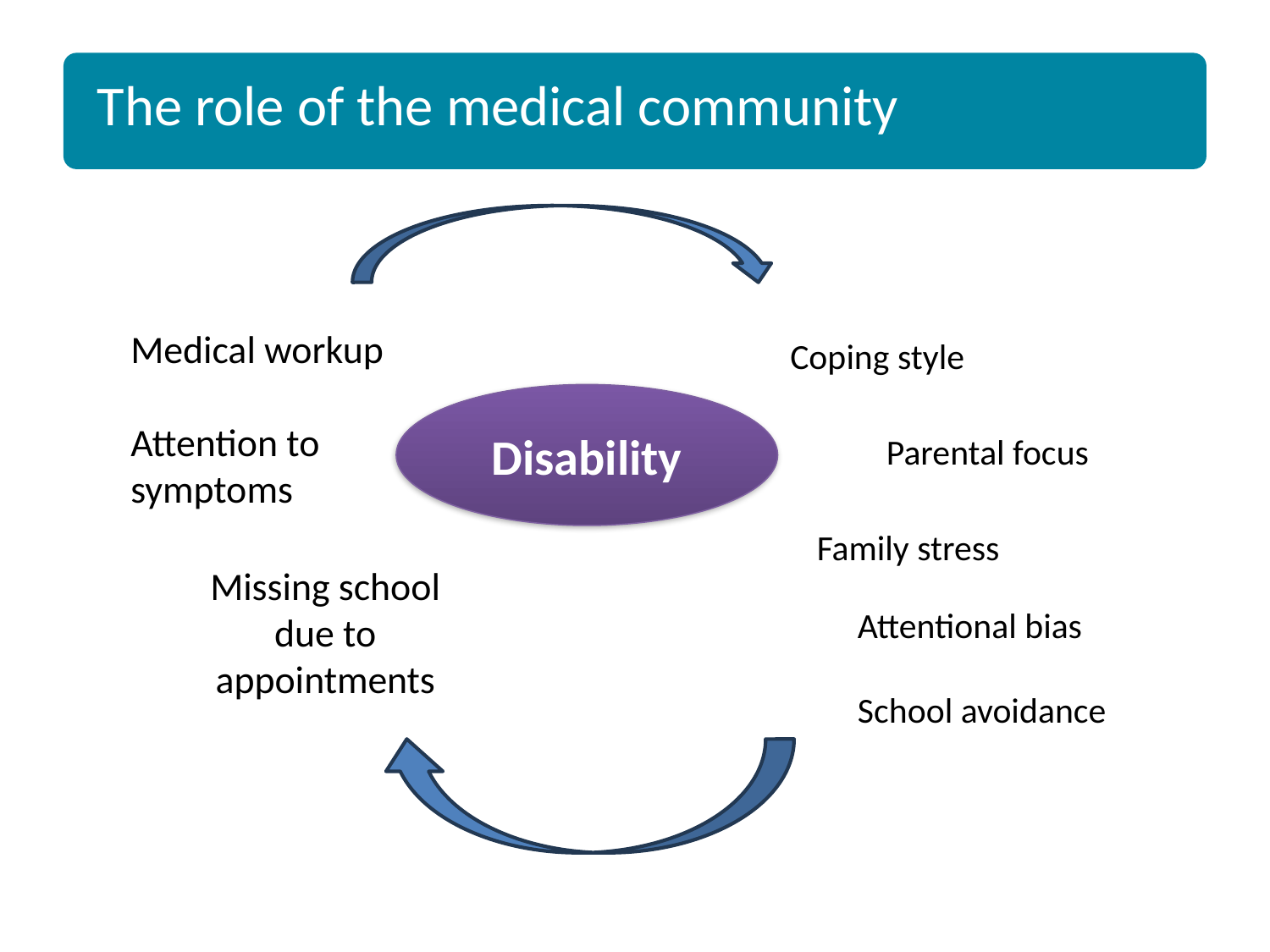

The role of the medical community
Medical workup
Attention to symptoms
Coping style
Disability
Parental focus
Family stress
Missing school due to appointments
Attentional bias
School avoidance

## Slide 11
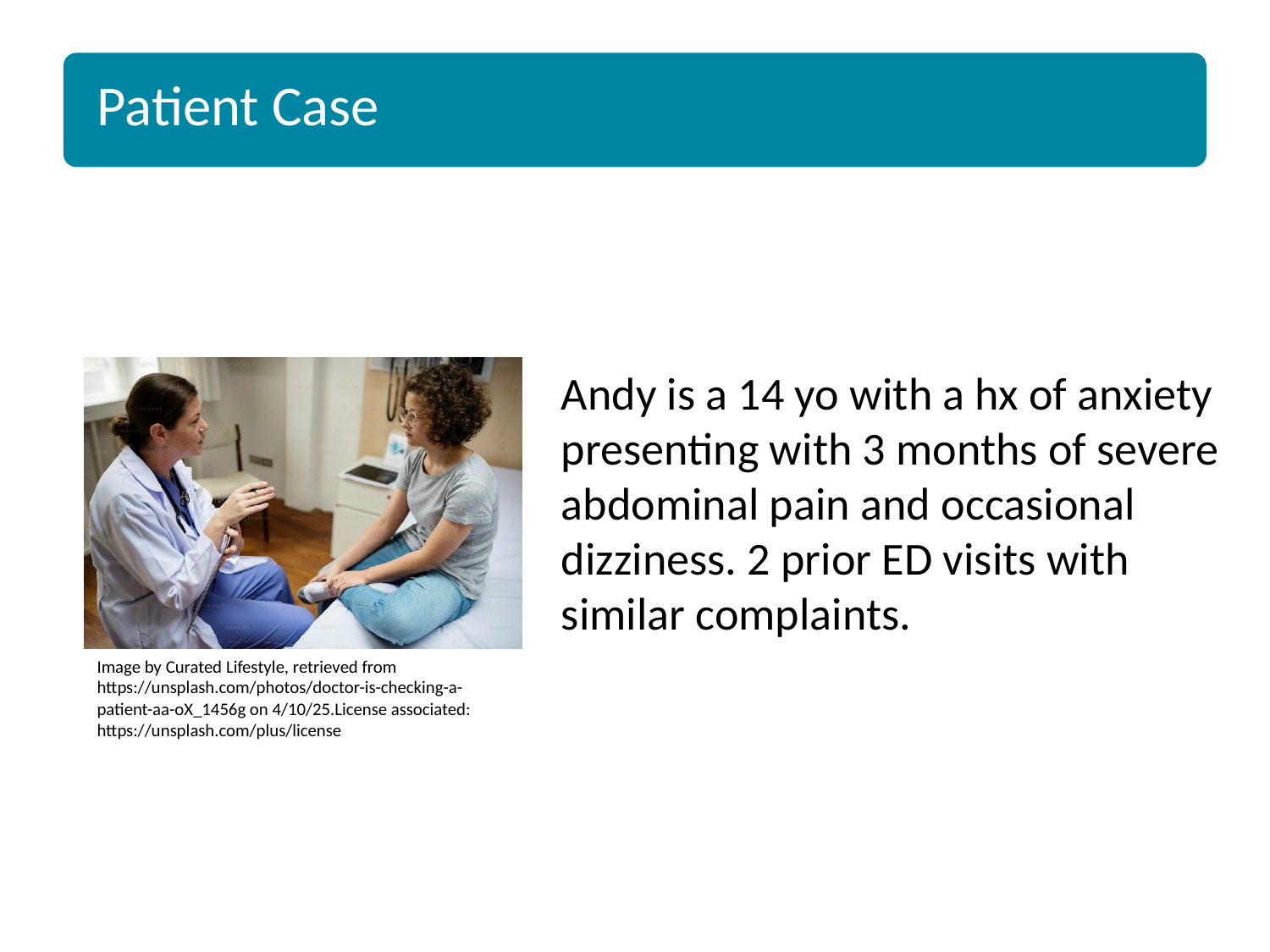

Patient Case
Andy is a 14 yo with a hx of anxiety presenting with 3 months of severe abdominal pain and occasional dizziness. 2 prior ED visits with similar complaints.
Image by Curated Lifestyle, retrieved from https://unsplash.com/photos/doctor-is-checking-a-patient-aa-oX_1456g on 4/10/25.License associated: https://unsplash.com/plus/license

## Slide 12
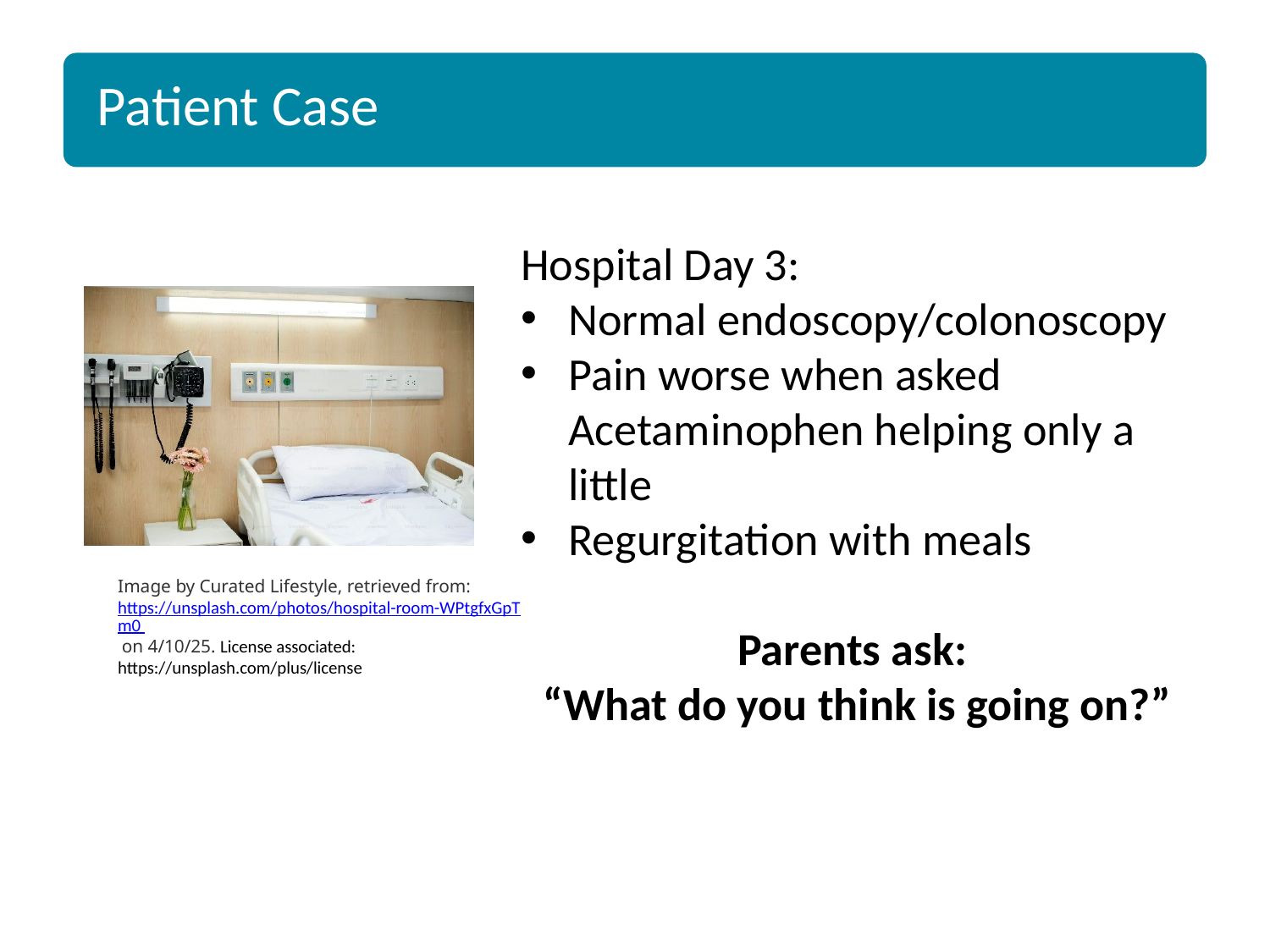

Patient Case
Hospital Day 3:
Normal endoscopy/colonoscopy
Pain worse when asked Acetaminophen helping only a little
Regurgitation with meals
Parents ask:
“What do you think is going on?”
Image by Curated Lifestyle, retrieved from: https://unsplash.com/photos/hospital-room-WPtgfxGpTm0 on 4/10/25. License associated: https://unsplash.com/plus/license

## Slide 13
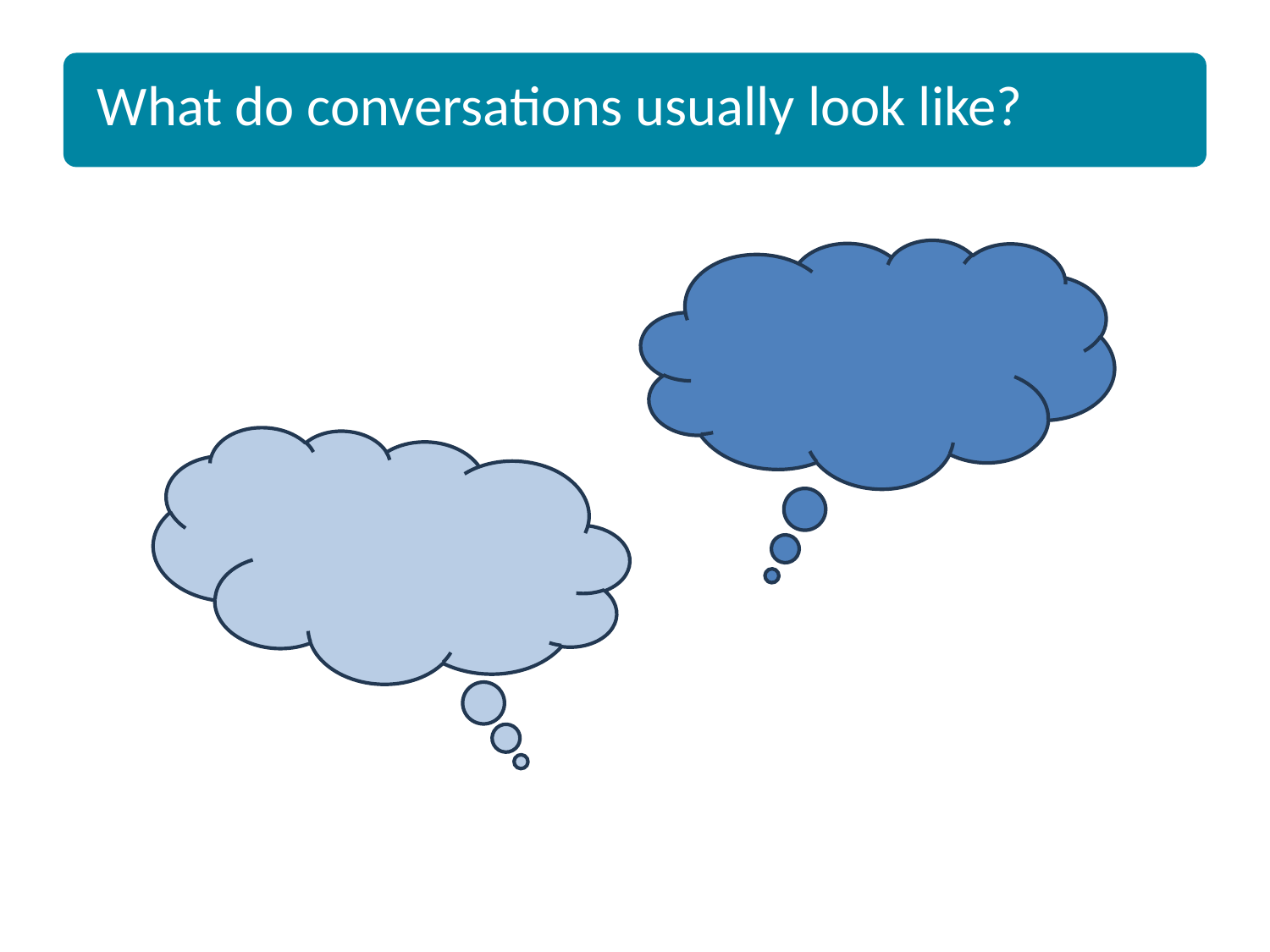

What do conversations usually look like?

## Slide 14
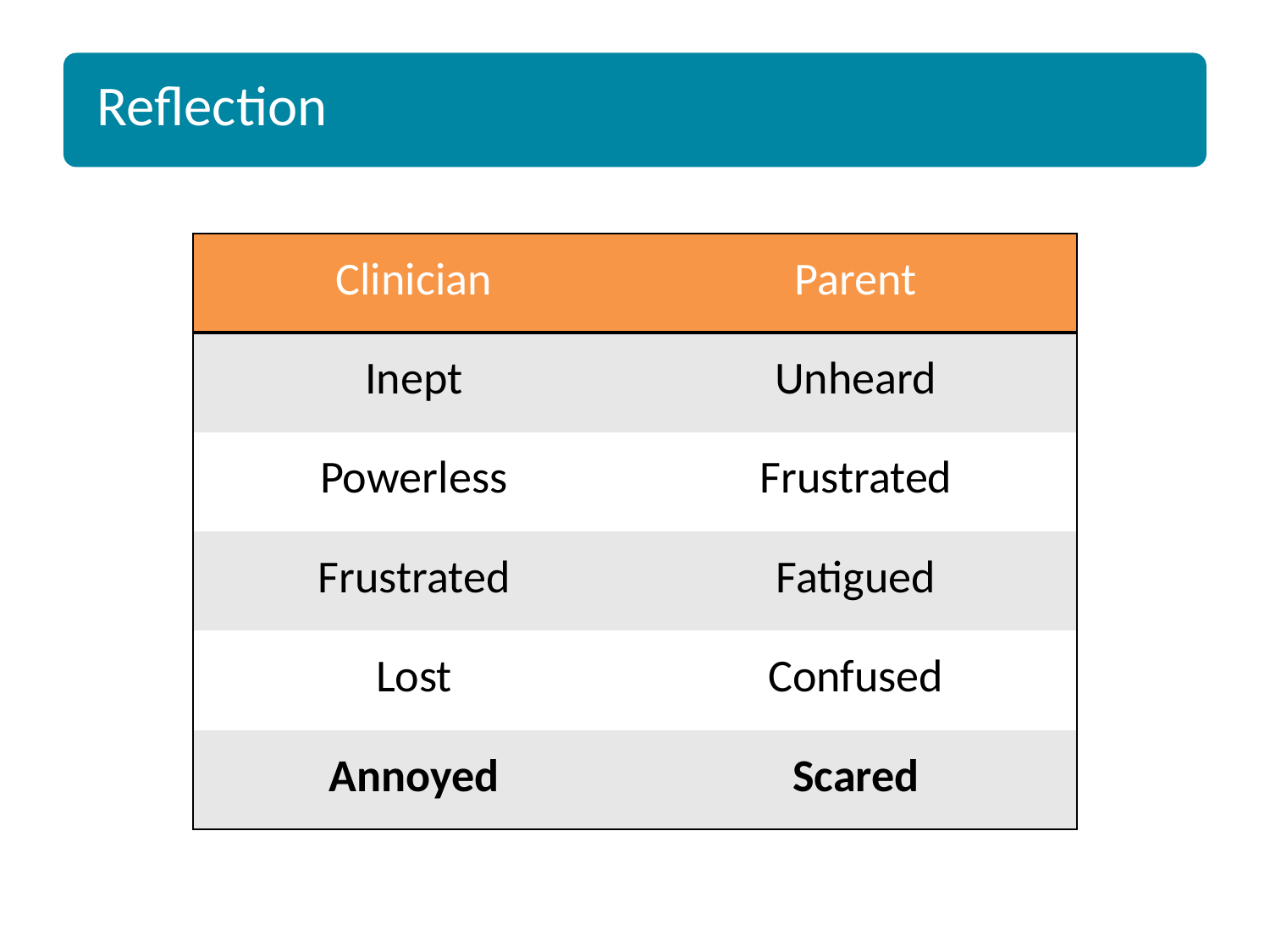

Reflection
| Clinician | Parent |
| --- | --- |
| Inept | Unheard |
| Powerless | Frustrated |
| Frustrated | Fatigued |
| Lost | Confused |
| Annoyed | Scared |

## Slide 15
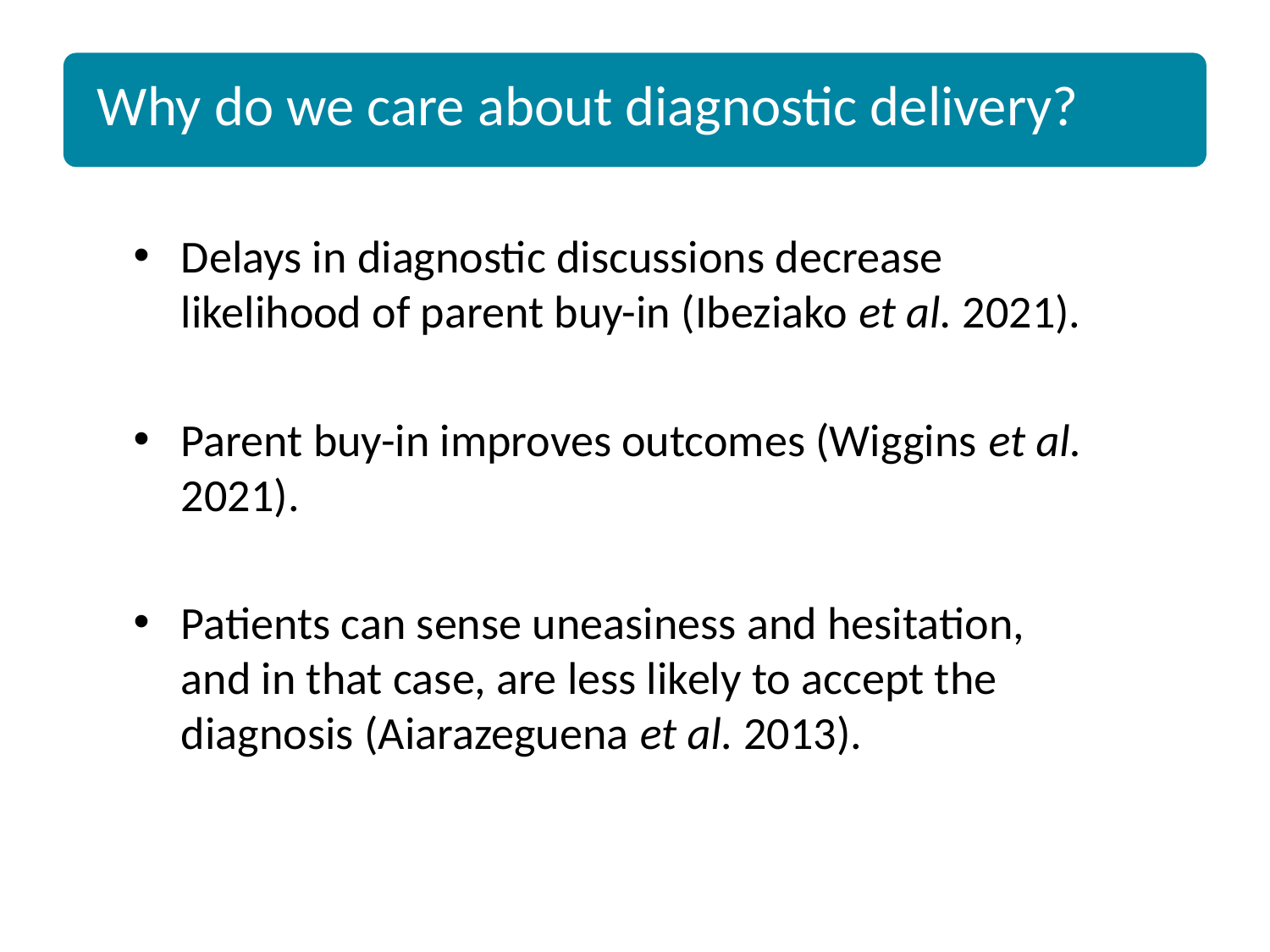

Why do we care about diagnostic delivery?
Delays in diagnostic discussions decrease likelihood of parent buy-in (Ibeziako et al. 2021).
Parent buy-in improves outcomes (Wiggins et al. 2021).
Patients can sense uneasiness and hesitation, and in that case, are less likely to accept the diagnosis (Aiarazeguena et al. 2013).

## Slide 16
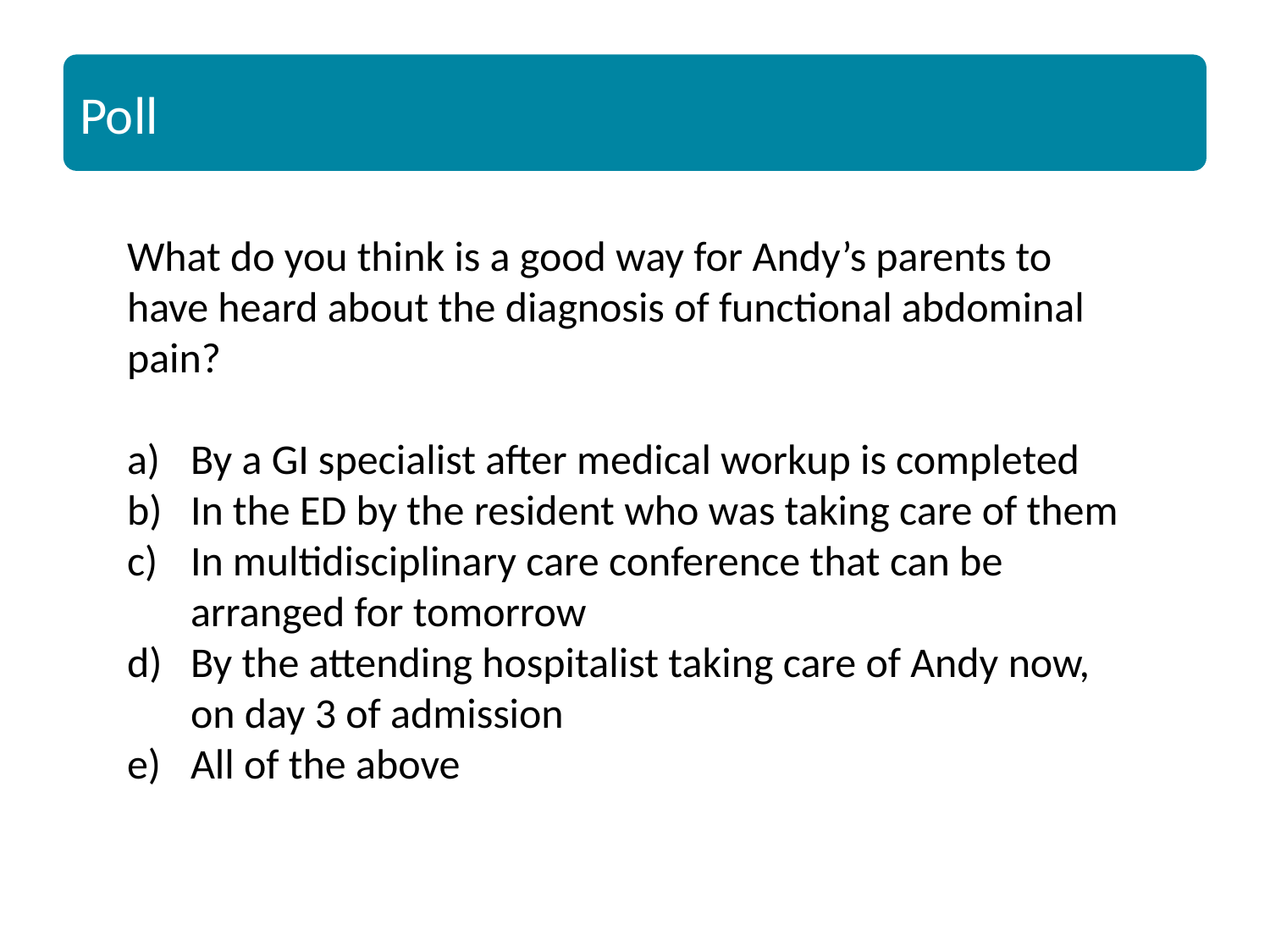

Poll
What do you think is a good way for Andy’s parents to have heard about the diagnosis of functional abdominal pain?
By a GI specialist after medical workup is completed
In the ED by the resident who was taking care of them
In multidisciplinary care conference that can be arranged for tomorrow
By the attending hospitalist taking care of Andy now, on day 3 of admission
All of the above

## Slide 17
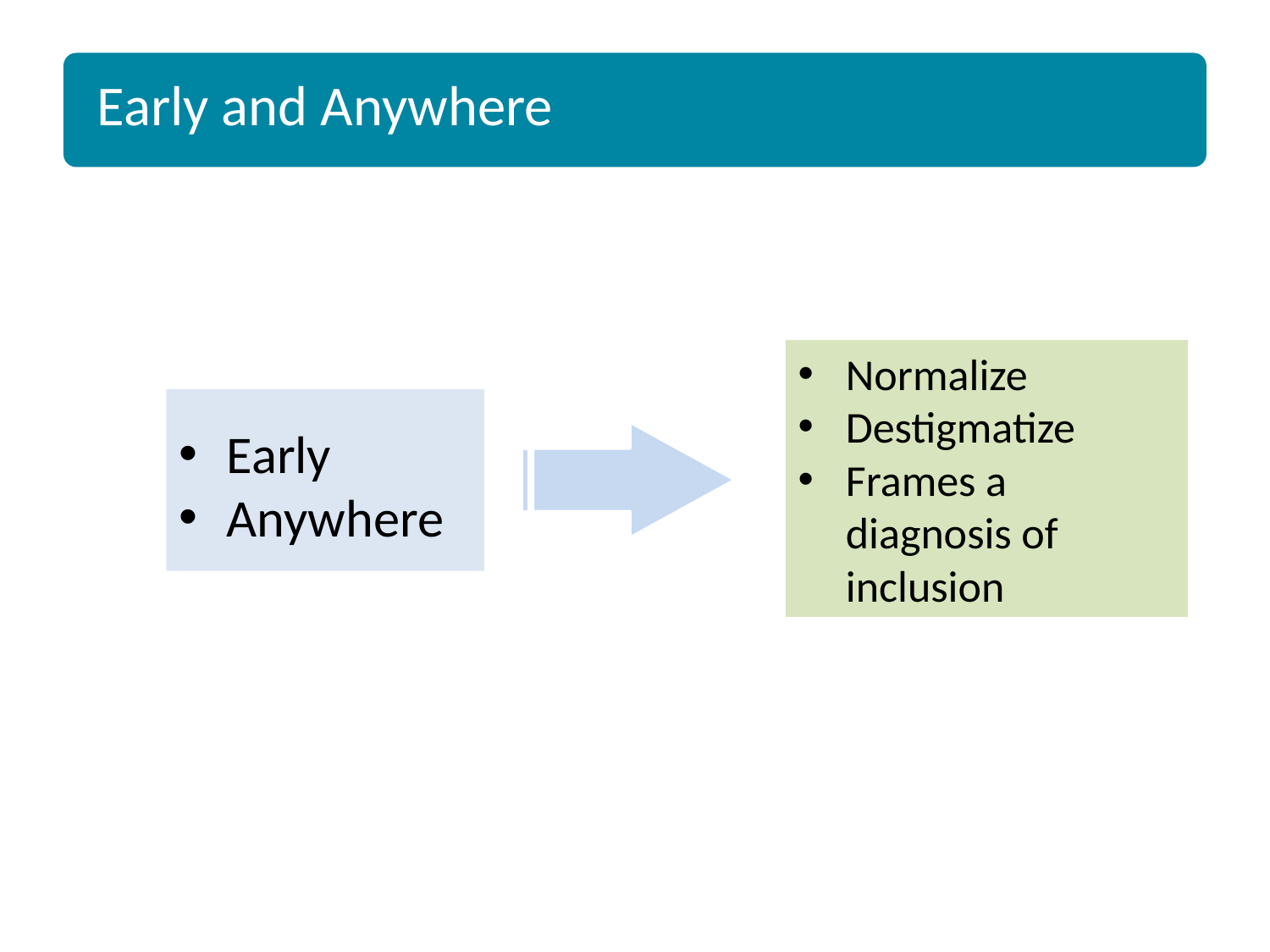

Early and Anywhere
Normalize
Destigmatize
Frames a diagnosis of inclusion
Early
Anywhere

## Slide 18
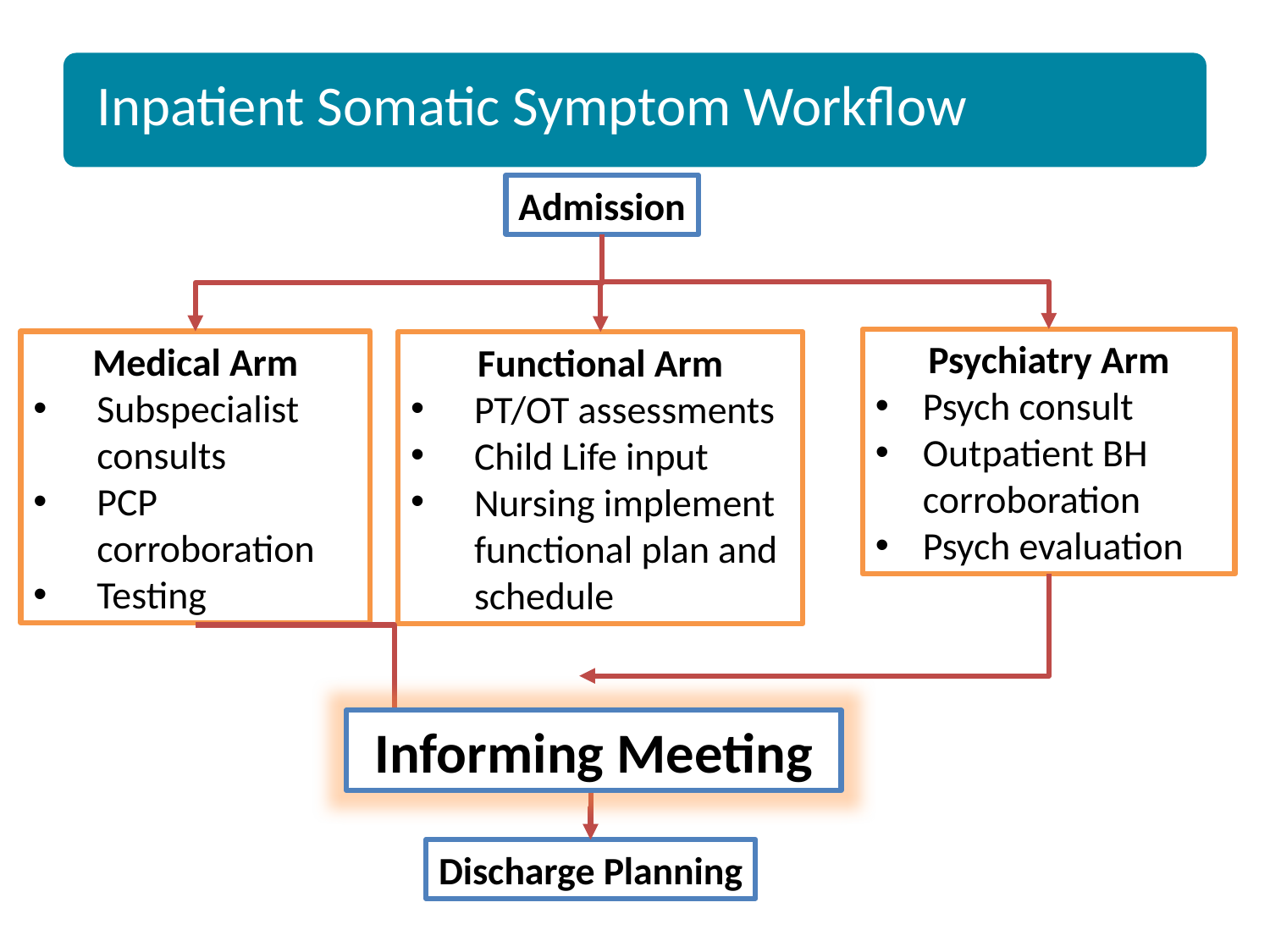

Inpatient Somatic Symptom Workflow
Admission
Psychiatry Arm
Psych consult
Outpatient BH corroboration
Psych evaluation
Medical Arm
Subspecialist consults
PCP corroboration
Testing
Functional Arm
PT/OT assessments
Child Life input
Nursing implement functional plan and schedule
Informing Meeting
Informing Meeting
Discharge Planning

## Slide 19
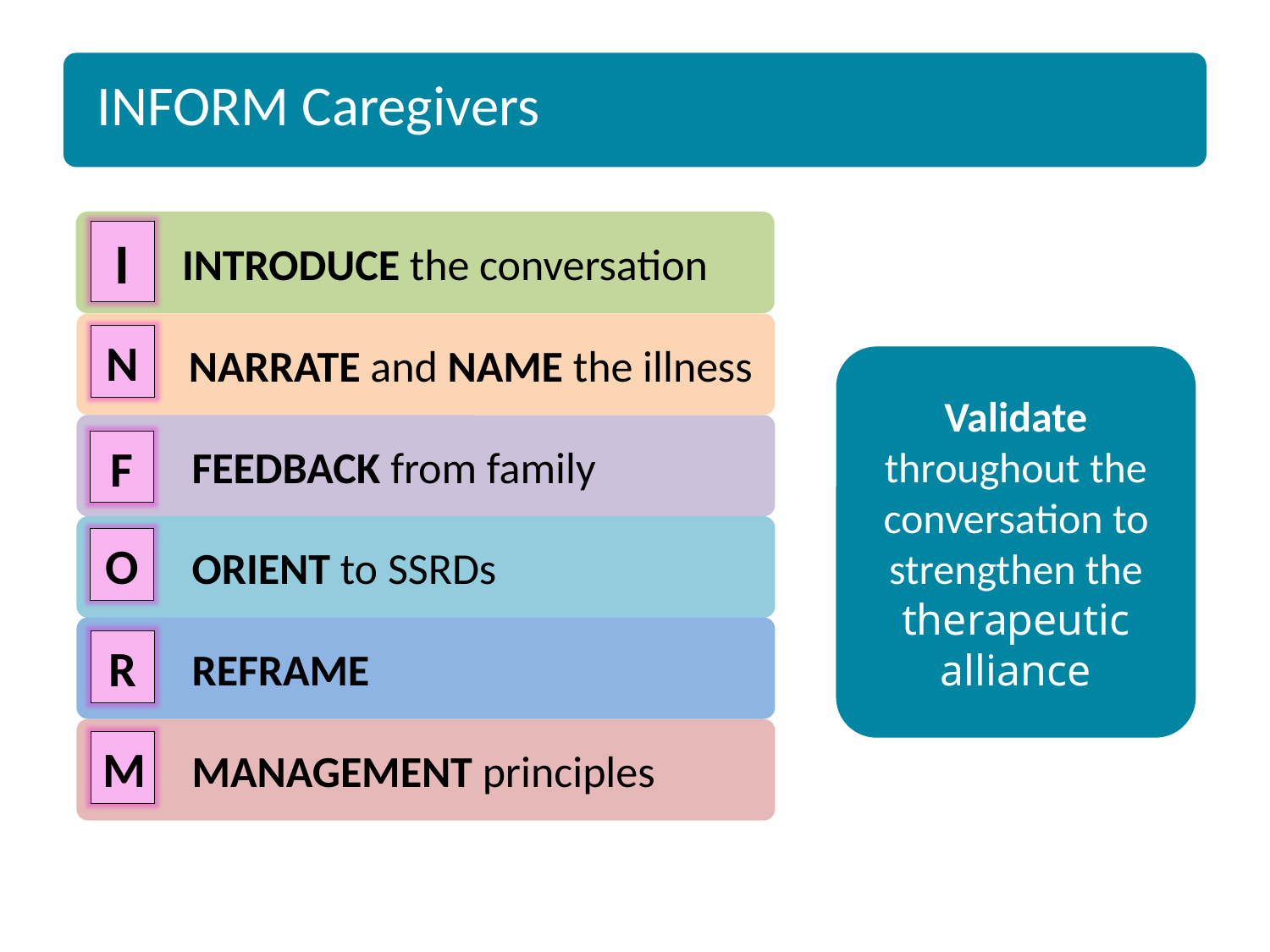

INFORM Caregivers
 INTRODUCE the conversation
I
 NARRATE and NAME the illness
N
Validate throughout the conversation to strengthen the therapeutic alliance
 FEEDBACK from family
F
 ORIENT to SSRDs
O
 REFRAME
R
 MANAGEMENT principles
M

## Slide 20
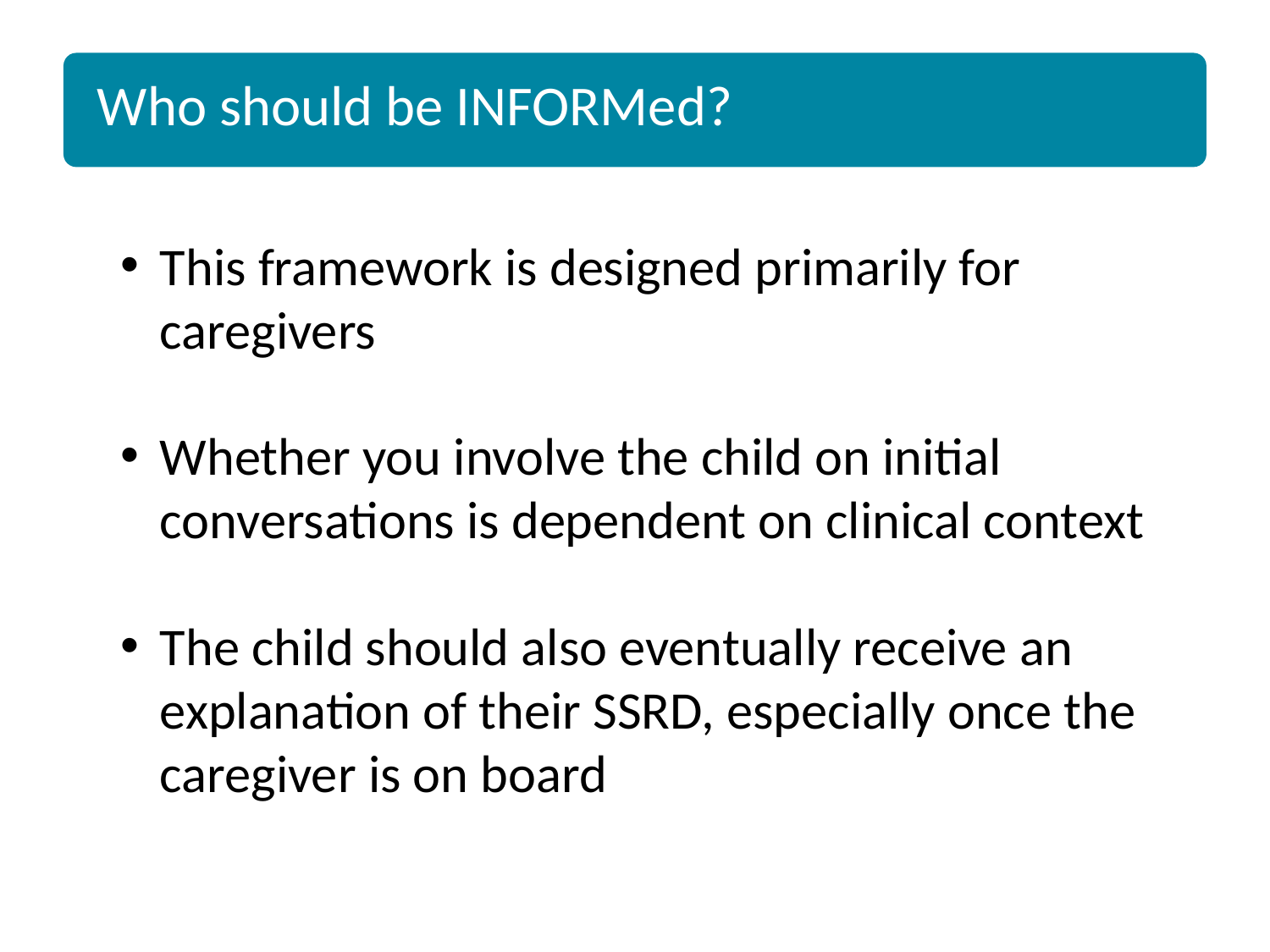

Who should be INFORMed?
This framework is designed primarily for caregivers
Whether you involve the child on initial conversations is dependent on clinical context
The child should also eventually receive an explanation of their SSRD, especially once the caregiver is on board

## Slide 21
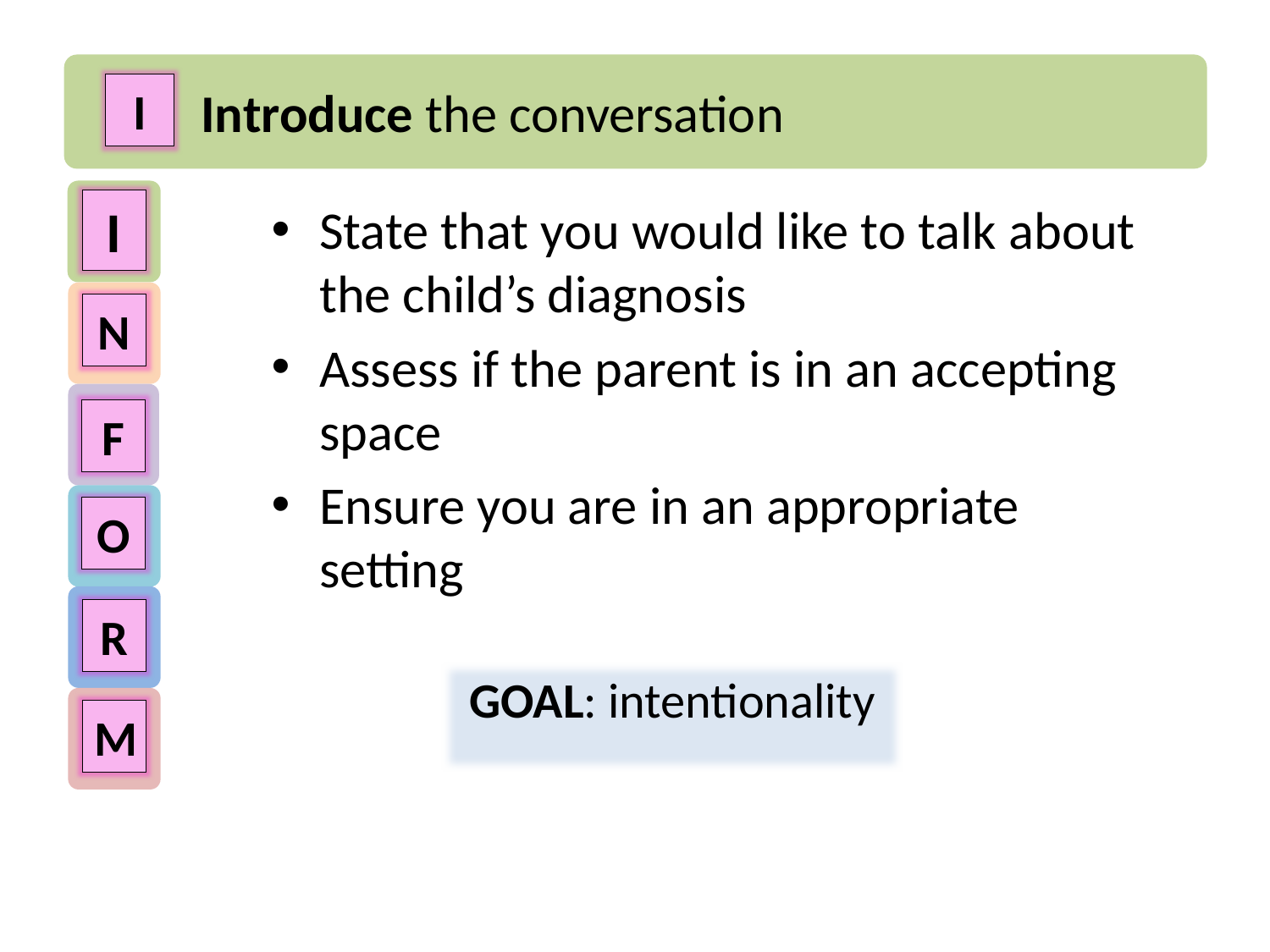

Introduce the conversation
Step 1: Introduce the conversation
I
I
State that you would like to talk about the child’s diagnosis
Assess if the parent is in an accepting space
Ensure you are in an appropriate setting
I
N
F
O
R
GOAL: intentionality
M

## Slide 22
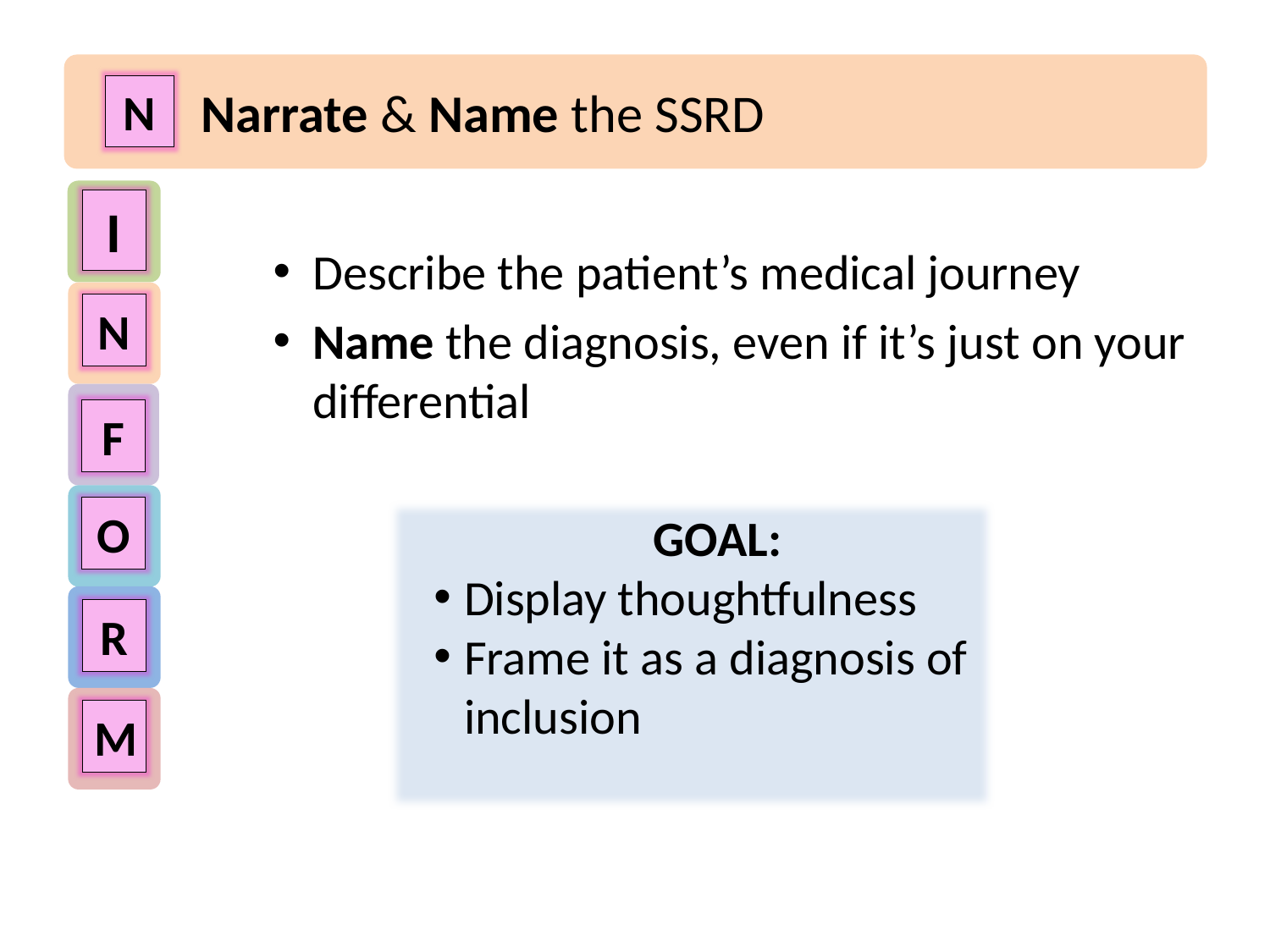

Narrate & Name the SSRD
Step 2: Narrate & Name the SSRD
Step 2: Narrate & Name the SSRD
N
I
Describe the patient’s medical journey
Name the diagnosis, even if it’s just on your differential
N
N
F
O
GOAL:
Display thoughtfulness
Frame it as a diagnosis of inclusion
R
M

## Slide 23
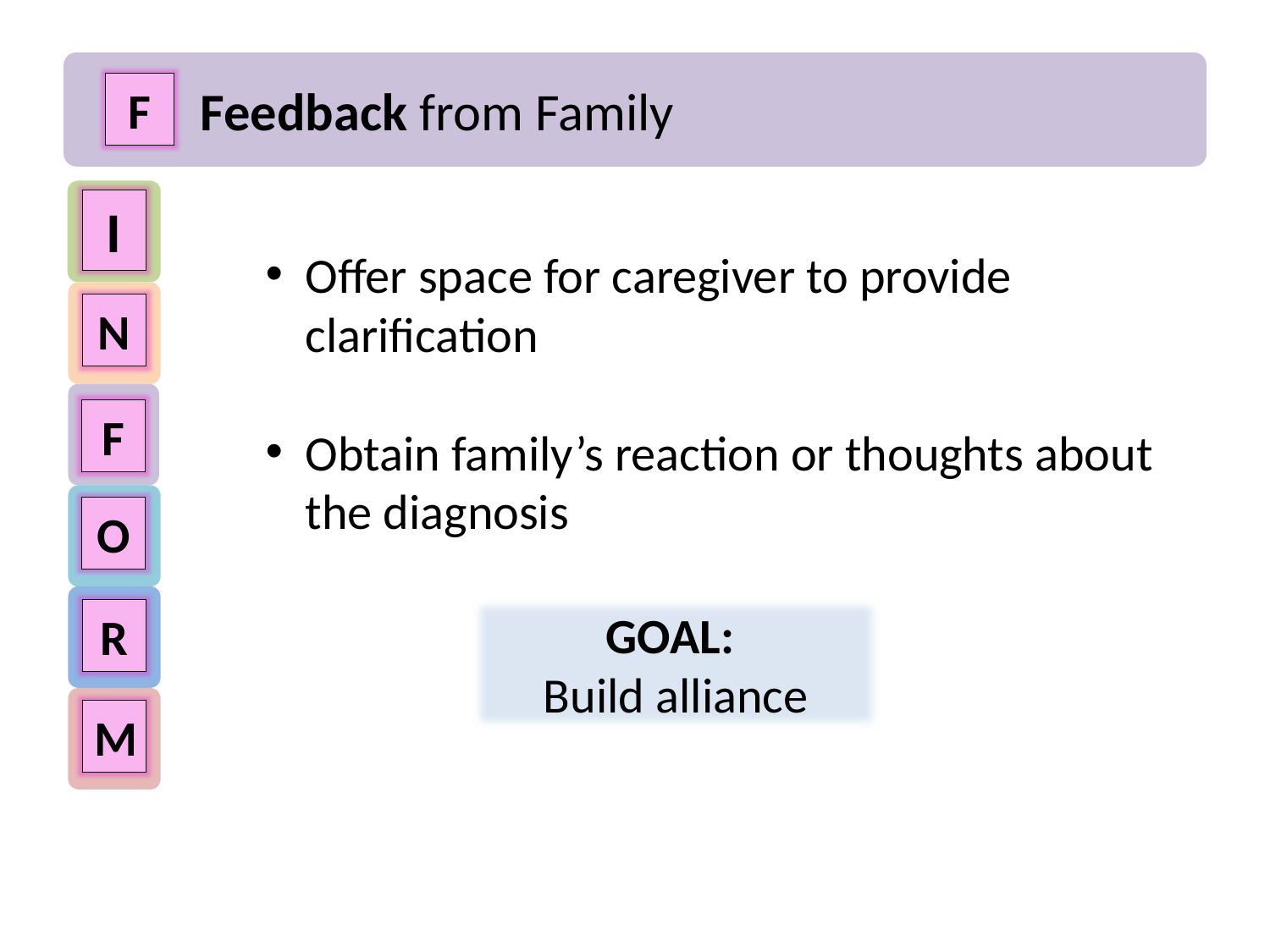

Feedback from Family
Step 3: Feedback from family
F
I
Offer space for caregiver to provide clarification
Obtain family’s reaction or thoughts about the diagnosis
N
F
F
O
R
GOAL:
Build alliance
M

## Slide 24
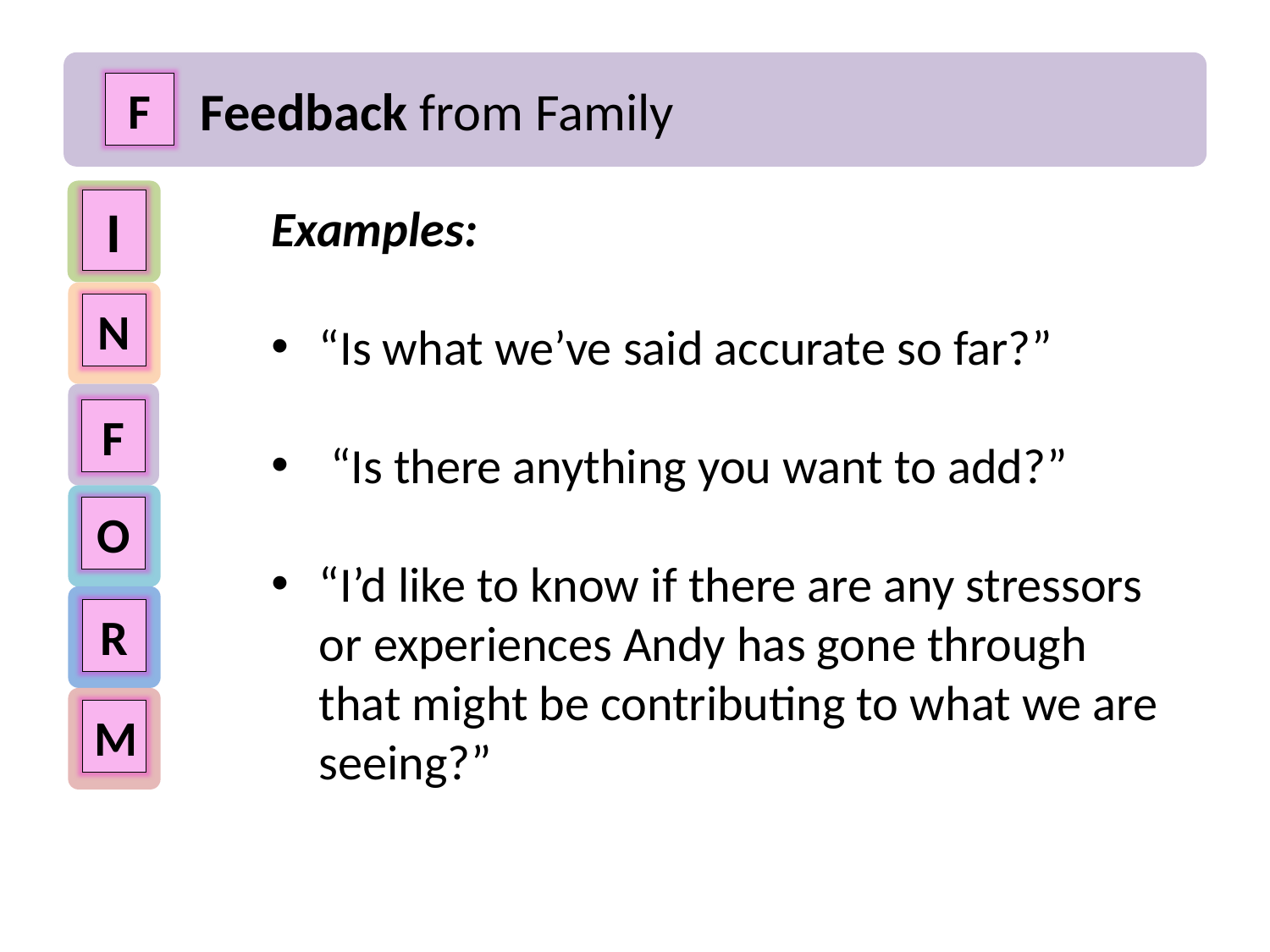

Feedback from Family
Step 3: Feedback from family
F
I
Examples:
“Is what we’ve said accurate so far?”
 “Is there anything you want to add?”
“I’d like to know if there are any stressors or experiences Andy has gone through that might be contributing to what we are seeing?”
N
F
F
O
R
M

## Slide 25
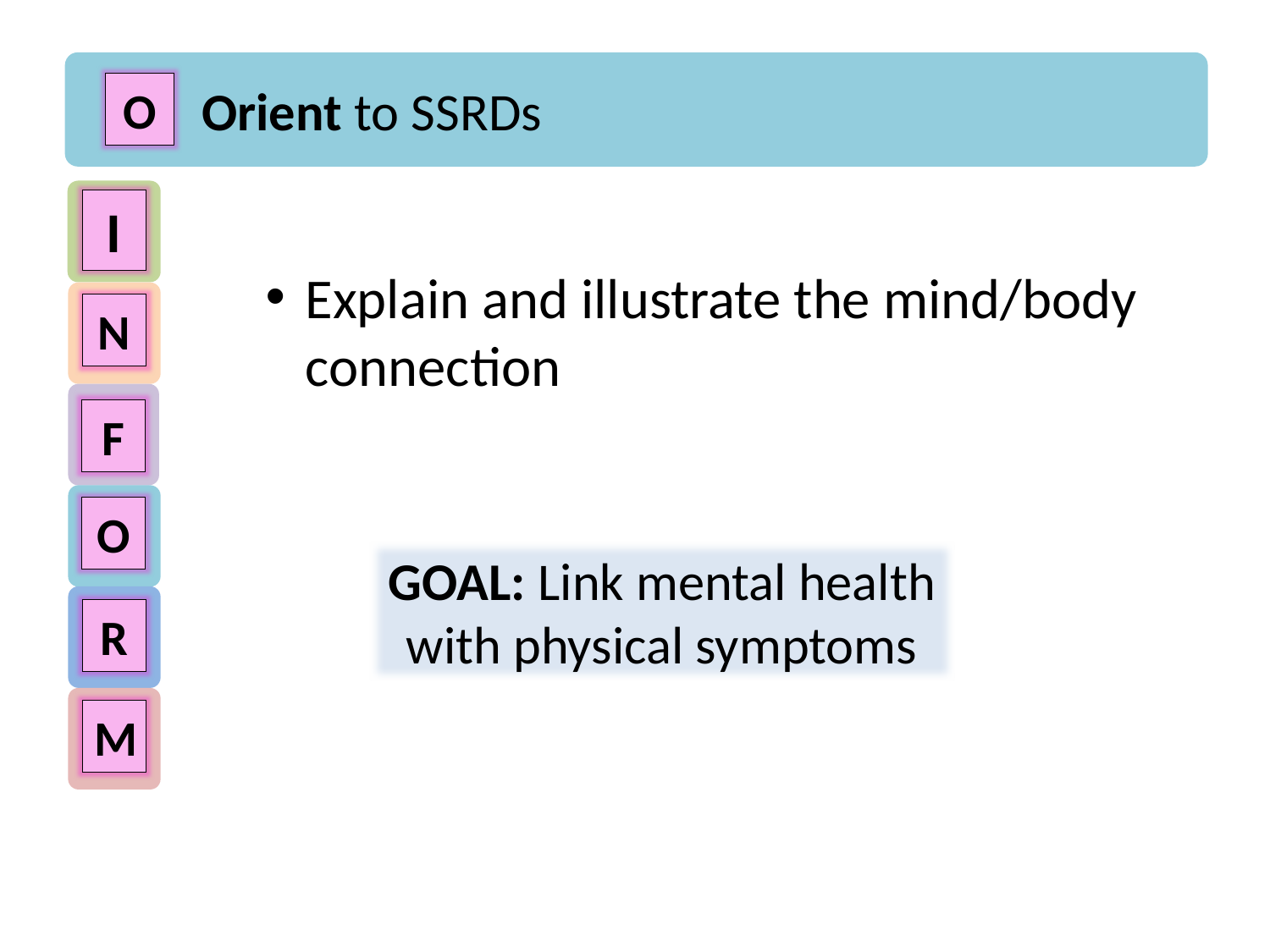

Orient to SSRDs
Step 4: Orient to diagnosis
O
I
Explain and illustrate the mind/body connection
N
F
O
O
GOAL: Link mental health with physical symptoms
R
M

## Slide 26
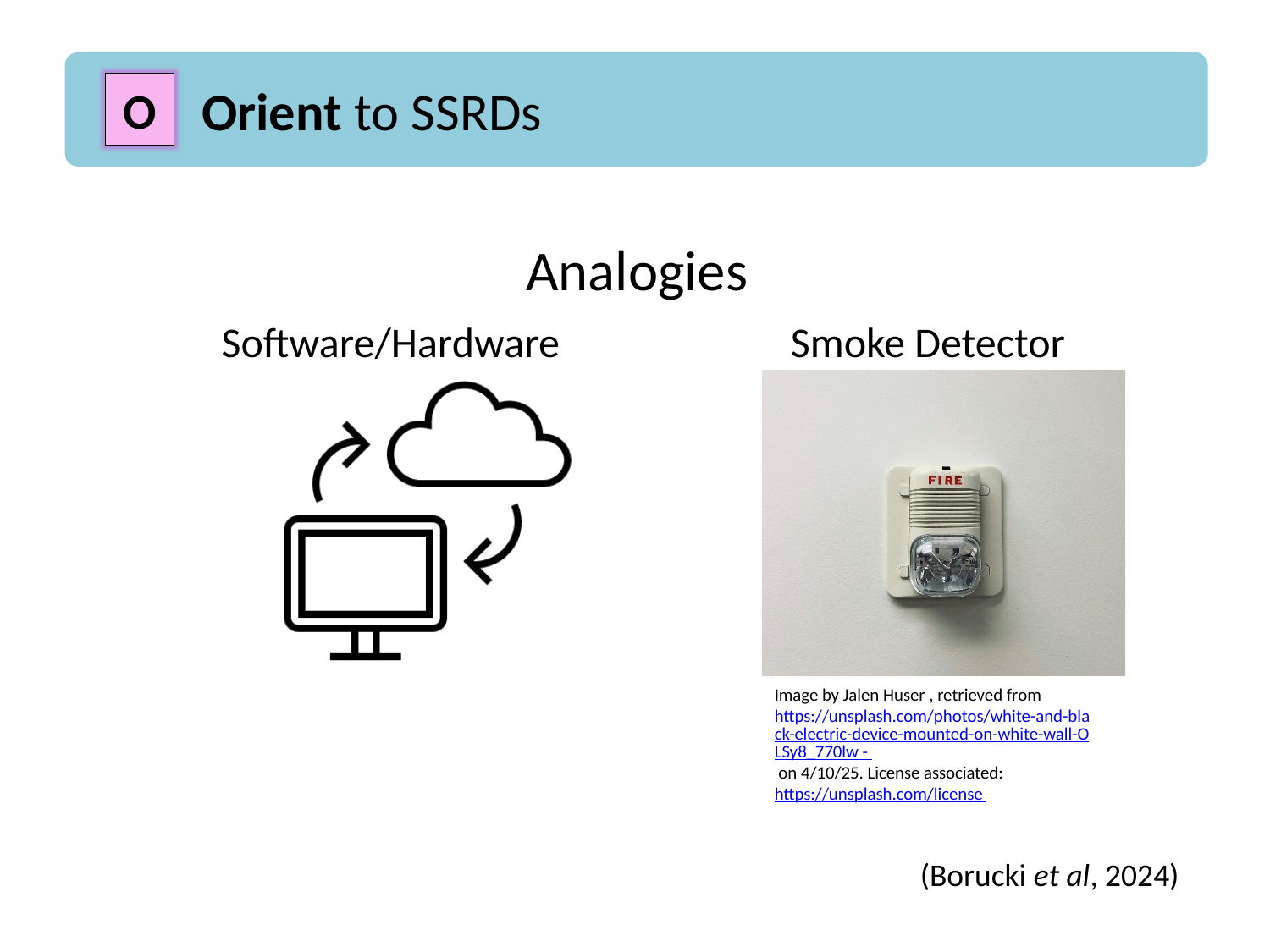

Orient to SSRDs
Step 4: Orient to diagnosis
O
Analogies
Software/Hardware
Smoke Detector
Image by Jalen Huser , retrieved from https://unsplash.com/photos/white-and-black-electric-device-mounted-on-white-wall-OLSy8_770lw - on 4/10/25. License associated: https://unsplash.com/license
(Borucki et al, 2024)

## Slide 27
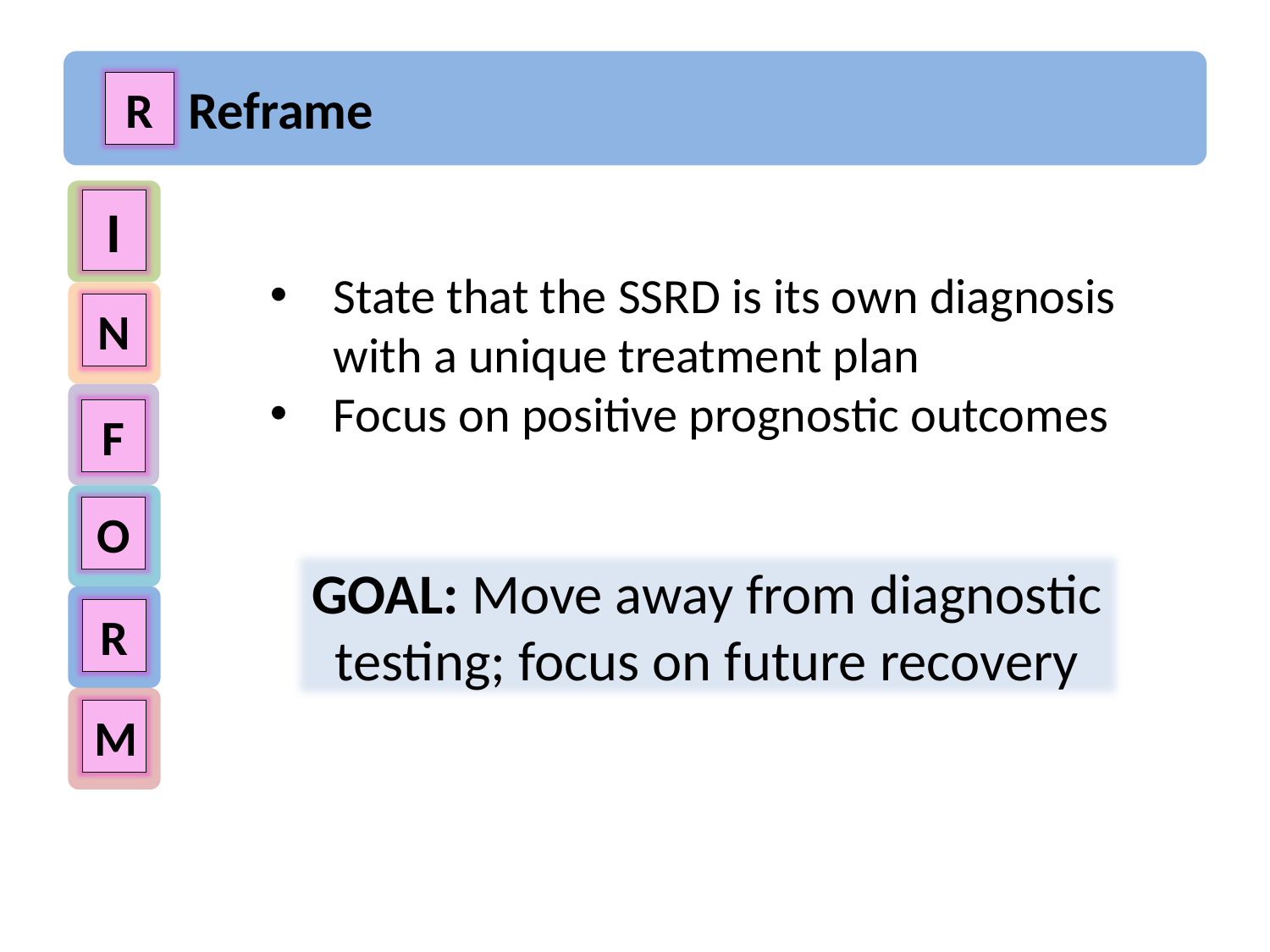

Reframe
R
I
State that the SSRD is its own diagnosis with a unique treatment plan
Focus on positive prognostic outcomes
N
F
O
GOAL: Move away from diagnostic testing; focus on future recovery
R
R
M

## Slide 28
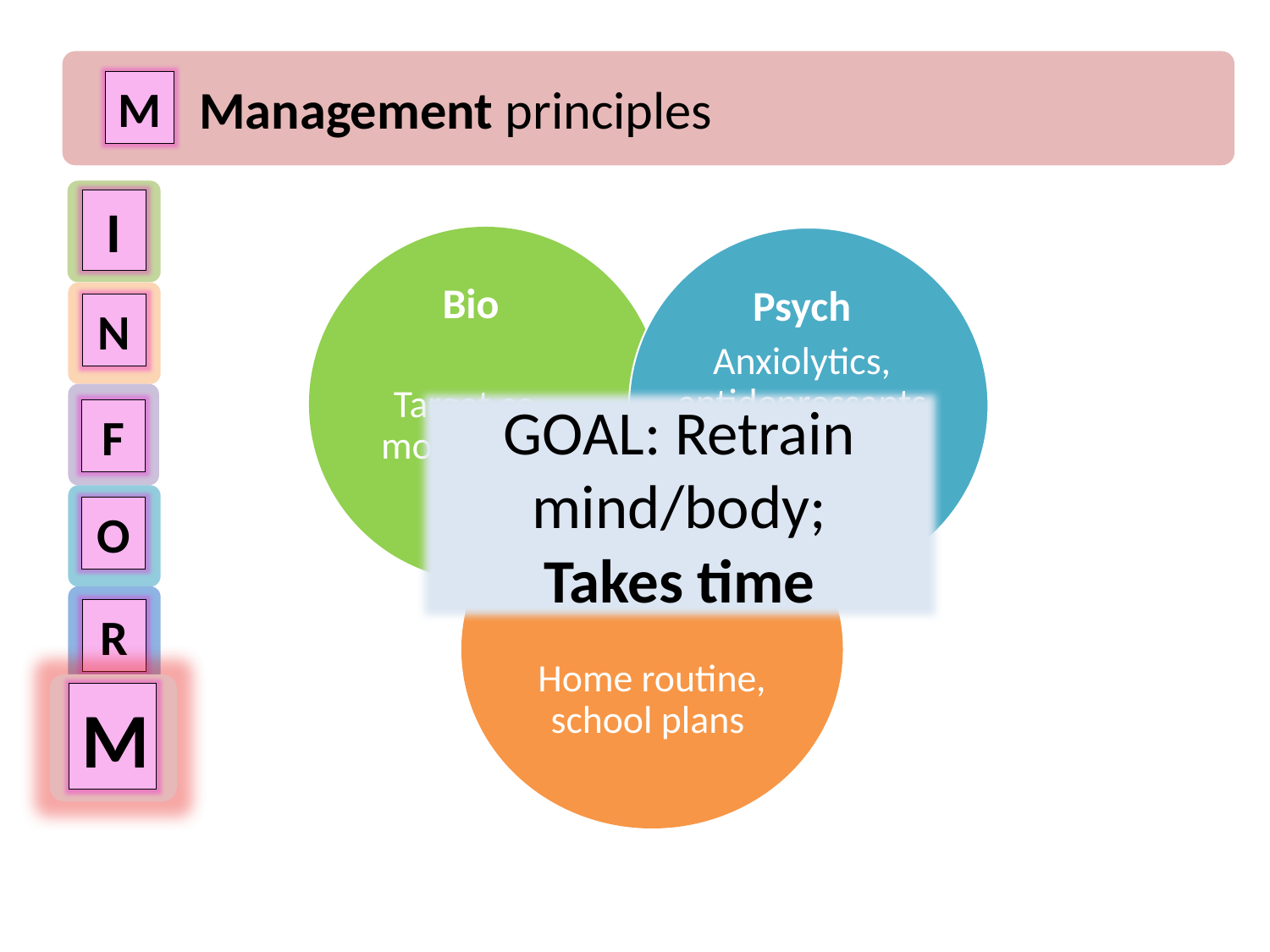

Management principles
M
I
Social
Home routine, school plans
Bio
Target co-morbidities
Psych
Anxiolytics, antidepressants
Mental health therapy
N
F
GOAL: Retrain mind/body;
Takes time
O
R
M
M

## Slide 29
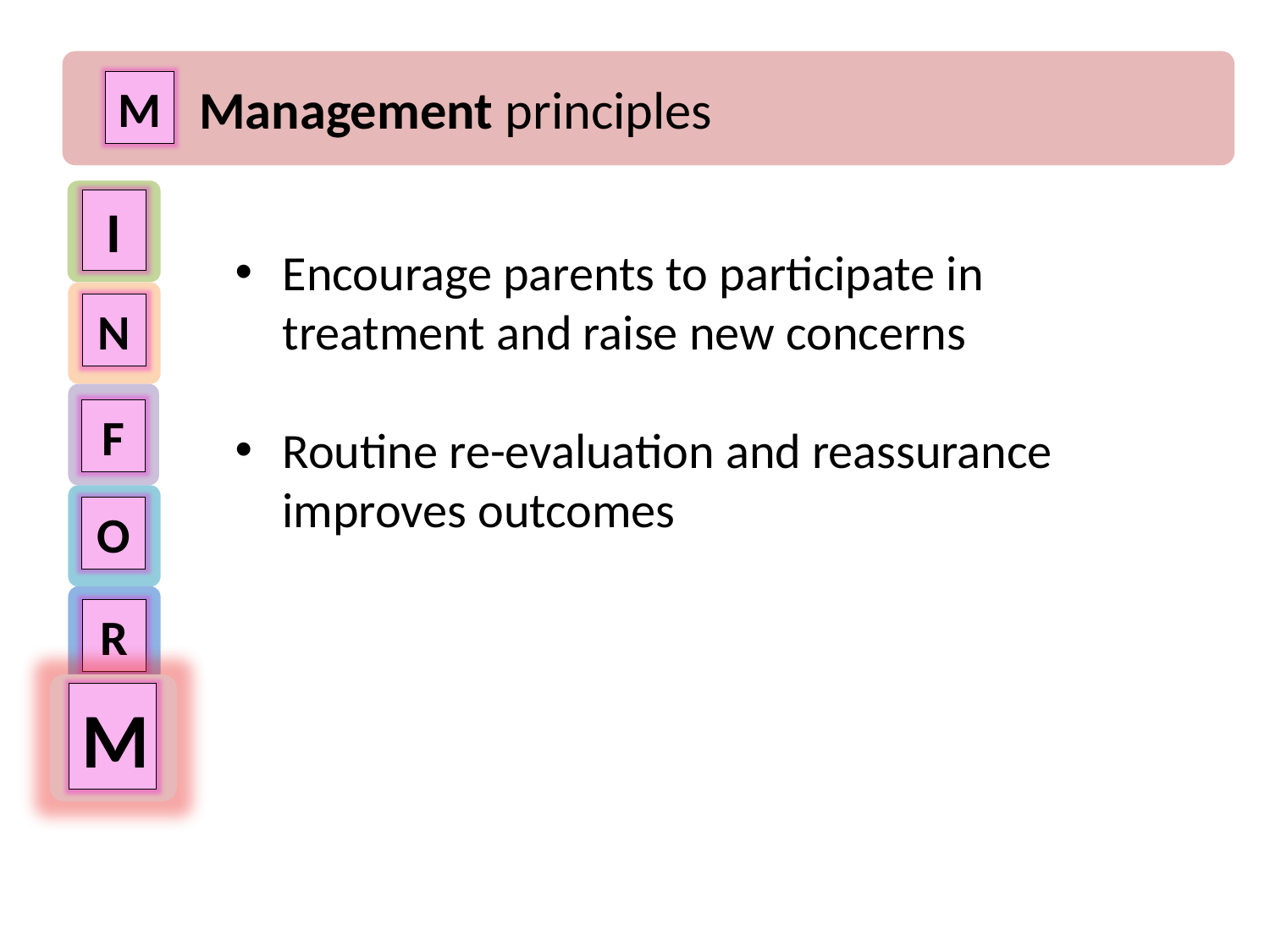

Management principles
M
I
Encourage parents to participate in treatment and raise new concerns
Routine re-evaluation and reassurance improves outcomes
N
F
O
R
M
M

## Slide 30
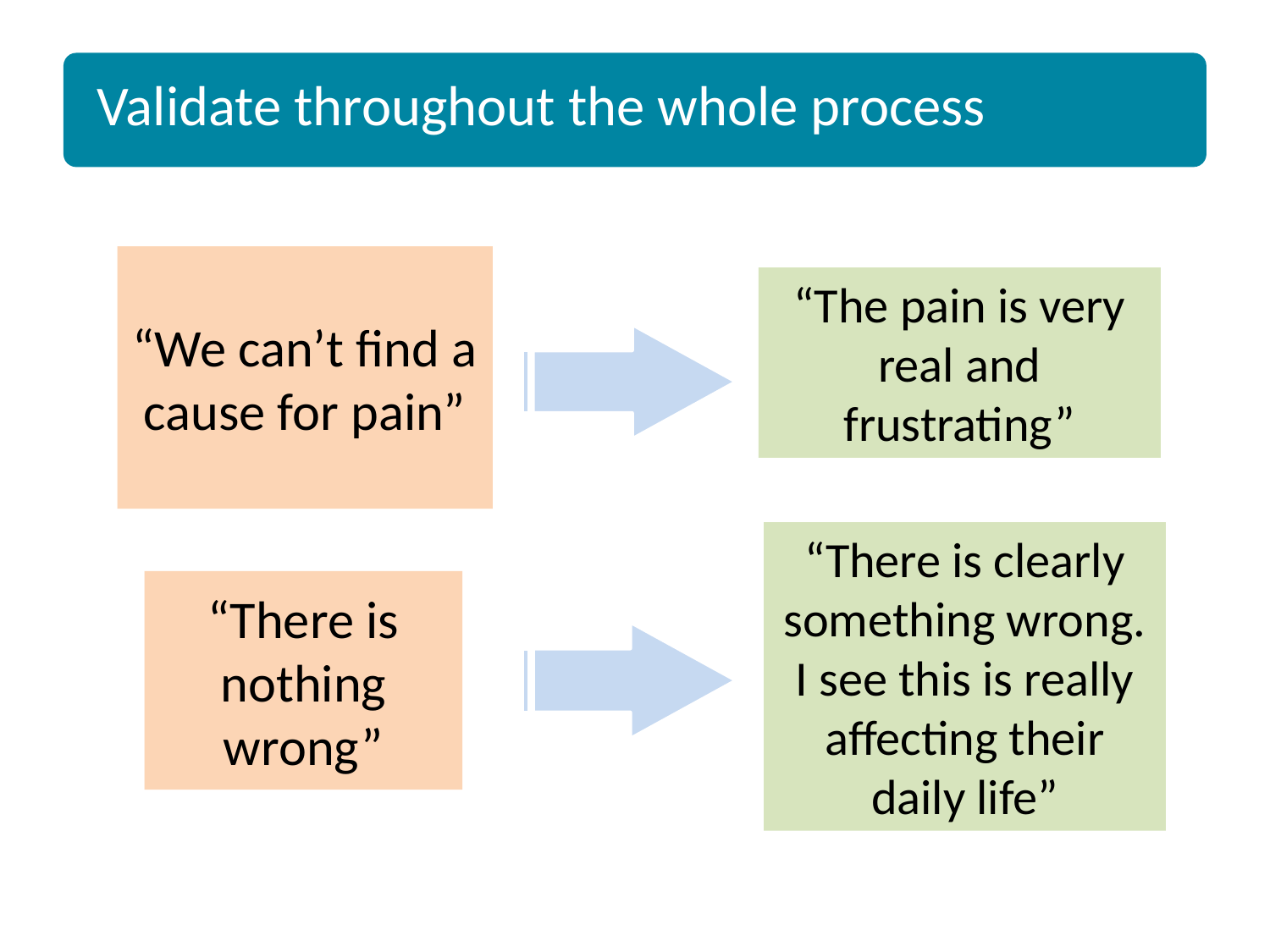

Validate throughout the whole process
“We can’t find a cause for pain”
“The pain is very real and frustrating”
“There is clearly something wrong. I see this is really affecting their daily life”
“There is nothing wrong”

## Slide 31
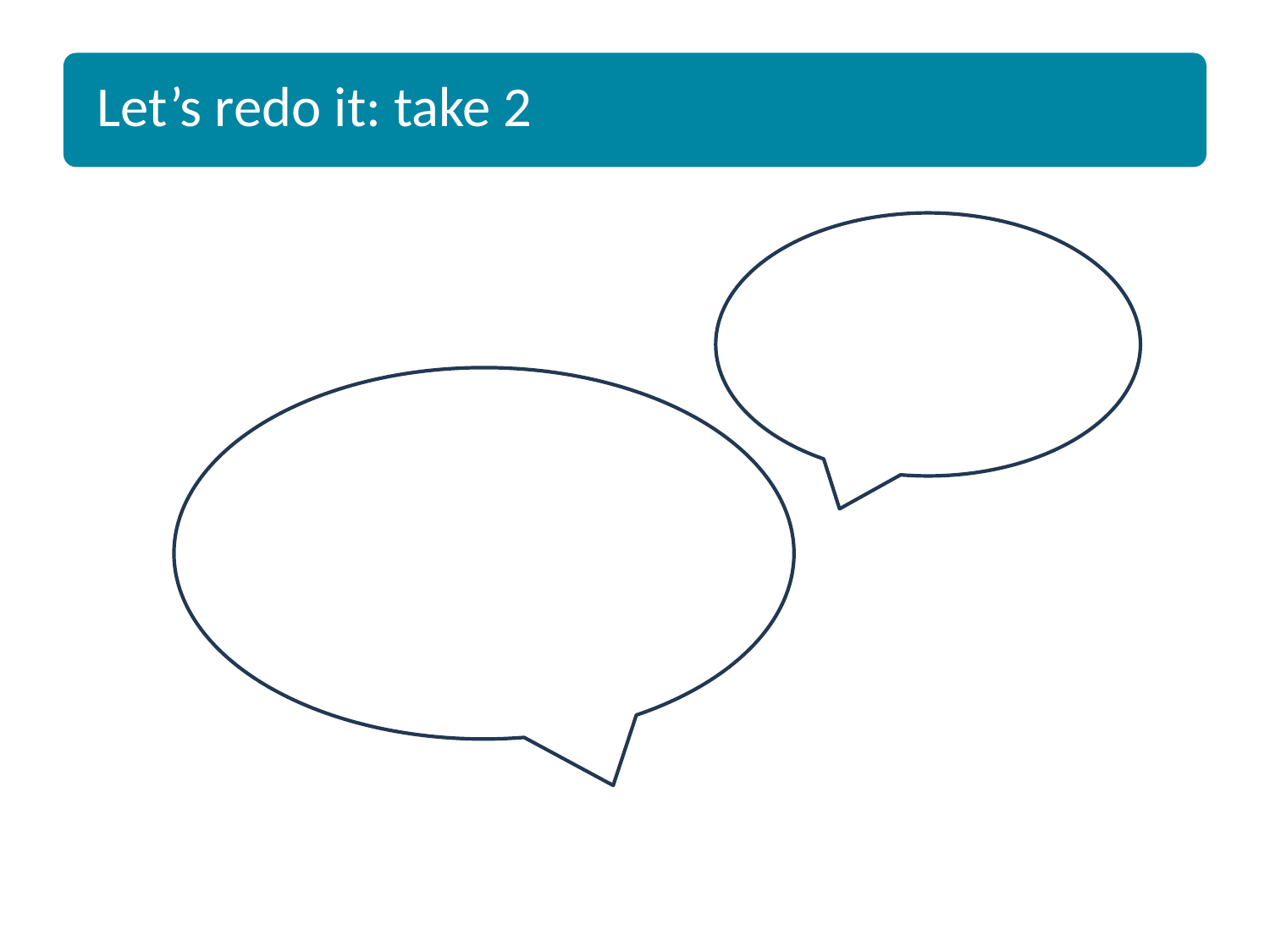

Let’s redo it: take 2

## Slide 32
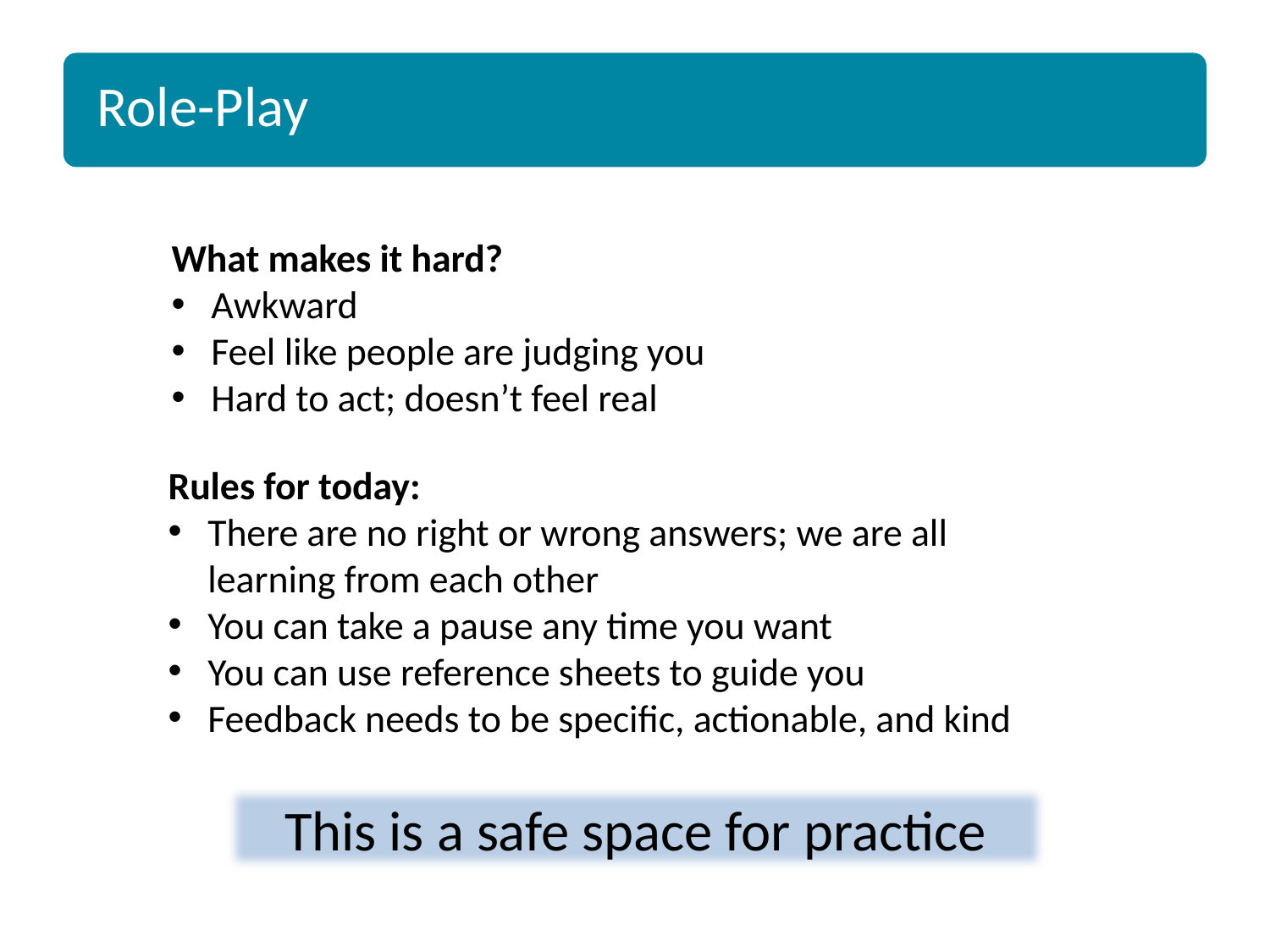

Role-Play
What makes it hard?
Awkward
Feel like people are judging you
Hard to act; doesn’t feel real
Rules for today:
There are no right or wrong answers; we are all learning from each other
You can take a pause any time you want
You can use reference sheets to guide you
Feedback needs to be specific, actionable, and kind
This is a safe space for practice

## Slide 33
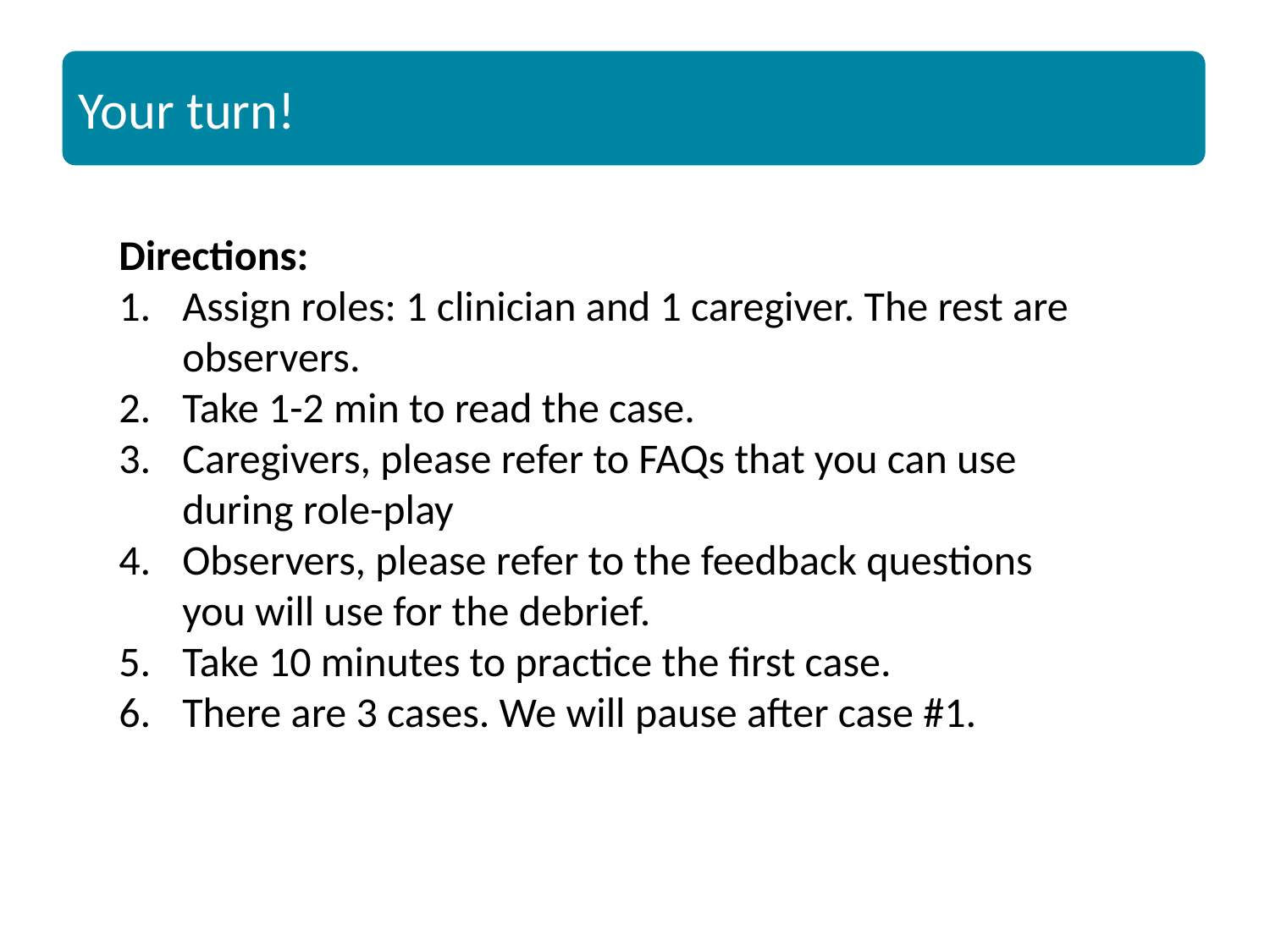

Your turn!
Directions:
Assign roles: 1 clinician and 1 caregiver. The rest are observers.
Take 1-2 min to read the case.
Caregivers, please refer to FAQs that you can use during role-play
Observers, please refer to the feedback questions you will use for the debrief.
Take 10 minutes to practice the first case.
There are 3 cases. We will pause after case #1.

## Slide 34
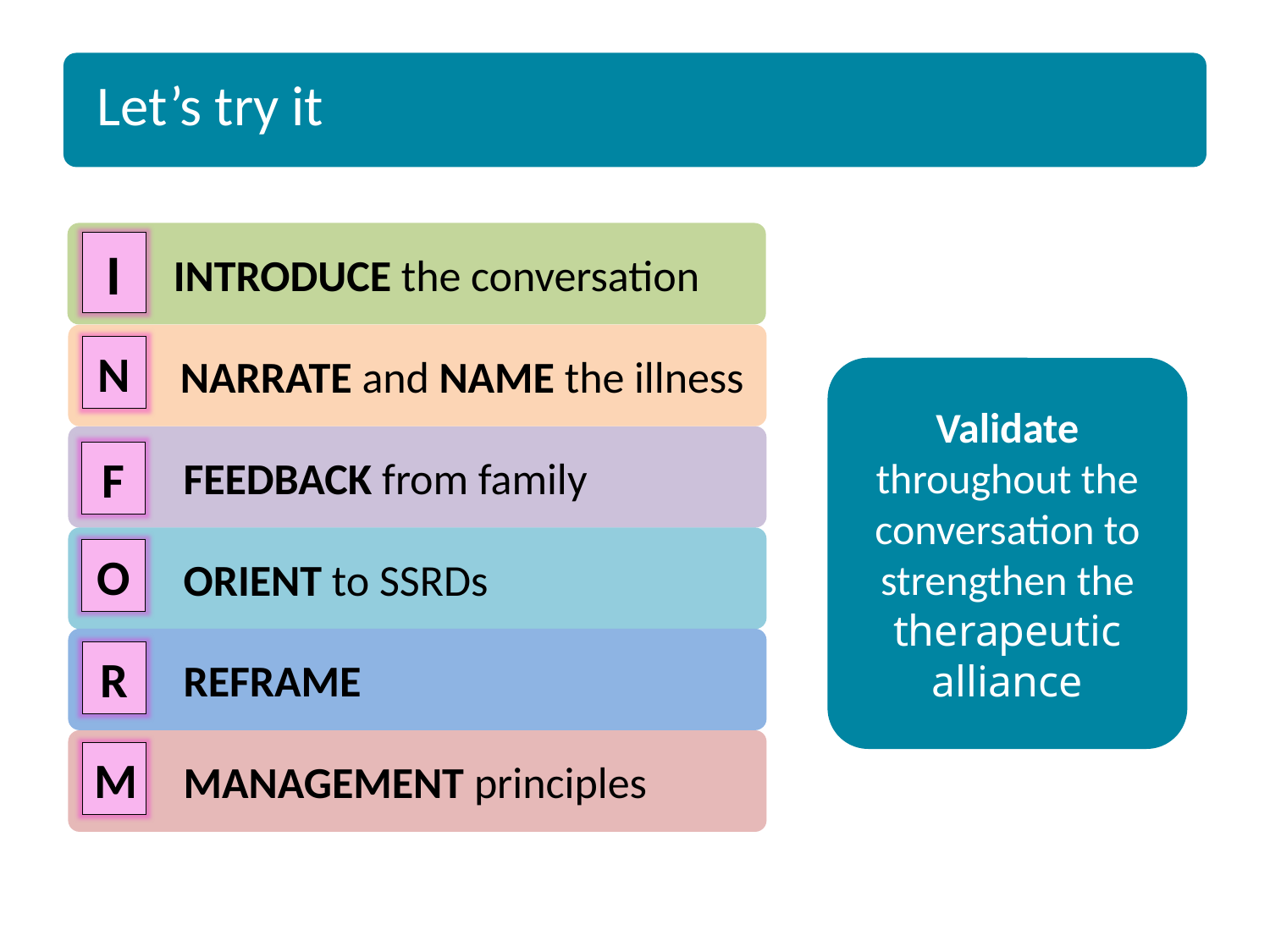

Let’s try it
 INTRODUCE the conversation
I
 NARRATE and NAME the illness
N
Validate throughout the conversation to strengthen the therapeutic alliance
 FEEDBACK from family
F
 ORIENT to SSRDs
O
 REFRAME
R
 MANAGEMENT principles
M

## Slide 35
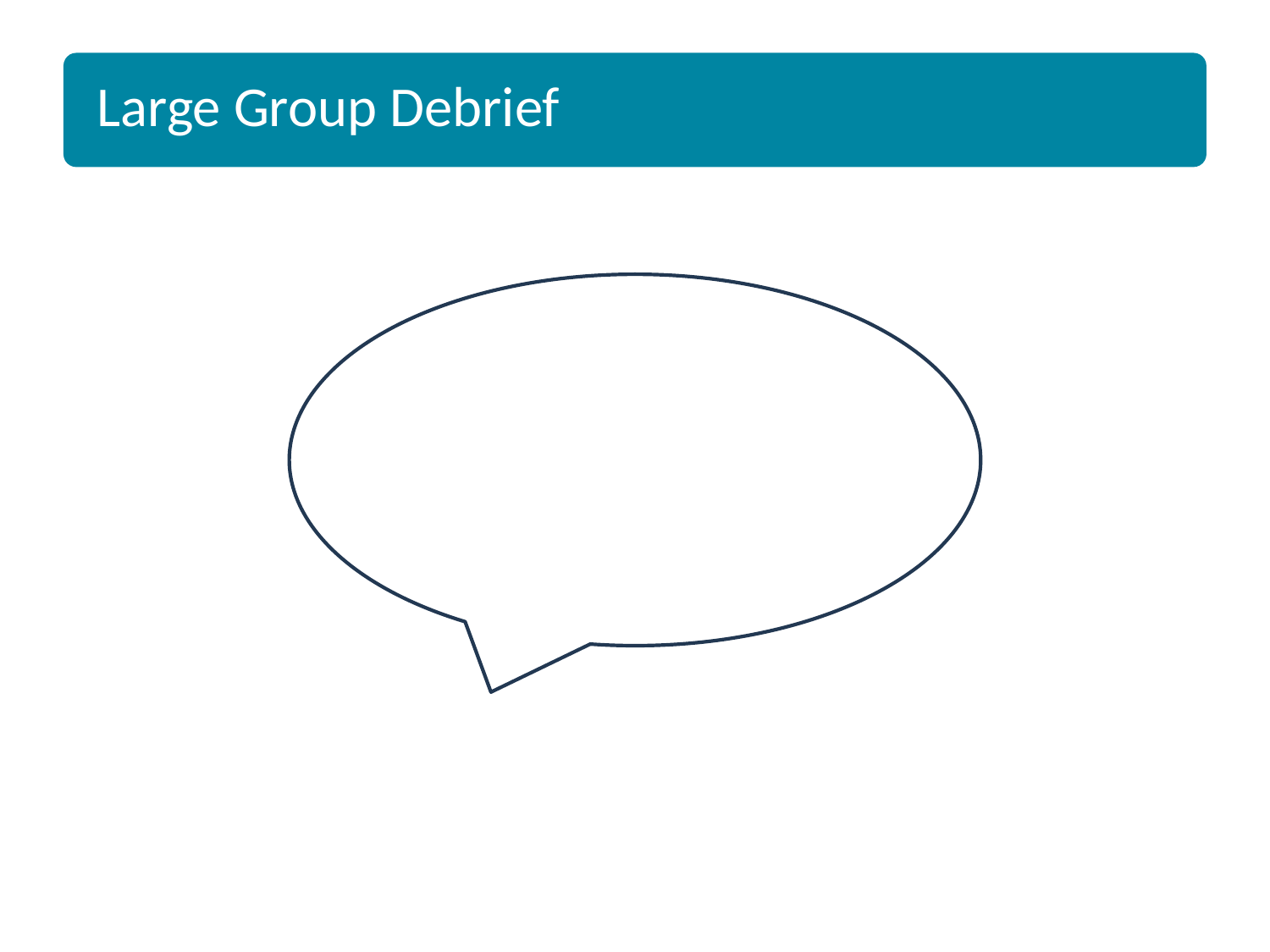

Large Group Debrief

## Slide 36
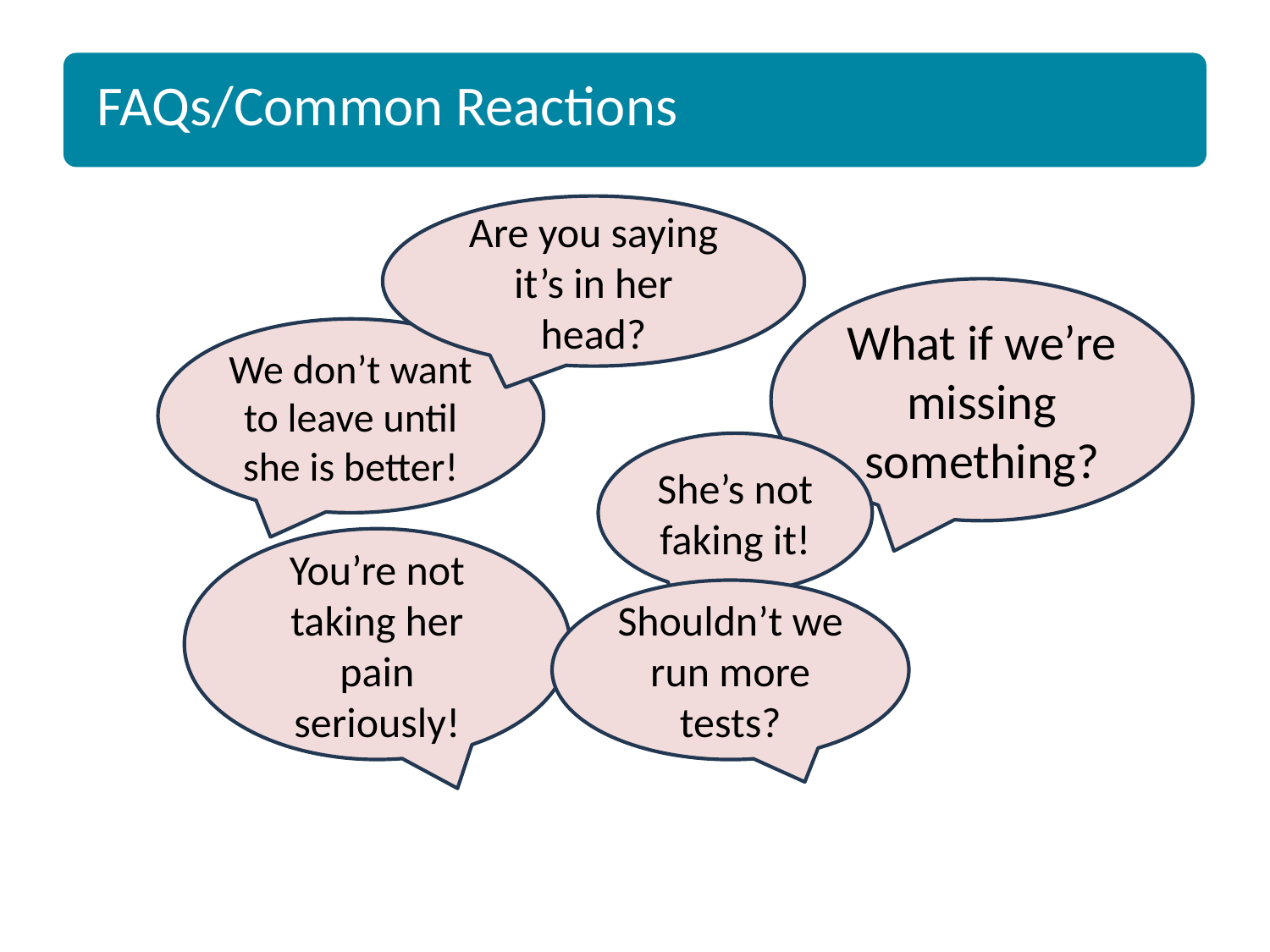

FAQs/Common Reactions
Are you saying it’s in her head?
What if we’re missing something?
We don’t want to leave until she is better!
She’s not faking it!
You’re not taking her pain seriously!
Shouldn’t we run more tests?

## Slide 37
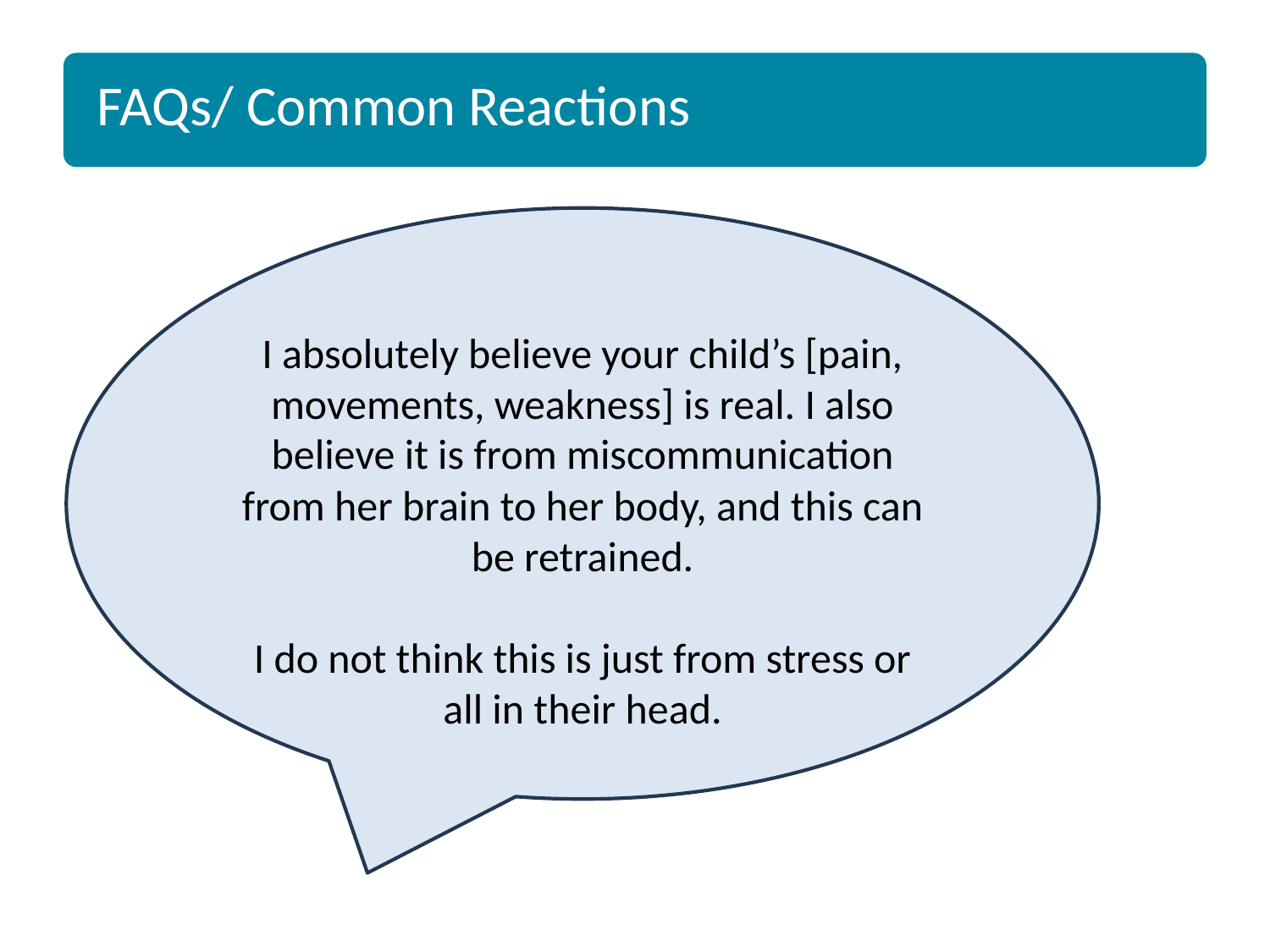

FAQs/ Common Reactions
I absolutely believe your child’s [pain, movements, weakness] is real. I also believe it is from miscommunication from her brain to her body, and this can be retrained.
I do not think this is just from stress or all in their head.
Are you saying it’s in her head?
She’s not faking it!
You’re not taking her pain seriously!

## Slide 38
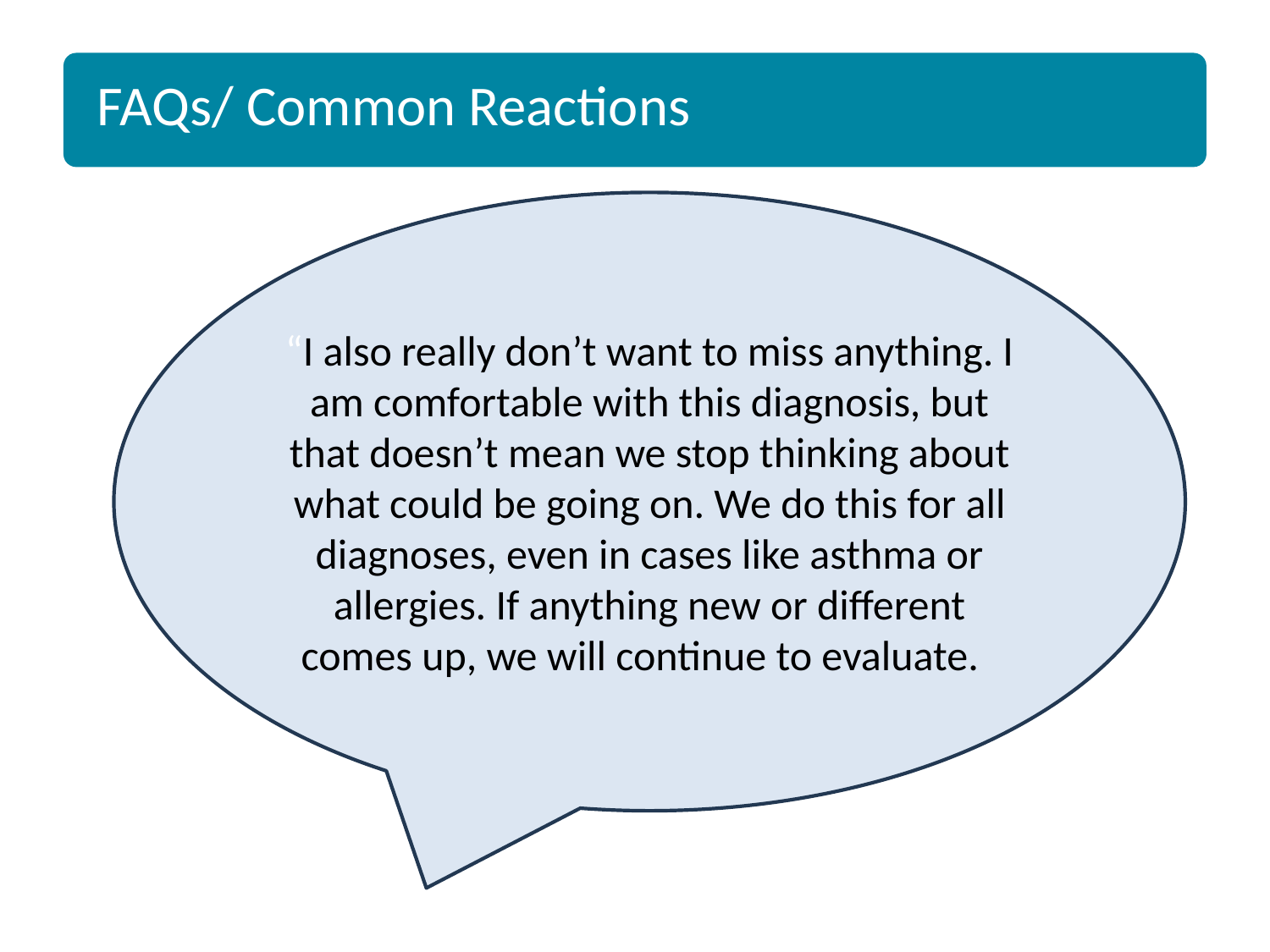

FAQs/ Common Reactions
“I also really don’t want to miss anything. I am comfortable with this diagnosis, but that doesn’t mean we stop thinking about what could be going on. We do this for all diagnoses, even in cases like asthma or allergies. If anything new or different comes up, we will continue to evaluate.
What if we’re missing something?
Shouldn’t we run more tests?

## Slide 39
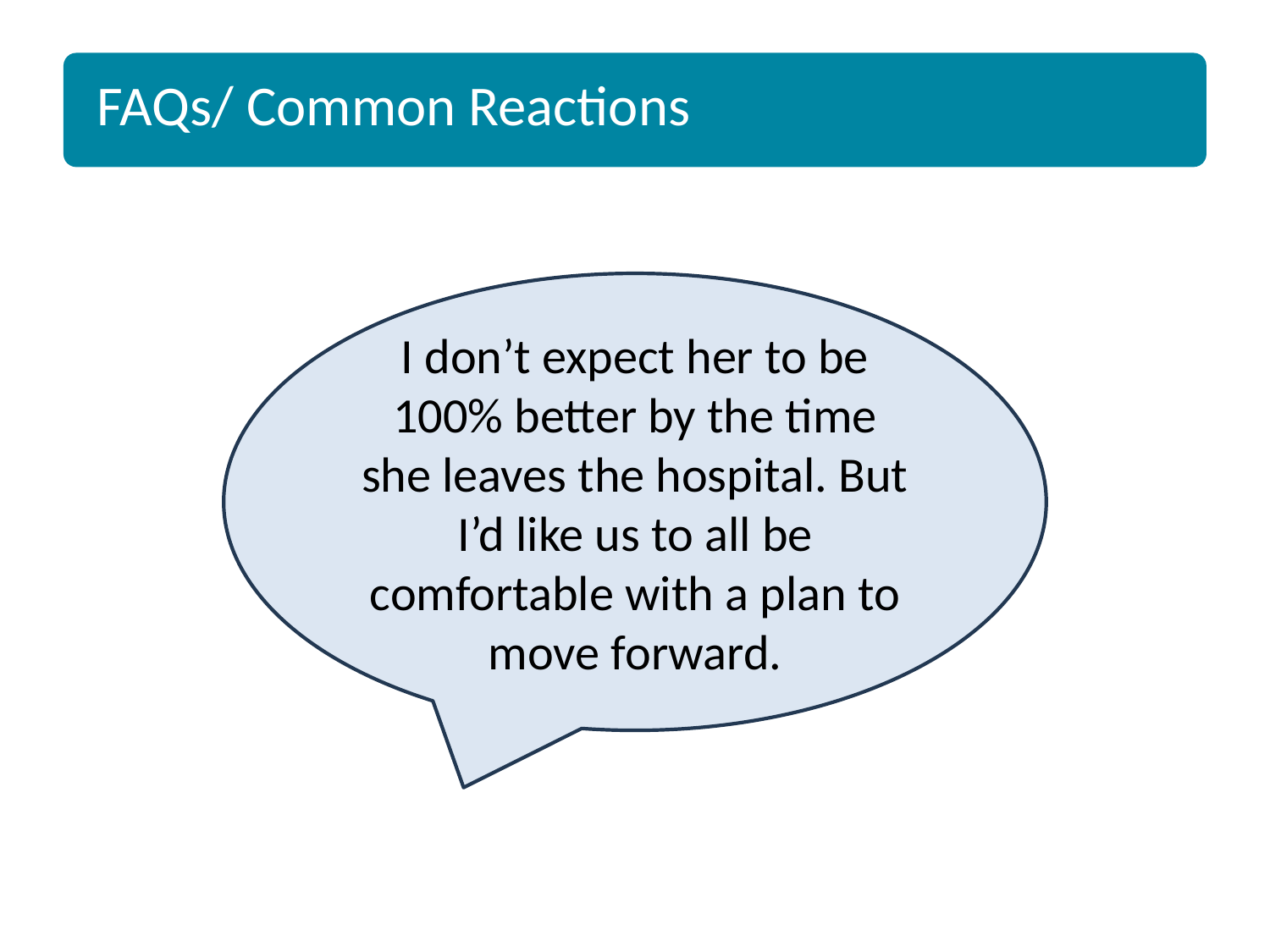

FAQs/ Common Reactions
I don’t expect her to be 100% better by the time she leaves the hospital. But I’d like us to all be comfortable with a plan to move forward.
We don’t want to leave until she is better!

## Slide 40
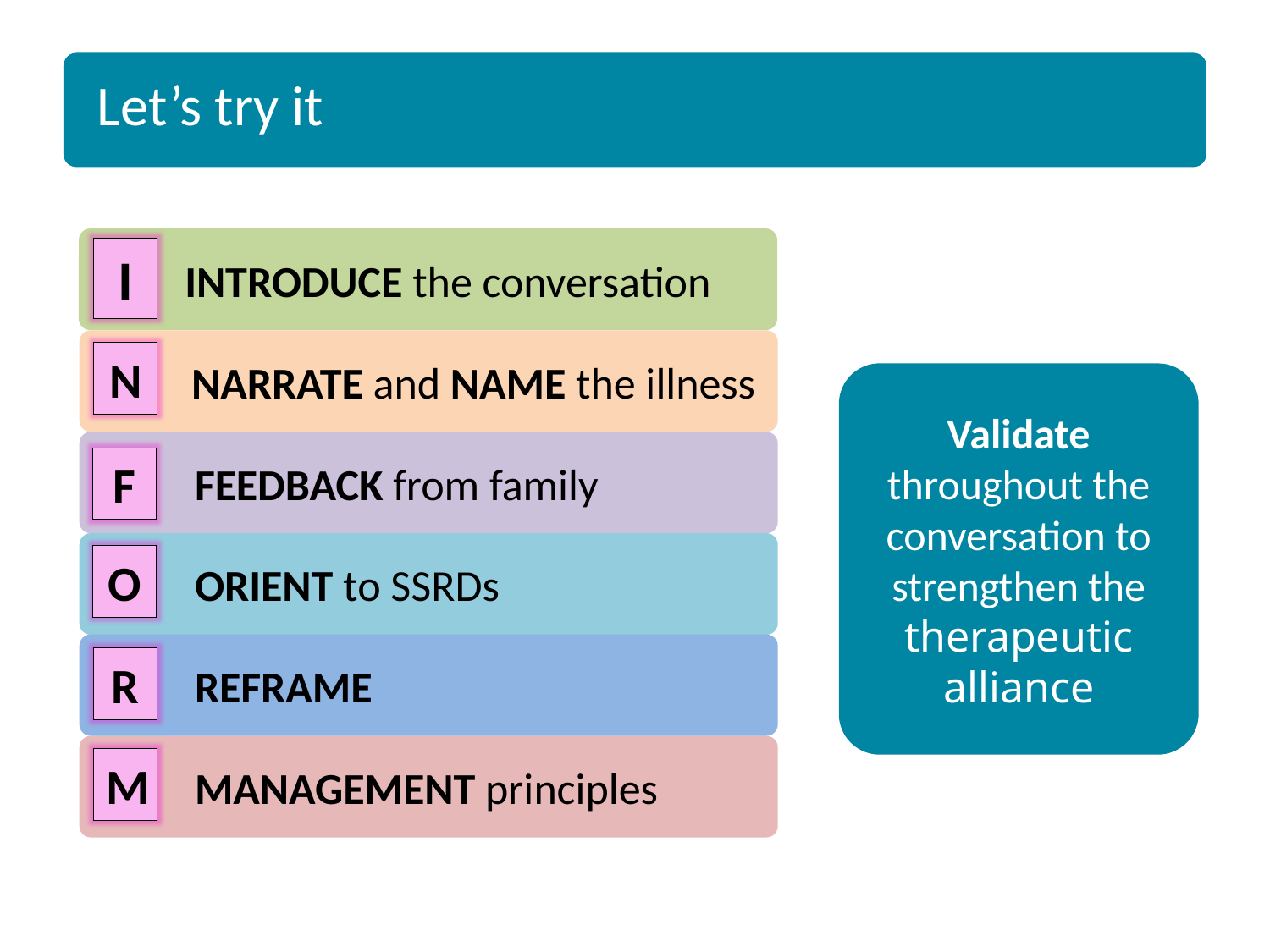

Let’s try it
 INTRODUCE the conversation
I
 NARRATE and NAME the illness
N
Validate throughout the conversation to strengthen the therapeutic alliance
 FEEDBACK from family
F
 ORIENT to SSRDs
O
 REFRAME
R
 MANAGEMENT principles
M

## Slide 41
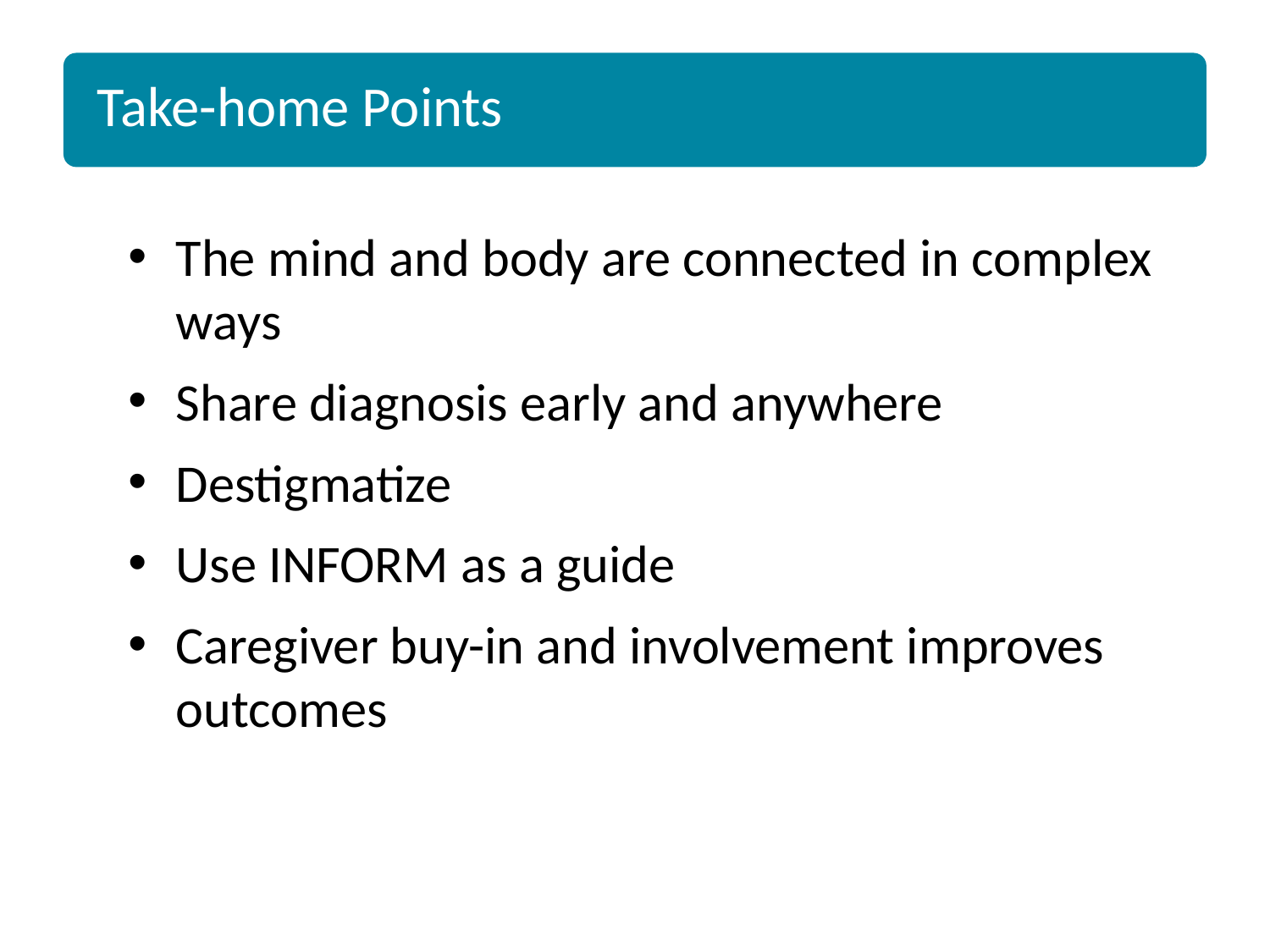

Take-home Points
The mind and body are connected in complex ways
Share diagnosis early and anywhere
Destigmatize
Use INFORM as a guide
Caregiver buy-in and involvement improves outcomes

## Slide 42
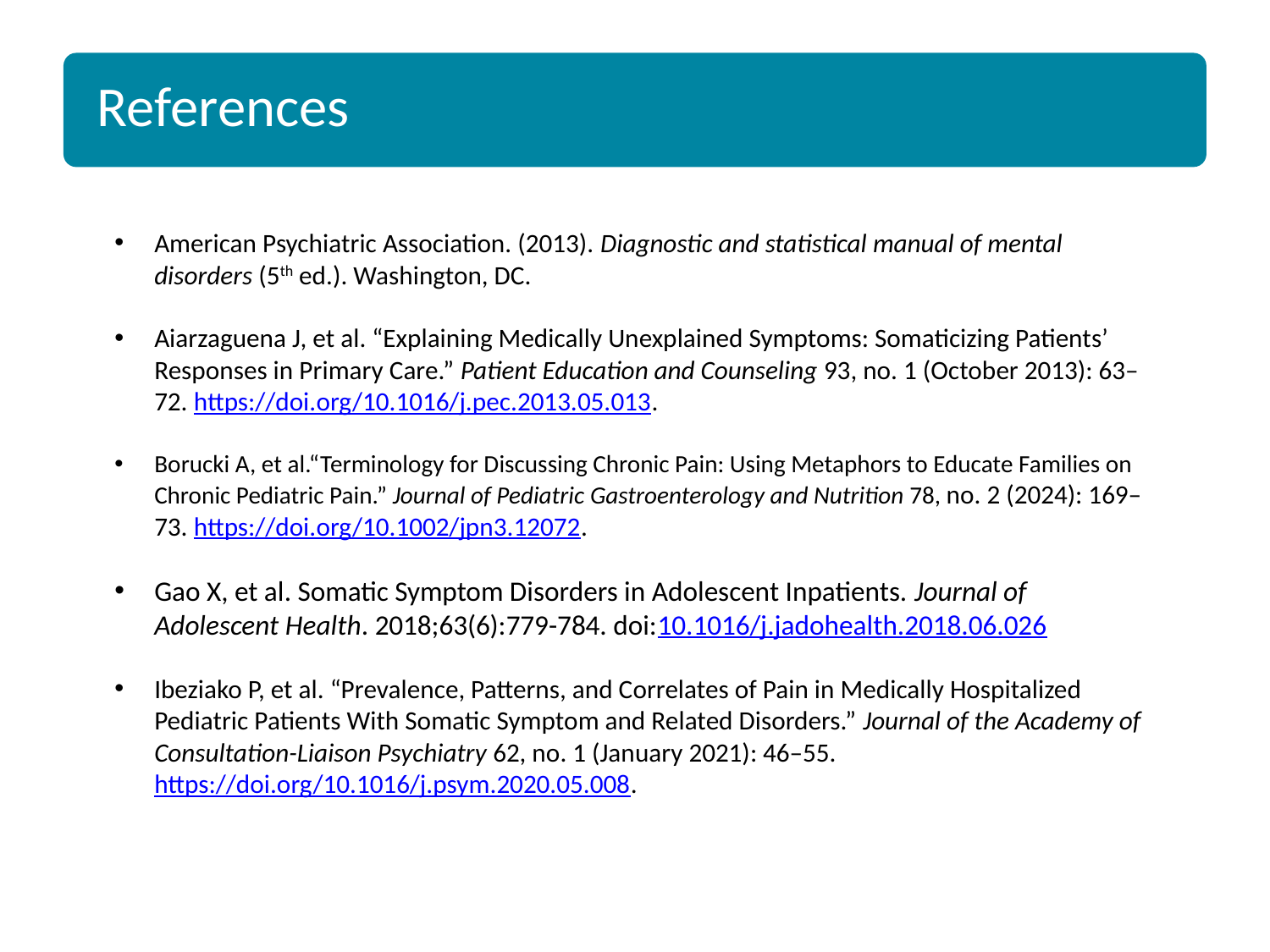

References
American Psychiatric Association. (2013). Diagnostic and statistical manual of mental disorders (5th ed.). Washington, DC.
Aiarzaguena J, et al. “Explaining Medically Unexplained Symptoms: Somaticizing Patients’ Responses in Primary Care.” Patient Education and Counseling 93, no. 1 (October 2013): 63–72. https://doi.org/10.1016/j.pec.2013.05.013.
Borucki A, et al.“Terminology for Discussing Chronic Pain: Using Metaphors to Educate Families on Chronic Pediatric Pain.” Journal of Pediatric Gastroenterology and Nutrition 78, no. 2 (2024): 169–73. https://doi.org/10.1002/jpn3.12072.
Gao X, et al. Somatic Symptom Disorders in Adolescent Inpatients. Journal of Adolescent Health. 2018;63(6):779-784. doi:10.1016/j.jadohealth.2018.06.026
Ibeziako P, et al. “Prevalence, Patterns, and Correlates of Pain in Medically Hospitalized Pediatric Patients With Somatic Symptom and Related Disorders.” Journal of the Academy of Consultation-Liaison Psychiatry 62, no. 1 (January 2021): 46–55. https://doi.org/10.1016/j.psym.2020.05.008.

## Slide 43
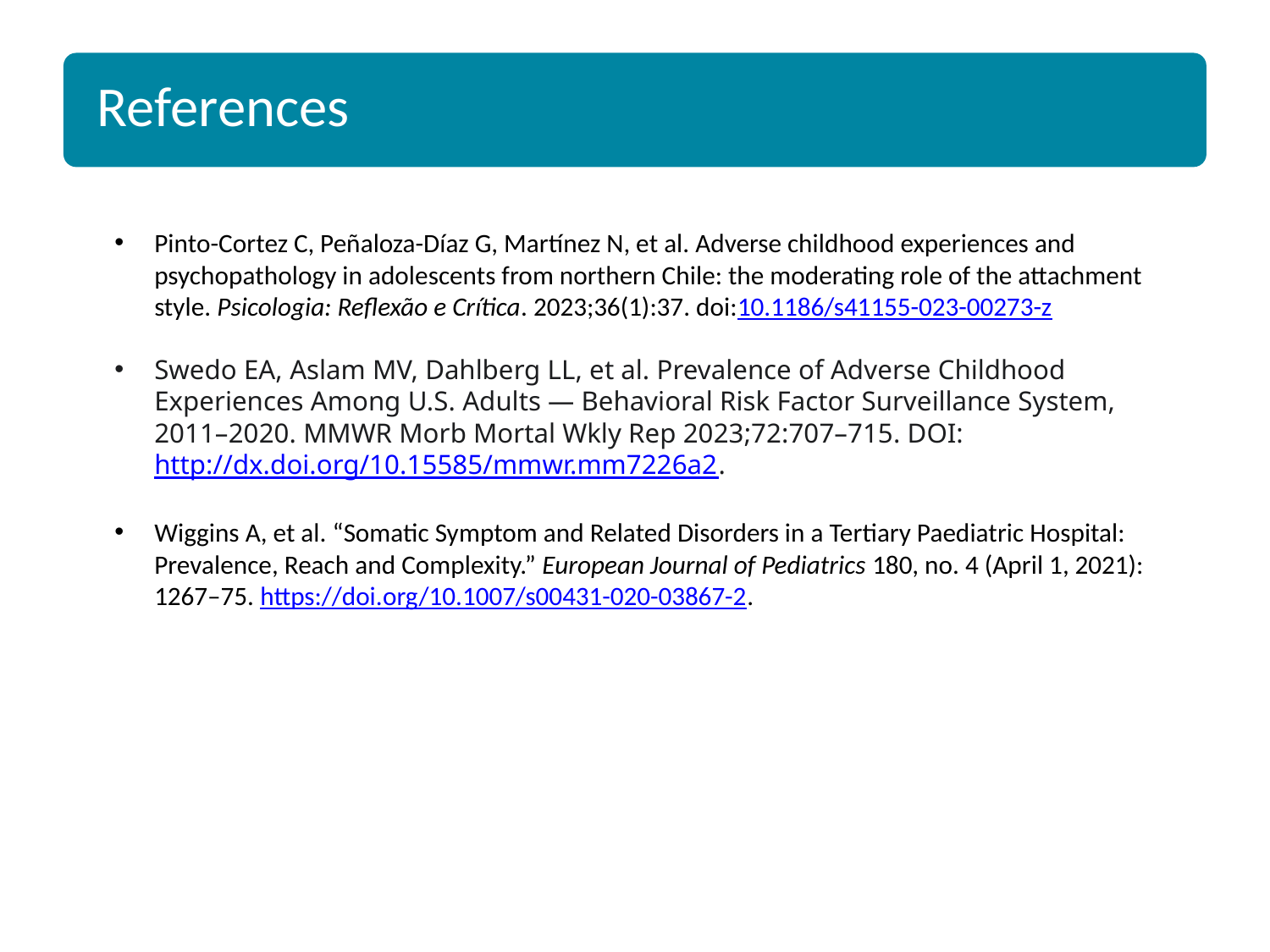

References
Pinto-Cortez C, Peñaloza-Díaz G, Martínez N, et al. Adverse childhood experiences and psychopathology in adolescents from northern Chile: the moderating role of the attachment style. Psicologia: Reflexão e Crítica. 2023;36(1):37. doi:10.1186/s41155-023-00273-z
Swedo EA, Aslam MV, Dahlberg LL, et al. Prevalence of Adverse Childhood Experiences Among U.S. Adults — Behavioral Risk Factor Surveillance System, 2011–2020. MMWR Morb Mortal Wkly Rep 2023;72:707–715. DOI: http://dx.doi.org/10.15585/mmwr.mm7226a2.
Wiggins A, et al. “Somatic Symptom and Related Disorders in a Tertiary Paediatric Hospital: Prevalence, Reach and Complexity.” European Journal of Pediatrics 180, no. 4 (April 1, 2021): 1267–75. https://doi.org/10.1007/s00431-020-03867-2.

## Slide 44
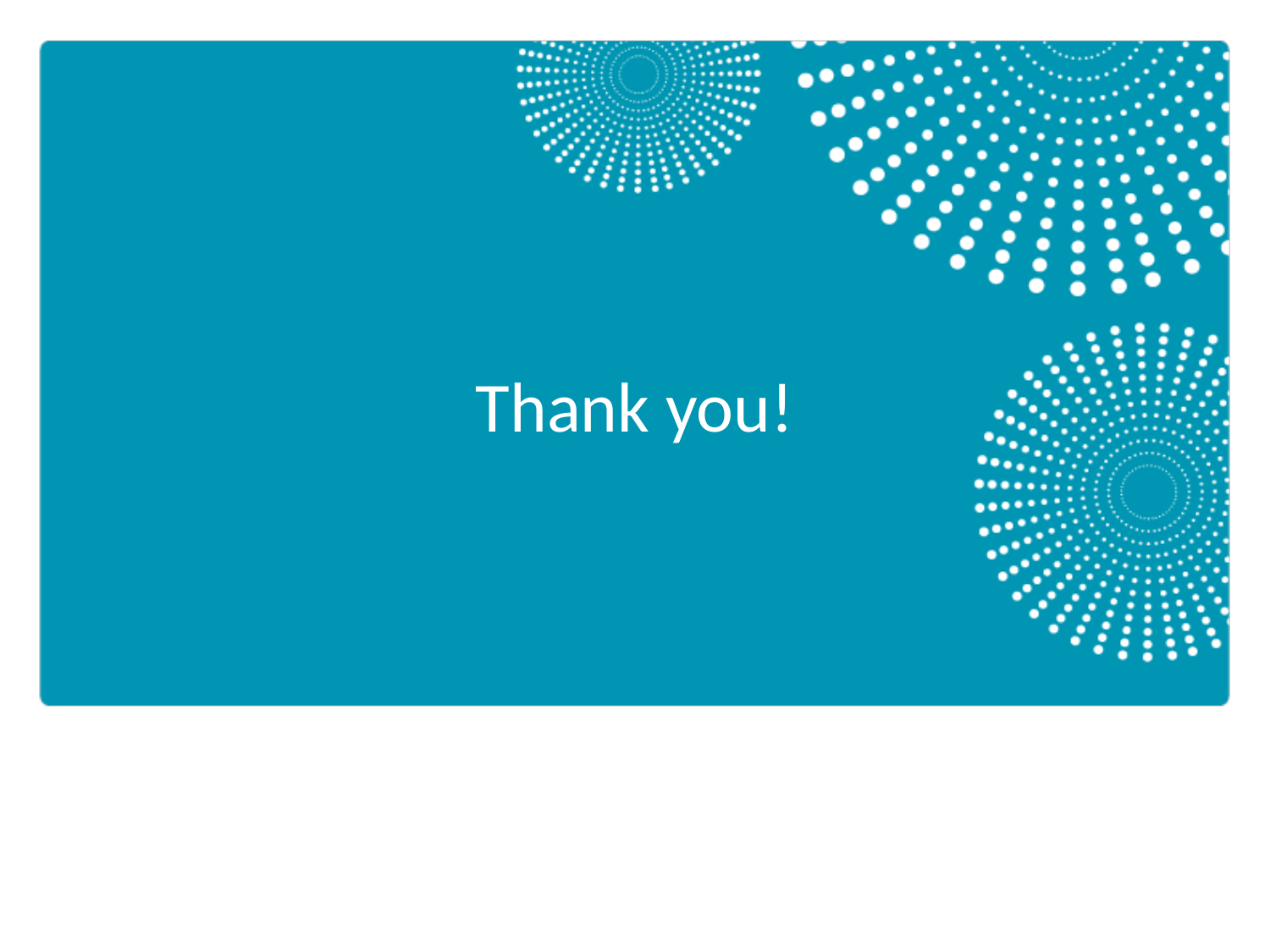

Thank you!
