# Supplementary material for: INFORM: A Pediatrician's Communication Curriculum About Diagnostic Conversations in Somatic Symptom and Related Disorders
Source: MedEdPORTAL. 2025 Dec 2;21:11561. doi: 10.15766/mep_2374-8265.11561 (PMC12669383; doi:10.15766/mep_2374-8265.11561)
Supplement: Supplementary file 1 — Curriculum Agenda.docxSlide Deck With Script.pptxScript for Case Demonstration by Facilitators.docxCases for Role-Play.docxObserver and Caregiver Guide for Role-Play.docxINFORM Quick Guide.docxGlossary of Acronyms.docxCurriculum Evaluation Forms.docx [file mep_2374-8265.11561-s001.zip › C. Script for Case Demonstration by Facilitators.docx]

**Functional Abdominal Pain Case – Bad Demo/Good Demo**

**Role play by two facilitators**

**“Bad” Demonstration (Slide #13):**

*Clinician*: Hi, we are here for rounds. It sounds like your child has complained of pain for a few months. You have been to the emergency room multiple times, and now you are admitted. We have done about every test we can think of, and everything looks normal.

*Caregiver:* Yes, we’ve been dealing with this issue for a long time. I’m hoping we have answers. She’s really miserable, and I don’t know what else to do.

*Clinician:* Well, her abdomen is totally soft; there’s no real pain, which is great. Everything looks good; nothing is wrong with her.

*Caregiver:* What do you mean? She is in terrible pain to the point she was admitted to the hospital. How do you plan to figure out what is going on and fix this?

*Clinician:* We haven’t found anything specific to fix. Typically, in these situations, we find that stress and other factors can make a child feel abdominal pain rather than something medical. So I am not sure what we’ll be able to do for you in the hospital.

*Caregiver:* Are you saying she is just stressed? That this is all in her head? She has been in excruciating pain for months. This isn’t a mental health problem.

*Clinician:* I’m not saying that exactly, but maybe stress is contributing to things.

*Caregiver:* I feel like you might be missing something; I want another doctor in here.

*Clinician:* There isn’t anyone else that can see you; she should follow up with primary care after discharge.

*Caregiver:* Her primary care doctor keeps telling us there is nothing more they can do and to come to the emergency room. I’m not leaving without more answers – do whatever you need to do. I want more imaging and labs, and she should be getting pain medications.

**“Good” Demonstration of the INFORM framework (Slide #30):**

*Clinician:* *[****INTRODUCE]*** We are the medical team here, circling back to talk about Andy’s symptoms and diagnosis. Is now a good time for us to talk?

*Caregiver:* Yes, now is a good time.

*Clinician:* *[****NARRATE]*** Let’s go over everything together first. Andy’s symptoms started a gastroenteritis when you were on that cruise in the summer. Everyone in the family got the stomach bug and recovered, but Andy has continued to have abdominal pain, as well as vomiting a couple times per week since then. The pain has come and gone, although sometimes it can last a whole day. Is that correct?

*Caregiver:* Yes, that’s correct, and she’s also been dizzy from time to time. It’s really scary.

*Clinician:* Oh yes, that’s right. It does sound really scary; I can’t imagine what you have been going through. From what I remember, she has had an abdominal and pelvic CT scan, which were normal. She has had bloodwork that has looked for infection, that looked at her liver and kidney function and urine, and all of these have been reassuring. These tests have ruled out things like a blockage in her GI tract, cancer, infection, or an inflammatory condition such as Crohn’s Disease or colitis.

*Caregiver:* Yes, every test so far is normal, so no one has figured out what it is yet.

*Clinician:* Yes you’ve been through a lot in the last few months, and it must be frustrating not to have answers. ***[NAME]*** However, I do think we have enough information to talk about her most likely diagnosis. Her picture to me seems more consistent with a disorder of gut-brain interaction, or DGBI. *[****FEEDBACK]*** Have you heard of this before?

*Caregiver:* No, I have no idea what that is.

*Clinician:* *[****ORIENT]*** In DGBIs, there is a miscommunication between the brain and the gut. There’s a miswiring that’s causing pain even with the virus no longer there. A little bit like if a fire alarm went off during a fire, and even though the fire was extinguished, the alarm is still going off.

Another analogy I like to give is that it’s like a volume dial. We see sometimes that patients have memory pain from prior illness, like a stomach bug, and it can cause children to have an amplification, or the volume to be “turned up” too loud even though all lab tests are normal. Additionally, in this diagnosis, stressful life events can cause even more amplification. Does this make sense?

*Caregiver:* Actually, Andy’s dad and I recently separated, and she is living between both houses. Maybe that can be part of it? Her father makes her go to school, and then I often have to pick her up early.

Does that mean the pain is in her head then?

*Clinician:* No, it is definitely not in her head. Her pain is very real, and I can tell it has been affecting her daily life.

*Caregiver:* It’s still hard to understand though. Are we sure we aren’t missing anything else? Aren’t there other tests we could do?

*Clinician:* I agree, I wouldn’t want to miss anything else. Just like any other diagnosis, we can keep our minds open about the possibilities, but a diagnosis of DGBI makes the most sense for Andy based off what I’m seeing. I don’t have any more tests right now that would help us.

*Caregiver:* Ok…How do you turn off the alarm then? I just really want her to get better.

*Clinician:* *[****REFRAME]*** That’s a great question. It’s going to be a long recovery process, and it’s not always linear, but we know from our scientific studies that the majority of kids do get better with the right support. I think we should focus on what we can do to get back to functioning, back to school for example.

*[****MANAGEMENT]*** The main thing we are trying to do with functional abdominal pain syndrome is rewire the brain-gut connection. We need to create new positive pathways between the brain and gut to turn down the pain volume dial down. We usually come at this from a few different angles, identifying the the biological, psychological, and social aspects that we can work on. Usually, this involves re-establishing routines at school and at home so that the body knows what to anticipate, that the training is happening every day. It also includes follow up with your pediatrician to address any new concerns and monitor her symptoms. We will also get our psychology team to explore more what else may be going on in her life that could be contributing.

As her parent, it is important for you to continue to communicate with us about your concerns. We are your partners in helping her to heal. What questions do you have for us about the information that we have shared?
