# Supplementary material for: INFORM: A Pediatrician's Communication Curriculum About Diagnostic Conversations in Somatic Symptom and Related Disorders
Source: MedEdPORTAL. 2025 Dec 2;21:11561. doi: 10.15766/mep_2374-8265.11561 (PMC12669383; doi:10.15766/mep_2374-8265.11561)
Supplement: Supplementary file 1 — Curriculum Agenda.docxSlide Deck With Script.pptxScript for Case Demonstration by Facilitators.docxCases for Role-Play.docxObserver and Caregiver Guide for Role-Play.docxINFORM Quick Guide.docxGlossary of Acronyms.docxCurriculum Evaluation Forms.docx [file mep_2374-8265.11561-s001.zip › D. Cases for Role-Play.docx]

**INFORM: SSRD Communication Curriculum**

**Case Scenario 1:** Abnormal movements

Mario is a 14-year-old with a history of depression who presents to the ED with 2 episodes of seizure-like activity where both his arms shake uncontrollably. He can hear his mother talking during the episodes but cannot respond. There is no postictal period or incontinence noted. Mother notes increased sleep and irritability recently, and he has missed several school days. 2 months ago, he had a similar episode and was discharged from another hospital with no plan or explanation. His neurological exam is normal. He was admitted overnight for concern for seizures.

*Past medical history:* He has depression and is on a SSRI. Has a history of febrile seizures as child.

*Social* *history*: His boyfriend broke up with him last month. His father would be upset if he knew Mario had a boyfriend. He is spending more time in his room and is on his phone all the time. Mom is worried because she has a sister with epilepsy.

*Neurology Consult*: recommend routine EEG and head MRI, which are both normal. His neurologic exam is normal. You suspect a diagnosis of functional neurologic disorder. Neuro is signing off the case.

**Case Scenario 2**: Abdominal Pain

Amaya is a 14-year-old admitted for intractable abdominal pain and failed PO trial in the ED. This is her third visit to the ED in 6 weeks for similar symptoms. She had viral gastroenteritis 3 months prior, and since then has been complaining of daily abdominal pain, feeling weak and dizzy. Work-up has included CBC with diff, CRP, CMP, lipase, abdominal CT (at outlying ED 2 weeks prior), and abdominal ultrasound, all of which are within normal limits. Head CT in the ED here was normal. She denies fever, loose stools, but has occasional emesis. Her abdomen is diffusely tender to even light tough, but soft when palpated, and she becomes tearful when discussing her pain. She has gained weight since her last check weeks ago. She appears comfortable on exam but complains of headache. She is able to ambulate to the restroom, but she vomited after eating a slice of pizza. Mother refuses to take her home until she has an answer, and she is worried doctors are missing something.

*Past medical history:* Amaya is on Lexapro for depression, sees the school counsellor weekly, and has an IEP at school.

*Social history*: She lives between two households and identifies grandmother as her primary caregiver.

You suspect a diagnosis of disorder of gut-brain-interaction.

**Case Scenario 3**: Weakness

Claire is a 16-year-old girl admitted to the hospital because of intermittent leg weakness, causing her to fall. Symptoms started 3 days ago and have been getting gradually worse. She started feeling a tingling sensation in her legs, and when she got up to go grab water, her legs felt weak, and she needed to sit down. Yesterday, as she was getting ready for soccer practice, she started feeling the tingling again and fell in her bedroom. Her mom rushed to find her, and it took 20 minutes for her to finally get back up and walking again. Her mom decided to bring her to the ED, and before coming, she stopped on the way to buy a wheelchair. In the ED, Claire was unable to get up for gait testing. Her neurologic exam is otherwise normal. There are no visible injuries or pain to her lower extremities. The ED provider ordered a brain/spine MRI, which was normal. Metabolic panel, CBC with diff, and urine pregnancy test are all negative. You are waiting for a neurology consult. You suspect a diagnosis of functional neurologic disorder.

*Past medical history:* Claire suffered from a concussion and right ankle fracture 1 year ago after a soccer accident.

*Social history:* Claire is a straight-A student and is prepping for college applications. She looks a little more tired than usual. She has not been as fast in soccer since her ankle fracture. Her mother has been busy at work and feels guilty she may not have noticed symptoms earlier. Her mom is worried this will have consequences on her college admissions, since she has been missing so much school.
