# Supplementary material for: INFORM: A Pediatrician's Communication Curriculum About Diagnostic Conversations in Somatic Symptom and Related Disorders
Source: MedEdPORTAL. 2025 Dec 2;21:11561. doi: 10.15766/mep_2374-8265.11561 (PMC12669383; doi:10.15766/mep_2374-8265.11561)
Supplement: Supplementary file 1 — Curriculum Agenda.docxSlide Deck With Script.pptxScript for Case Demonstration by Facilitators.docxCases for Role-Play.docxObserver and Caregiver Guide for Role-Play.docxINFORM Quick Guide.docxGlossary of Acronyms.docxCurriculum Evaluation Forms.docx [file mep_2374-8265.11561-s001.zip › E. Observer and Caregiver Guide for Role-Play.docx]

**Observer Guide**

Questions to review during your team’s debrief:

1. Did the clinician follow the INFORM framework? Were there any missing components?
2. What is one thing that the clinician did very well?
3. Where did the clinician seem to struggle most?
4. What analogy did they use? Did it work well for this case?

**Caregiver Guide**

Aim to ask **1-2** of the following questions during your conversation:

- Are you saying this is in their head?
- Are you sure this is what’s going on?
- How should I respond when the symptom is occurring?
- Can the symptom hurt them?
- How will I know if they need more evaluation/testing?
- What if we’re missing something?
- Shouldn’t we run more tests?
- Are we going to stay in the hospital until they are better?
- Will she always have this illness?
