# Supplementary material for: INFORM: A Pediatrician's Communication Curriculum About Diagnostic Conversations in Somatic Symptom and Related Disorders
Source: MedEdPORTAL. 2025 Dec 2;21:11561. doi: 10.15766/mep_2374-8265.11561 (PMC12669383; doi:10.15766/mep_2374-8265.11561)
Supplement: Supplementary file 1 — Curriculum Agenda.docxSlide Deck With Script.pptxScript for Case Demonstration by Facilitators.docxCases for Role-Play.docxObserver and Caregiver Guide for Role-Play.docxINFORM Quick Guide.docxGlossary of Acronyms.docxCurriculum Evaluation Forms.docx [file mep_2374-8265.11561-s001.zip › F. INFORM Quick Guide.docx]

**Quick Guide** -Framework and Scripts for SSRD Diagostic Delivery

**ORIENT: Example scripts for different functional symptoms**


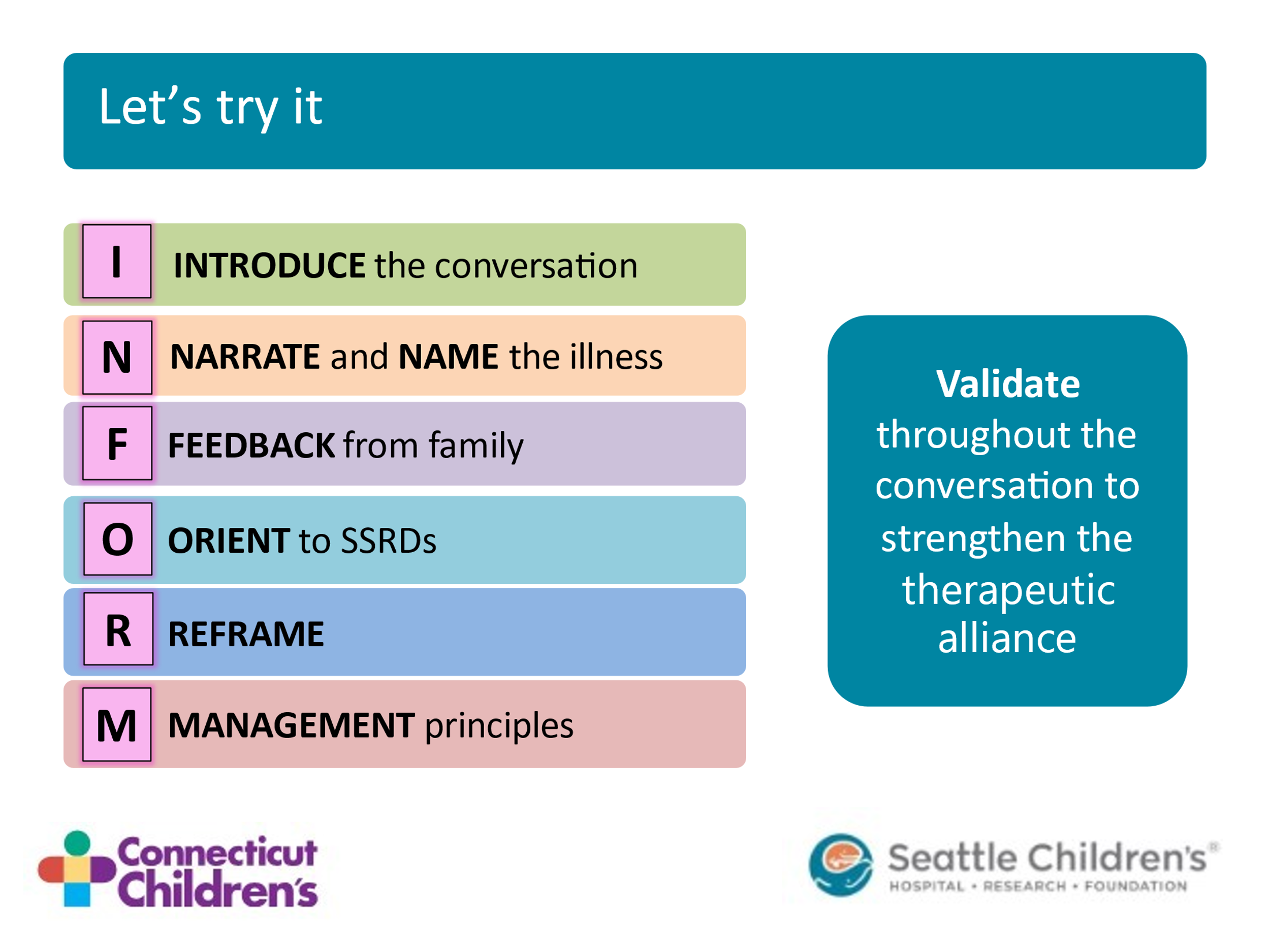
 **INFORM**

**Role of psyche on pain amplification**

“Symptoms tend to worsen in stressful situations or when focusing on the pain, such as when someone asks about the pain.”

“Symptoms can worsen if the child sees that others are also very concerned or anxious in the moment.”

**GI: Nausesa, abdominal pain**

“The brain and the gut are miscommunicating. Even though the gut is healthy, it is sending signals that are interpreted as [pain/nausea] by the brain, like a miswiring.”

**Neurologic:** **numbness, abnormal movements, weakness, inability to walk**

“We can see that his body is strong and reflexes are intact, but his mind and body are not communicating in a healthy way. The body has learned a pattern of [insert symptom: abnormal movements, numbness, paralysis] that is possibly triggered by [insert environmental factor]. This pattern needs to be re-wired, which will take some time.”


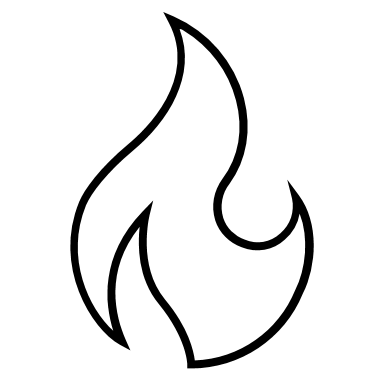

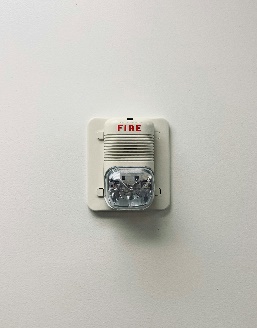
**
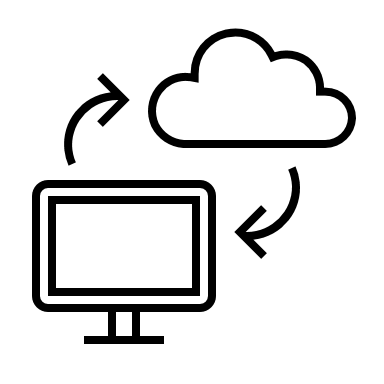
**­­­­

**Alarm** = symptoms, nervous system signaling

**Fire/smoke** = trigger (biological, trauma, etc)

**Validating Scripts**

“There is clearly something wrong.”

“I see that this is really affecting her daily life. It must be hard to not be able to participate in [school, sport, activity].”

“This diagnosis can be frustrating for families because there is not a specific test for this condition”

**Analogies**

**Software** = mind, nervous system signaling, symptoms

**Hardware** = body
