# Supplementary material for: INFORM: A Pediatrician's Communication Curriculum About Diagnostic Conversations in Somatic Symptom and Related Disorders
Source: MedEdPORTAL. 2025 Dec 2;21:11561. doi: 10.15766/mep_2374-8265.11561 (PMC12669383; doi:10.15766/mep_2374-8265.11561)
Supplement: Supplementary file 1 — Curriculum Agenda.docxSlide Deck With Script.pptxScript for Case Demonstration by Facilitators.docxCases for Role-Play.docxObserver and Caregiver Guide for Role-Play.docxINFORM Quick Guide.docxGlossary of Acronyms.docxCurriculum Evaluation Forms.docx [file mep_2374-8265.11561-s001.zip › G. Glossary of Acronyms.docx]

**Glossary of Acronyms**

*For internal use as a reference by facilitators. The following acronyms are used throughout several appendices.*

- ACE -adverse childhood experience
- CBC -complete blood count
- CMP -complete metabolic panel
- CRP -C-reactive protein
- CT -computed tomography
- DGBI -disorder of gut-brain interaction
- ED -emergency department
- EEG -electroencephalogram
- FND -functional neurologic disorder
- FNSD -functional neurologic symptoms disorder
- IEP -individualized education program
- MUPS -medically unexplained physical symptoms
- MRI -magnetic resonance imaging
- PNES -psychogenic nonepileptic seizures
- PO -*per os*
- PT/OT -physical therapy/occupational therapy
- SSRD -somatic symptom and related disorder
- SSRI -selective serotonin reuptake inhibitor
