# Supplementary material for: INFORM: A Pediatrician's Communication Curriculum About Diagnostic Conversations in Somatic Symptom and Related Disorders
Source: MedEdPORTAL. 2025 Dec 2;21:11561. doi: 10.15766/mep_2374-8265.11561 (PMC12669383; doi:10.15766/mep_2374-8265.11561)
Supplement: Supplementary file 1 — Curriculum Agenda.docxSlide Deck With Script.pptxScript for Case Demonstration by Facilitators.docxCases for Role-Play.docxObserver and Caregiver Guide for Role-Play.docxINFORM Quick Guide.docxGlossary of Acronyms.docxCurriculum Evaluation Forms.docx [file mep_2374-8265.11561-s001.zip › H. Curriculum Evaluation Forms.docx]

**Survey - SSRD Communication Workshop**

Dear participant,

Thank you for participating in this workshop! Your answers to these questions are part of a research study at the University of Washington to help guide further curriculum development. Your responses are completely voluntary and anonymous.

This study has been given an exempt status by the Institutional Review Board.

**Pre workshop:**

*Background:* Somatic Symptoms and Related Disorders (SSRD) is an umbrella term that describes multiple diagnoses involving somatization. Some examples include functional abdominal pain, functional neurologic changes (eg numbness, weakness, abnormal movements), or functional joint pain.

1. True/False: It is best practice to wait until all medical workup is complete prior to discussing a SSRD diagnosis with a patient’s family.

- True
- False

1. True/False: When talking about a SSRD diagnosis, it is important to reassure parents by emphasizing that nothing is wrong with their child.

- True
- False

1. True/False: Parent buy-in of the diagnosis improves outcomes for children with SSRDs.

- True
- False

1. Have you ever attended a lecture, workshop, or other educational opportunity throughout your medical training devoted to best practices in communicating an SSRD diagnosis to a patient or their family? *Check one.*

- Yes
- No
- Not sure

1. I feel confident in my ability to deliver a new SSRD diagnosis to a child’s parent. *Circle one.*

Strongly disagree Disagree Neither Agree nor Disagree Agree Strongly Agree

1. It is important to my clinical practice to know how to deliver a SSRD diagnosis. *Circle one.*

Strongly disagree Disagree Neither Agree nor Disagree Agree Strongly Agree

**--------------------***Pause here; complete the remainder of the survey after the workshop***------------------------**

**Post workshop:**

1. True/False: It is best practice to wait until all medical workup is complete prior to discussing a SSRD diagnosis with a patient’s family.

- True
- False

1. True/False: When talking about a SSRD diagnosis, it is important to reassure parents by emphasizing that nothing is wrong with their child.

- True
- False

1. True/False: Parent buy-in of the diagnosis improves outcomes for children with SSRDs

- True
- False

1. I feel confident in my ability to deliver a new SSRD diagnosis to a child’s parent. *Circle one.*

Strongly disagree Disagree Neither Agree nor Disagree Agree Strongly Agree

1. It is important to my clinical practice to know how to deliver a SSRD diagnosis. *Circle one.*

Strongly disagree Disagree Neither Agree nor Disagree Agree Strongly Agree

1. I plan to use what I have learned in today’s workshop in future patient interactions. *Circle one.*

Strongly disagree Disagree Neither Agree nor Disagree Agree Strongly Agree

1. I felt like I had enough time to practice my communication skills in this workshop. *Cirlce one.*

Strongly disagree Disagree Neither Agree nor Disagree Agree Strongly Agree

1. I enjoyed the role-play activity as a way to practice my communication skills. *Circle one.*

Strongly disagree Disagree Neither Agree nor Disagree Agree Strongly Agree

1. I would recommend this workshop to a colleague. *Circle one.*

Strongly disagree Disagree Neither Agree nor Disagree Agree Strongly Agree

1. What did you like most about this workshop? *Write your response below.*
2. Is there anything you would recommend be changed about this workshop? *Write your response below.*
3. Demographic data
4. **Type of clinician/level of training. Check one:**

- Medical student: Year? ______
- Resident: Specialty? ___________________Year?_____
- Fellow: Specialty? ­­­­­_________________ Year? ­­­­_____
- Nurse Practitioner. Specialty? __________________
- Physician Assistant. Specialty? __________________
- Attending. Specialty? __________________

**b. Gender:**

- Female
- Male
- Non-binary
- Other: _________________
- Prefer not to answer

**c. Race (check all that apply):**

- - White/Caucasian
  - Alaska Native
  - Asian
  - Black or African American
  - Native Hawaiian
  - Other Pacific Islander
  - Prefer not to answer

**d. Ethnicity:**

- Hispanic or Latinx
- Not Hispanic or Latinx
- Prefer not to answer
